# Causal relationship between gut microbiota and prostate cancer contributes to the gut-prostate axis: insights from a Mendelian randomization study

**DOI:** 10.1007/s12672-024-00925-1

**Published:** 2024-03-03

**Authors:** Li Wang, Yong-bo Zheng, Shan Yin, Kun-peng Li, Jia-hao Wang, Er-hao Bao, Ping-yu Zhu

**Affiliations:** 1 Department of Urology, Affiliated Hospital of North Sichuan Medical College, Nanchong, China; 2 Department of Urology, The Second Hospital of Lanzhou University, Lanzhou, China

**Keywords:** Mendelian randomization, Prostate cancer, Risk, Gut microbiota, Gene

## Abstract

**Background:**

Changes in gut microbiota abundance have been linked to prostate cancer development. However, the causality of the gut-prostate axis remains unclear.

**Methods:**

The genome-wide association study (GWAS) data for gut microbiota sourced from MiBioGen (n = 14,306), alongside prostate cancer summary data from PRACTICAL (n = 140,254) and FinnGen Consortium (n = 133,164). Inverse-variance-weighted (IVW) was mainly used to compute odds ratios (OR) and 95% confidence intervals (Cl), after diligently scrutinizing potential sources of heterogeneity and horizontal pleiotropy via the rigorous utilization of Cochran's Q test, the MR-PRESSO method, and MR-Egger. We used meta-analysis methods in random effects to combine the Mendelian randomization (MR) estimates from the two sources.

**Results:**

The pooled analyses of MR results show that genus *Eubacterium fissicatena* (OR = 1.07, 95% CI 1.01 to 1.13, P = 0.011) and genus *Odoribacter* (OR = 1.14, 95% CI 1.01 to 1.27, P = 0.025) were positively associated with prostate cancer. However, genus *Adlercreutzia* (OR = 0.89, 95% CI 0.83 to 0.96, P = 0.002), *Roseburia* (OR = 0.90, 95% CI 0.83 to 0.99, P = 0.03), *Holdemania* (OR = 0.92, 95% CI 0.86 to 0.97, P = 0.005), *Flavonifractor* (OR = 0.85, 95% CI 0.74 to 0.98, P = 0.024) and *Allisonella* (OR = 0.93, 95% CI 0.89 to 0.98, P = 0.011) seems to be a protective factor for prostate cancer. Sensitivity analysis found no significant heterogeneity, horizontal pleiotropy, or reverse causal links in all causal associations.

**Conclusion:**

This MR study lends support to a causal relationship between genetically predicted gut microbiota and prostate cancer. Research on the gut-prostate axis, along with further multi-omics analyses, holds significant implications for the prevention and treatment of prostate cancer.

**Supplementary Information:**

The online version contains supplementary material available at 10.1007/s12672-024-00925-1.

## Introduction

In men globally, prostate cancer represents 7% of all new cancer diagnoses, with a pronounced prevalence in Western nations [1]. Notably, it emerges as the second leading cause of cancer-associated mortality in this demographic, culminating in over 350,000 deaths annually [2]. Given this, the urgency of early detection in potentially high-risk individuals, accompanied by swift therapeutic interventions, becomes paramount in curbing both the incidence and mortality rates linked to this malignancy.

The gut microbiota, a diverse collection of microorganisms in the digestive tract, is vital in determining and sustaining the host's health via interactions like nutrient processing and immune system modulation. Obesity and high-fat consumption are linked to Prostate cancer risks, with lifestyle, particularly dietary habits, influencing the gut microbiome [3]. It's long been believed that certain bacteria can cause persistent, mild inflammation, potentially triggering prostate cancer. Although the current positive correlation between prostatitis and prostate cancer rates may be the result of detection bias [4]. Poutahidis et al. [5] demonstrated that gastrointestinal bacterial infections can enhance prostatic intraepithelial neoplasia (PIN) and microinvasive carcinoma in vivo. Additionally, individuals diagnosed with prostate cancer showed significant increases in proinflammatory Bacteroides and Streptococcus species. Antibiotics promote selection for resistant bacteria by enhancing the proliferation of pathogenic strains. Research indicates that antibiotic use elevates the risk of infections from *Clostridium difficile* and methicillin-resistant *Staphylococcus aureus* [6]. Tulstrup et al. [7] found that changes in the microbiota due to antibiotics can alter intestinal permeability, thereby heightening the risk of neoplastic alterations. Earlier research has demonstrated that prostate cancer patients exhibiting elevated oestrogen levels may possess intestinal bacterial genes capable of oestrogen metabolism. Such metabolic activity can expedite carcinogenesis by activating polycyclic aromatic hydrocarbons (PAHs) [8–10]. *Escherichia coli* commonly resides in the human gut. Murine studies have indicated a potential link between E. coli and prostatitis development [11]. Moreover, *Campylobacter jejuni* has been identified as an inducer of cell cycle arrest and cellular death through its toxin production. Notably, *Clostridium* can transform gut glucocorticoids into androgens through side chain cleavage, contributing synergistically to the progression of prostate cancer [12]. While numerous studies have investigated the link between specific gut microbes and prostate cancer, the causal relationship between them remains unclear [13, 14].

Mendelian randomization (MR) emerges as a method of instrumental variable (IV) analysis that harnesses single nucleotide polymorphisms (SNPs) derived from genome-wide association studies (GWAS) as tools to deduce causal associations between two traits [15]. MR approximates the inherent attributes of a RCT and exhibits a reduced susceptibility to the impact of covariates. Moreover, its operational simplicity and cost-effectiveness enhance its appeal [16]. Consequently, we conducted a two-sample MR utilizing aggregated data from accessible GWAS repositories. This approach facilitated an exploration of the conceivable etiological correlation between gut microbiota and the risk of Prostate cancer through a comprehensive meta-analysis.

## Methods

### Study design

**Fig. 1:**
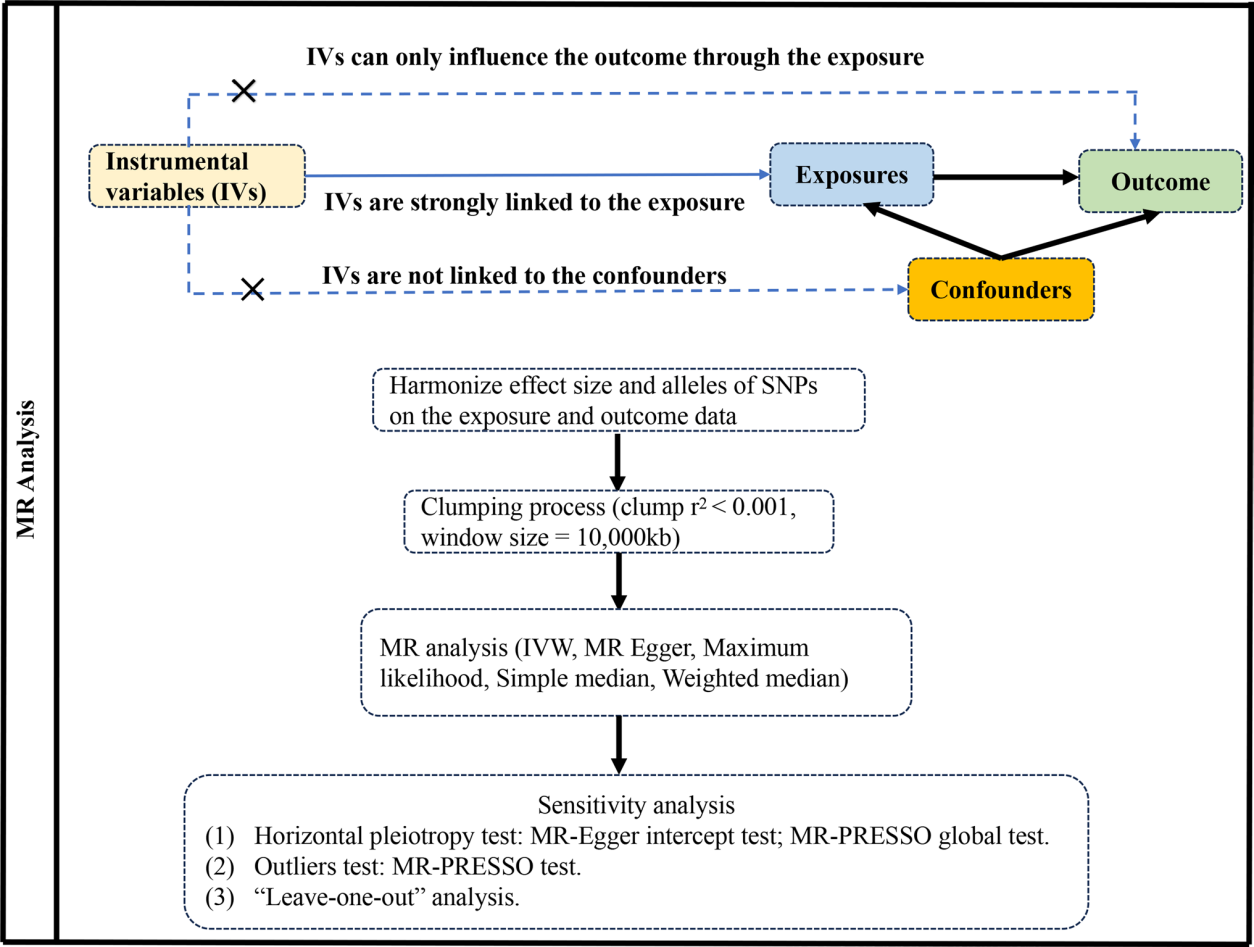
Flowchart of a MR study

The study rigorously adhered to the guidelines outlined in the Strengthening the Reporting of Observational Studies in Epidemiology Mendelian Randomization (STROBE-MR) framework [17]. MR relies on three essential assumptions: IVs demonstrate strong correlation with exposure factors, remain unaffected by confounding variables, and impact outcomes solely through the exposure under investigation [18]. We conducted two-sample MR analyses using summary statistics from genome-wide association studies (GWAS) to investigate the connections between the gut microbiome and prostate cancer. The basic assumptions and MR design flow are depicted in Fig. 1. Since publicly available pooled data were utilized, ethical approval was not necessary for this study.

### Data sources

We obtained SNPs associated with gut microbial abundance from the MiBioGen consortium’s GWAS study [19]. This extensive study involved 25 cohorts, comprising a total of 18,340 participants from diverse ethnic backgrounds. Our dataset included 211 taxa with an average abundance exceeding 1%. After excluding 15 taxa from unidentified groups, we finally used 196 bacterial taxa from 14,306 populations of European ancestry for the MR analysis.

Summary data for prostate cancer were obtained from the comprehensive GWAS meta-analysis conducted by the PRACTICAL consortium [20], encompassing 79,148 cases and 61,106 controls of European descent. Additionally, for validation, we acquired a dataset related to prostate cancer from the FinnGen consortium [21], comprising 13,216 prostate cancer patients and 119,948 controls (Table 1).

**Table 1:** Phenotypic descriptive statistics of studies included in the exposure and outcome genome-wide association studies

Data source	Phenotype	Sample size	Cases	Population	Adjustment	PubMed ID/website
MiBioGen consortium	Gut microbial	14,306	–	European	Age, sex, technical covariates, and genetic principal components	33462485
PRACTICAL	Prostate cancer	140,254	79,148	European	Age, sex	29892016
FinnGen	Prostate cancer	133,164	13,216	European	Age, sex	https://r9.finngen.fi/

PubMed ID: PubMed identifier

### Instrument selection

To ensure the stability of the causal relationship between exposure and outcome, IVs were selected based on the following principles: (1) we established genome-wide significance thresholds for bacterial taxa at p < 1 × 10^–5^. (2) Cluster analysis was conducted to address linkage disequilibrium (LD) among the selected IVs (r^2^ < 0.001, kb = 10,000). (3) Only SNPs with a minor allele frequency (MAF) exceeding 0.01 were considered. (4) To mitigate bias from weak IVs, the strength of the IVs was quantified using the F value (β^2^/SE), with those having F < 10 being excluded [22]. Here, β represents the effect size of exposure and SE represents the standard error of the effect size. We also used PhenoScanner to examine potential confounders (such as body mass index and family history) of exposed SNPs to eliminate effects on outcome. Ultimately, we selected SNPs meeting all the criteria as IVs for downstream MR analysis. The IV selection process is illustrated in Fig. 1.

### Statistical analyses

The primary analysis employed the robust inverse-variance weighted (IVW) method [23]. This method has the strongest statistical efficacy, but it must be satisfied that all genetic variation is a valid instrumental variable, and therefore we employed the weighted median, MR-Egger regression, maximum likelihood and simple weighted mode methods as validation approaches. The weighted median allows for up to 50% of the weights in the estimator to be from invalid instruments [24]. To address potential directional pleiotropy, MR-Egger regression and weighted mode methods were employed [25, 26]. The median-based method evaluates the causal link by focusing on the subset with the highest number of SNPs, and the maximum likelihood method helped assess population overlap.

Sensitivity analysis assumes a vital role in the assessment of heterogeneity and potential biases within MR studies. Firstly, heterogeneity was evaluated through the application of Cochran's Q test, which involved calculating the weighted sum of squared differences between specific variability estimates and the overall IVW estimate [27]. To address potential outliers, the MR Pleiotropy RESidual Sum and Outlier (MR-PRESSO) method was employed during data analysis [28]. Furthermore, MR-Egger regression was utilized, and intercepts were assessed to identify potential horizontal pleiotropy (p < 0.05 was judged significant). In addition, we performed a leave-one-out analysis to test the stability of the results. We evaluated heterogeneity among variant-specific causal estimates and pinpointed outliers through scatter and funnel plots. Finally, we identified potential bidirectional links between SNPs related to the gut microbiota and prostate cancer using the MR Steiger Filtering Test [29].

We conducted MR analysis using the FinnGen consortium dataset for validation and then merged MR estimates from both FinnGen and PRACTICAL datasets through meta-analysis. Statistical analyses were executed using R version 4.2.2 with the “TwoSampleMR,” “meta,” and “MRPRESSO” packages. Odds ratios (ORs) with 95% confidence intervals (CIs) were used to quantify the MR analysis, and statistical significance was defined as P < 0.05.

## Results

### Selection of instrumental variables

We selected 13,860 SNPs with locus-wide significance (P < 1 × 10^–5^) based on 196 bacterial features in the MiBioGen consortium. SNPs with a minor allele frequency ≤ 0.01 were excluded. The linkage disequilibrium threshold was set at r^2^ = 0.001, with a clumping distance of 10,000 kb. We obtained 102, 178, 215, 375, and 1381 SNPs at the phylum, class, order, family, and genus levels, respectively. Namely, 2251 SNPs were selected as IVs. Importantly, all the included SNPs had F-values exceeding 10, indicating a minimal likelihood of weak IVs bias. The screening through PhenoScanner did not reveal interference from confounding factors (Additional file 2: Tables S1–S2).

### MR analyses

**Fig. 2:**
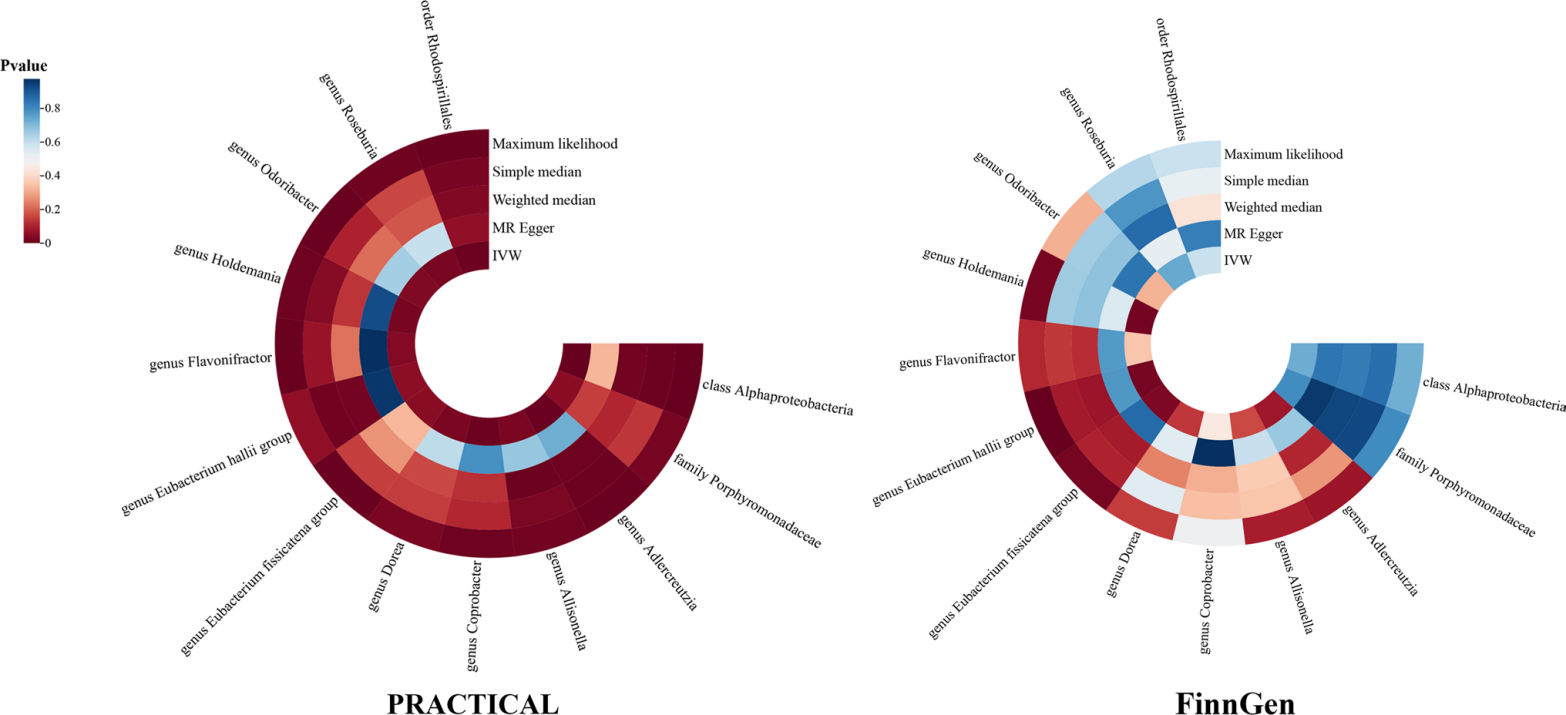
Association of gut microbiome with the risk of prostate cancer

**Fig. 3:**
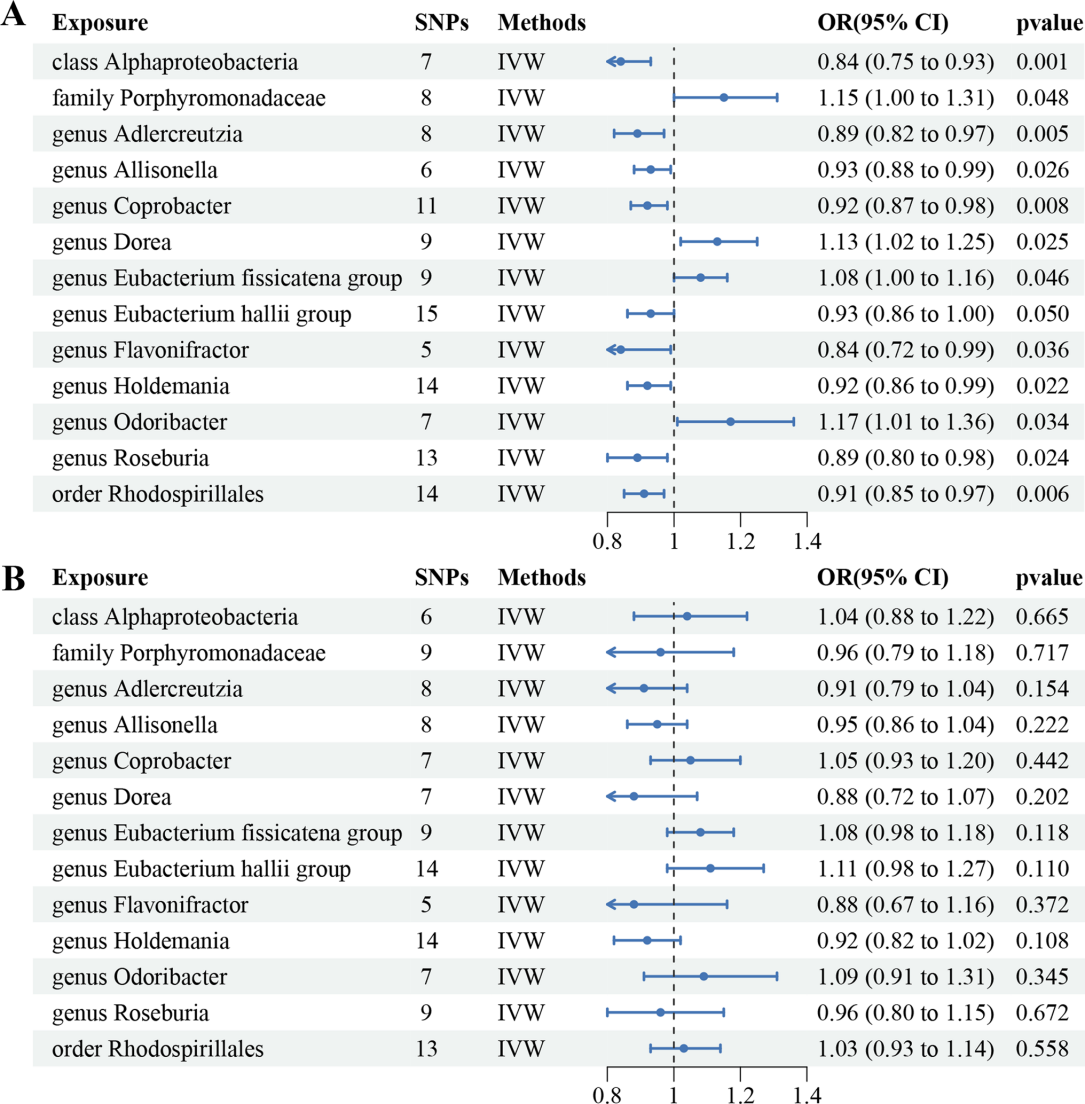
Causal analysis of gut microbiome and prostate cancer based on MR analyses. **A** Based on PRACTICAL consortium, **B** based on FinnGen consortium

**Fig. 4:**
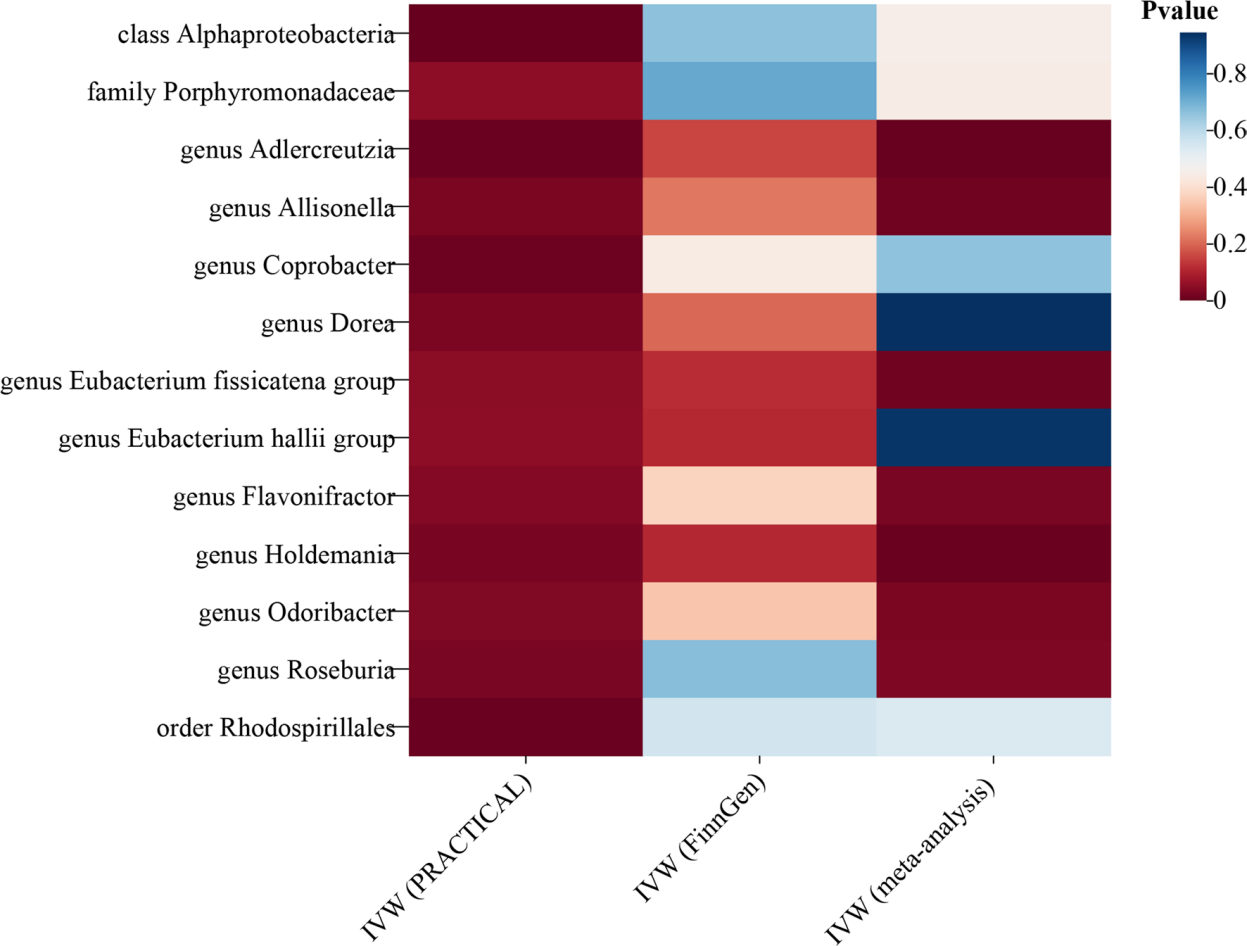
Cumulative analysis of PRACTICAL and FinnGen databases predicts gut flora and prostate cancer risk

Analyzing data from the PRACTICAL consortium, the IVW results indicate that within the class *Alphaproteobacteria* (OR = 0.84, 95% CI 0.75 to 0.93, P = 0.001), genus *Adlercreutzia* (OR = 0.89, 95% CI 0.82 to 0.97, P = 0.005), genus *Eubacterium hallii* (OR = 0.93, 95% CI 0.86 to 1.0, P = 0.05), genus *Roseburia* (OR = 0.89, 95% CI 0.8 to 0.98, P = 0.024), genus *Holdemania* (OR = 0.92, 95% CI 0.86 to 0.99, P = 0.023), genus *Flavonifractor* (OR = 0.84, 95% CI 0.72 to 0.99, P = 0.037), genus *Allisonella* (OR = 0.93, 95% CI 0.88 to 0.99, P = 0.026), genus *Coprobacter* (OR = 0.92, 95% CI 0.87 to 0.98, P = 0.008), and the order *Rhodospirillales* (OR = 0.91, 95% CI 0.85 to 0.97, P = 0.006) demonstrated protective effects against prostate cancer, while family *Porphyromonadaceae* (OR = 1.15, 95% CI 1.0 to 1.31, P = 0.048), genus *Eubacterium fissicatena* (OR = 1.08, 95% CI 1.0 to 1.16, P = 0.046), genus *Odoribacter* (OR = 1.17, 95% CI 1.01 to 1.36, P = 0.033), genus *Dorea* (OR = 1.17, 95% CI 1.01 to 1.36, P = 0.033) were risk factors for prostate cancer. Other supplementary methods in MR analysis corroborate comparable trends and findings regarding the influence of gut microbiota on prostate cancer (Fig. 2; Fig. 3A; Additional file 2: Table S3). MR analyses conducted using the FinnGen database did not reveal a statistically significant causal relationship between gut microbiota and prostate cancer (Fig. 2**; **Fig. 3B**;** Additional file 2: Table S4). We combined MR estimates from both the PRACTICAL and FinnGen databases by meta-analysis and found that genus *Eubacterium fissicatena* (OR = 1.07, 95% CI 1.01 to 1.13, P = 0.011) and genus *Odoribacter* (OR = 1.14, 95% CI 1.01 to 1.27, P = 0.025) were positively associated with Prostate cancer. However, the pooled analysis revealed that genus *Adlercreutzia* (P = 0.002), genus *Roseburia* (P = 0.03), genus *Holdemania* (P = 0.005), genus *Flavonifractor* (P = 0.024) and genus *Allisonella* (P = 0.011) showed suggestive associations with a reduced risk for prostate cancer (Fig. 4; Additional file 2: Table S5).

The MR Steiger filtering test found no reverse causal link between the bacterial taxa and prostate cancer (Additional file 2: Table S6). Cochran's Q test results indicated the absence of statistically significant heterogeneity among these IVs. Moreover, both the Egger Intercept test and the MR-PRESSO Global test failed to identify significant horizontal pleiotropy (Table 2). Visual scatter and funnel plots are available in Additional file 1: Figs. S1–S104. Finally, Leave-one-out analyses confirmed result stability.

**Table 2:** Evaluation of heterogeneity and directional pleiotropy using different methods

Data source	Classification	Bacterial taxas	Heterogeneity			Horizontal pleiotropy			
			I^2^ (%)	IVW		Egger intercept	SE	P-value	MR-PRESSO global test p
				Cochran’s Q	P-value				
PRACTICAL	class	Alphaproteobacteria id.2379	26.131	8.122	0.229	0.005	0.020	0.780	0.312
PRACTICAL	family	Porphyromonadaceae id.943	25.235	9.362	0.227	− 0.019	0.016	0.276	0.256
PRACTICAL	genus	Adlercreutzia id.812	0	6.638	0.467	− 0.004	0.017	0.825	0.489
PRACTICAL	genus	Eubacterium fissicatena group id.14373	46.172	14.862	0.061	− 0.018	0.026	0.503	0.083
PRACTICAL	genus	Eubacterium hallii group id.11338	0	11.601	0.638	− 0.005	0.006	0.380	0.630
PRACTICAL	genus	Odoribacter id.952	43.093	10.543	0.103	0.002	0.019	0.902	0.149
PRACTICAL	genus	Roseburia id.2012	28.276	16.730	0.160	− 0.002	0.011	0.841	0.180
PRACTICAL	genus	Holdemania id.2157	23.979	17.100	0.194	− 0.007	0.010	0.494	0.212
PRACTICAL	genus	Flavonifractor id.2059	49.970	7.995	0.091	− 0.014	0.030	0.661	0.153
PRACTICAL	genus	Allisonella id.2174	18.176	6.110	0.295	0.003	0.028	0.905	0.353
PRACTICAL	genus	Dorea id.1997	0	5.752	0.674	0.003	0.010	0.758	0.707
PRACTICAL	genus	Coprobacter id.949	0	7.946	0.634	− 0.012	0.011	0.305	0.675
PRACTICAL	order	Rhodospirillales id.2667	15.006	15.295	0.289	0.021	0.014	0.149	0.323
FinnGen	class	Alphaproteobacteria id.2379	0	2.982	0.702	− 0.005	0.026	0.836	0.718
FinnGen	class	Porphyromonadaceae id.943	0	1.454	0.993	0.003	0.026	0.904	0.994
FinnGen	genus	Adlercreutzia id.812	0	5.722	0.572	0.005	0.027	0.838	0.602
FinnGen	genus	Eubacterium fissicatena group id.14373	0	6.353	0.607	< 0.001	0.030	0.999	0.615
FinnGen	genus	Eubacterium hallii group id.11338	5.595	13.770	0.390	0.004	0.011	0.699	0.432
FinnGen	genus	Odoribacter id.952	0	3.969	0.680	< 0.001	0.021	0.977	0.738
FinnGen	genus	Roseburia id.2012	0	4.406	0.818	− 0.026	0.019	0.204	0.789
FinnGen	genus	Holdemania id.2157	4.738	13.646	0.399	0.002	0.016	0.898	0.433
FinnGen	genus	Flavonifractor id.2059	52.628	8.443	0.076	0.012	0.052	0.824	0.111
FinnGen	genus	Allisonella id.2174	19.936	8.743	0.271	0.021	0.047	0.666	0.294
FinnGen	genus	Dorea id.1997	0	5.767	0.449	0.017	0.017	0.371	0.475
FinnGen	genus	Coprobacter id.949	0	2.601	0.856	− 0.017	0.041	0.696	0.846
FinnGen	order	Rhodospirillales id.2667	0	5.500	0.939	0.010	0.020	0.622	0.938

MR PRESSO: Mendelian randomization pleiotropy residual sum and outlier

## Discussion

In this study, large-scale GWAS data using European ancestry, combined with MR and meta-analyses demonstrated a potential causal link between gut microbiome and prostate cancer.

Despite the anatomical distance between the prostate and the gut, a substantial body of research suggests a potential link between the gut microbiome and both prostate cancer development and drug resistance. Liss et al. [30] conducted a study utilizing 16S rRNA sequencing to analyze the gut microbiota of 133 American men who underwent prostate biopsies. Their findings indicated elevated levels of *Streptococcus* and *Bacteroides* species in men diagnosed with prostate cancer. Further genome studies indicate that alterations in folate and arginine pathways, possibly influenced by gut microbes, may play a role in prostate cancer risk. Golombos et al. [13] observed a greater prevalence of *Bacteroides massiliensis* in individuals with prostate cancer in comparison to the healthy control group. Conversely, *Faecalibacterium prausnitzii* and *Eubacterium rectalie* exhibited higher relative abundances among the control group. Elevation of *F.prausnitzii* and *E.rectalie* is associated with the formation of anti-inflammatory butyrate, resulting in a symbiotic and protective effect [31, 32].

The results of data pooled from the PRACTICAL and FinnGen consortiums indicate that the genus *Eubacterium fissicatena* and *Odoribacter* are associated with an increased risk of prostate cancer. Conversely, the genus *Adlercreutzia*, *Roseburia*, *Holdemania*, *Flavonifractor*, and *Allisonella* are potential protective factors against prostate cancer. In fact, the gut microbiome tends to be influenced by host genetics. Xu et al. [33] demonstrated that *Odoribacter* had nominally significant heritability estimates (0.476), implying its potential role as a genetic carcinogenic factor for prostate cancer. The *Eubacterium fissicatena* group may be associated with in vivo metabolism. Nutritional investigations have shown a significant increase in the abundance of the *E. fissicatena* group in populations following a low-calorie diet for 6 days a week [34]. Despite the absence of specific studies on the relationship between the *E. fissicatena* group and prostate cancer, Zang et al. discovered a causal relationship between *E. fissicatena* and psoriasis. This finding suggests that gut microbes play a role in mediating the modulation of relevant immune responses [35].

Equol, a secondary metabolite of daidzein produced by the intestinal microbiota, is significantly associated with a reduced risk of prostate cancer in Japanese men, as indicated by plasma equol levels in a study [36]. Additionally, a positive correlation was detected between the genus *Adlercreutzia* and S-equol concentration [37]. *Roseburia*, a Gram-positive anaerobic bacterium, induces cancer cell apoptosis through the inhibition of histone deacetylases and related signaling pathways. It also contributes to immune homeostasis and has anti-inflammatory properties by producing short-chain fatty acids [38]. While research on the role of Holdemania, Flavonifractor, and Allisonella in prostate cancer is limited, their correlation with colorectal cancer and metabolism suggests potential roles in immune function, inflammation, and hormone levels. These factors have been implicated in the development and progression of prostate cancer [39, 40]. The effects of bacteria on prostate cancer risk are likely multifactorial, involving a combination of specific microbial activities, host responses, and interactions within the broader microbiome [41]. Eubacteriales from the same order may have different effects on prostate cancer, which is related to the fact that different species in the same bacterial order may have different metabolic pathways and produce different metabolites. Secondly, the functions of bacteria will vary according to the host and environment, and finally we cannot ignore the interactions between bacterial groups [42]. As research in this field progresses, a more nuanced understanding of these complexities will likely emerge.

### Strength and limitation

Our MR analysis has the following advantages. Firstly, the sample size in the GWAS was large and the study strictly adhered to the three assumptions of MR, thus reducing confounders and reverse bias. Secondly, the study population included only individuals of European origin, minimizing population stratification interference. Finally, sensitivity analyses and different model estimations were used to ensure the reliability of the results.

However, certain limitations are unavoidable. Firstly, we assumed of a linear relationship between gut microbiota and prostate cancer risk, disregarding the potential presence of U-shaped associations. Furthermore, the generalizability of our results to different racial groups and various subtypes of prostate cancer is uncertain. Additionally, data limitations such as individual dietary habits and environmental factors may lead to confounding factors. Consequently, further molecular experiments are imperative to corroborate the findings of this study.

## Conclusion

This MR study unveils genetic evidence supporting a causal link between gut microbiota and prostate cancer. The multi-omics-based platform is anticipated to offer fresh perspectives on prostate cancer diagnosis and treatment by delving into the pathogenic mechanisms and potential bacterial biomarkers.

### Supplementary Information

**Additional file1: Additional figures.**

**Additional file 2: Additional tables.**

## Data Availability

All datasets in this study are available for download in the online dataset/supplementary file and further contact the corresponding author if necessary.
