# Supplementary material for: Causal relationship between gut microbiota and prostate cancer contributes to the gut-prostate axis: insights from a Mendelian randomization study
Source: Discov Oncol. 2024 Mar 3;15:58. doi: 10.1007/s12672-024-00925-1 (PMC10909808; doi:10.1007/s12672-024-00925-1)
Supplement: Supplementary file 1 — Additional file1: Additional figures. [file 12672_2024_925_MOESM1_ESM.pdf]

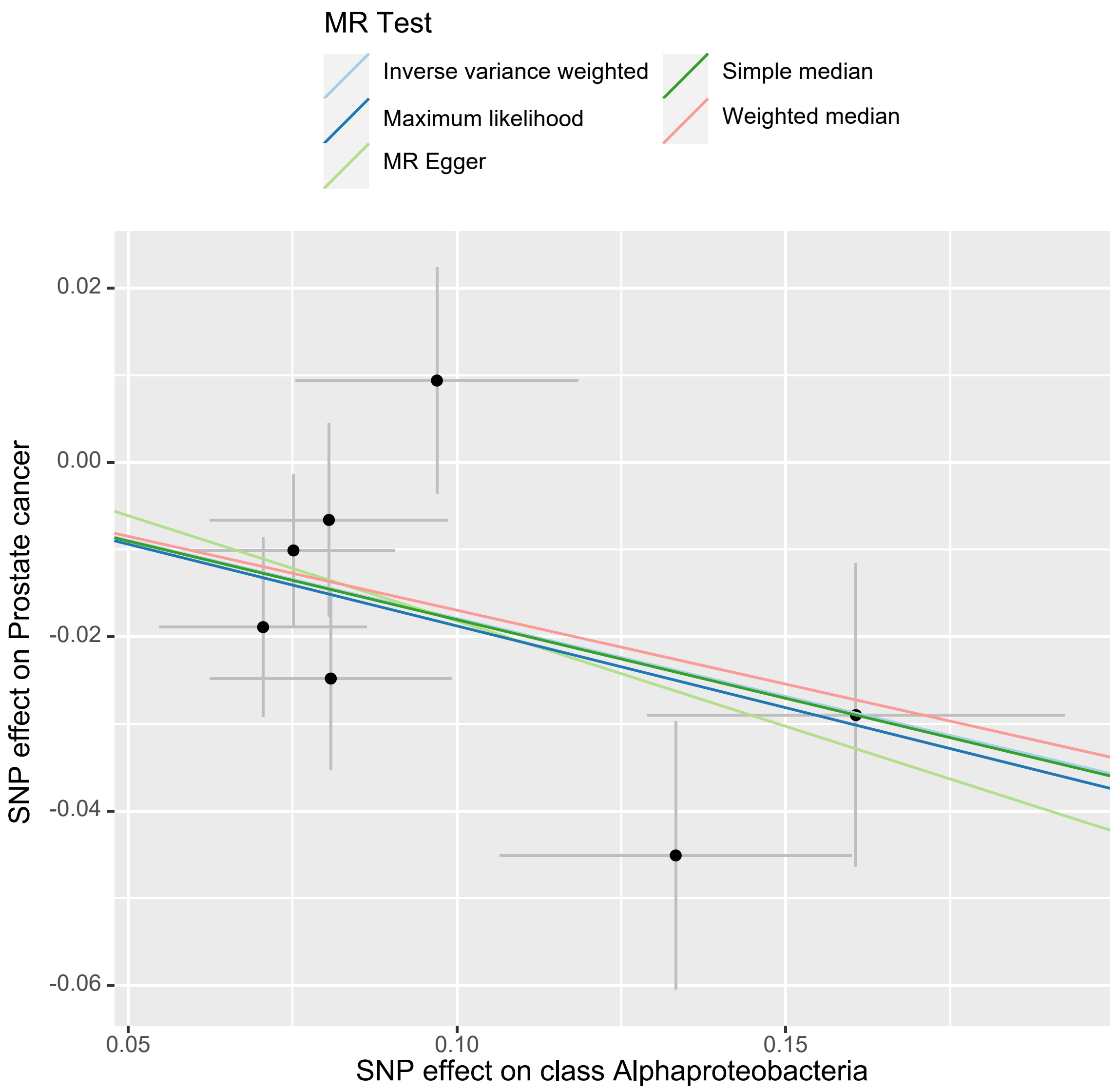

Figure S1. The scatter plot of class Alphaproteobacteria on prostate cancer in the PRACTICAL consortium

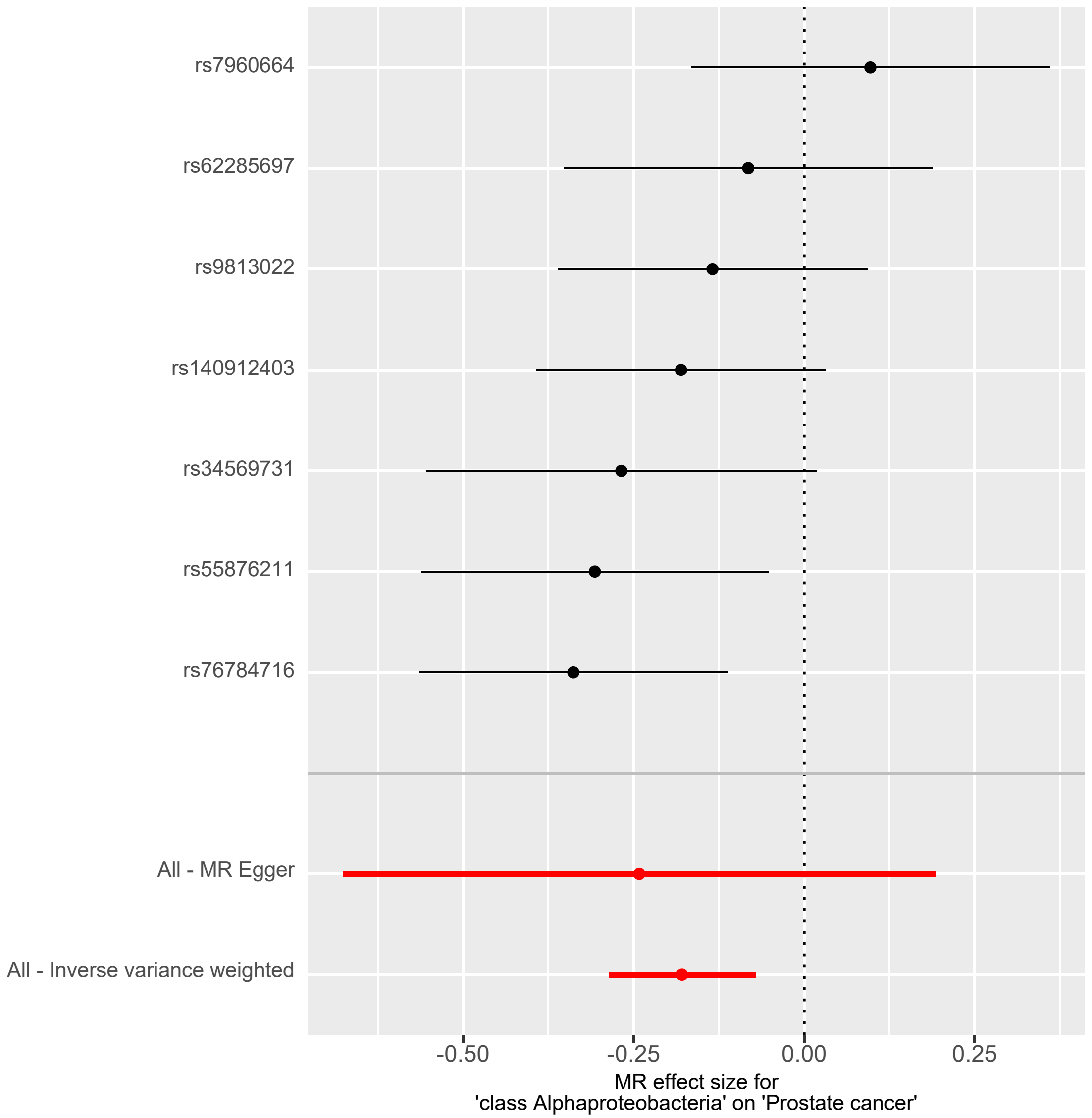

Figure S2. The forest plot of class Alphaproteobacteria on prostate cancer in the PRACTICAL consortium

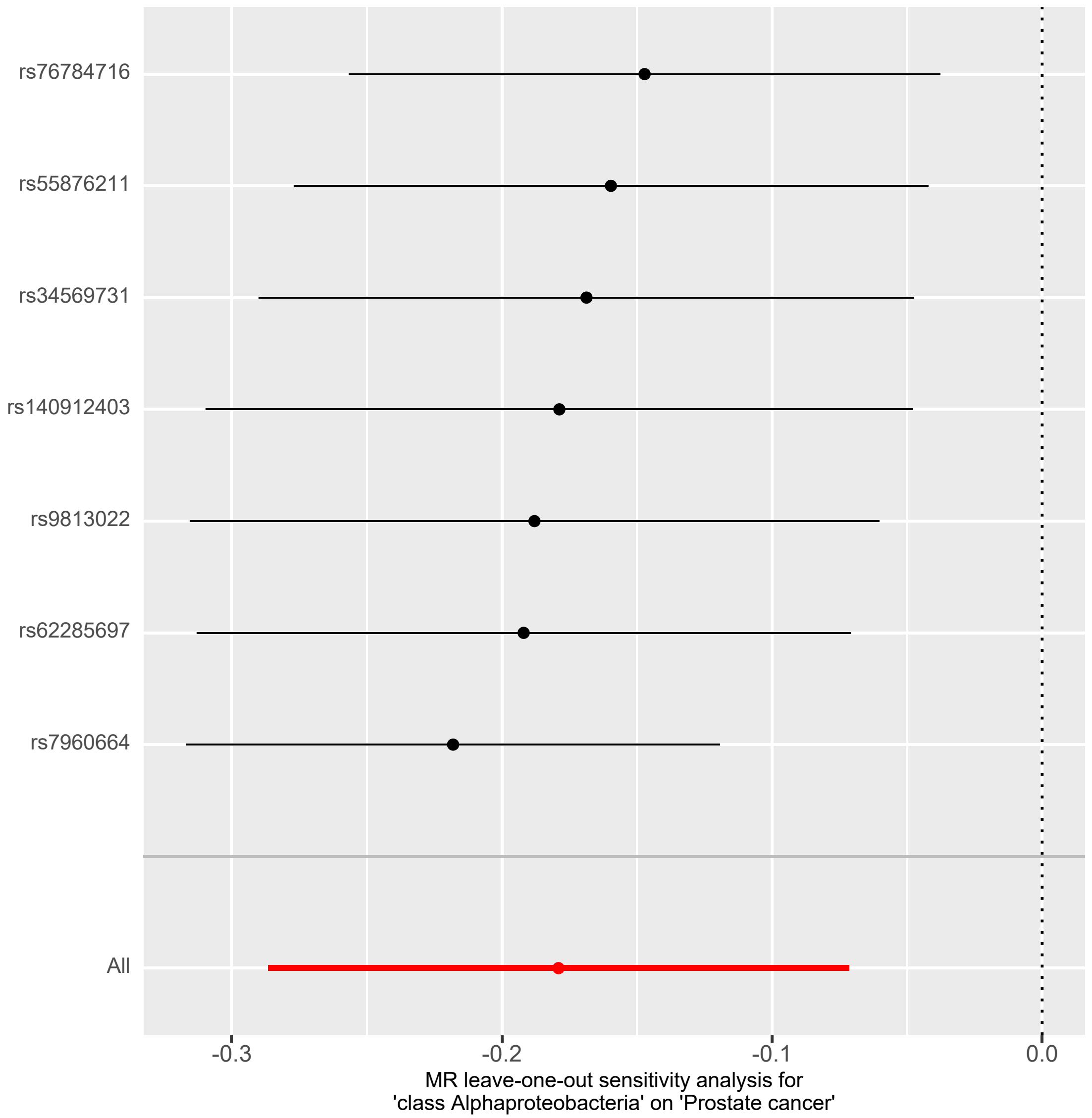

Figure S3. The leave-one-out test of class Alphaproteobacteria on prostate cancer in the PRACTICAL consortium

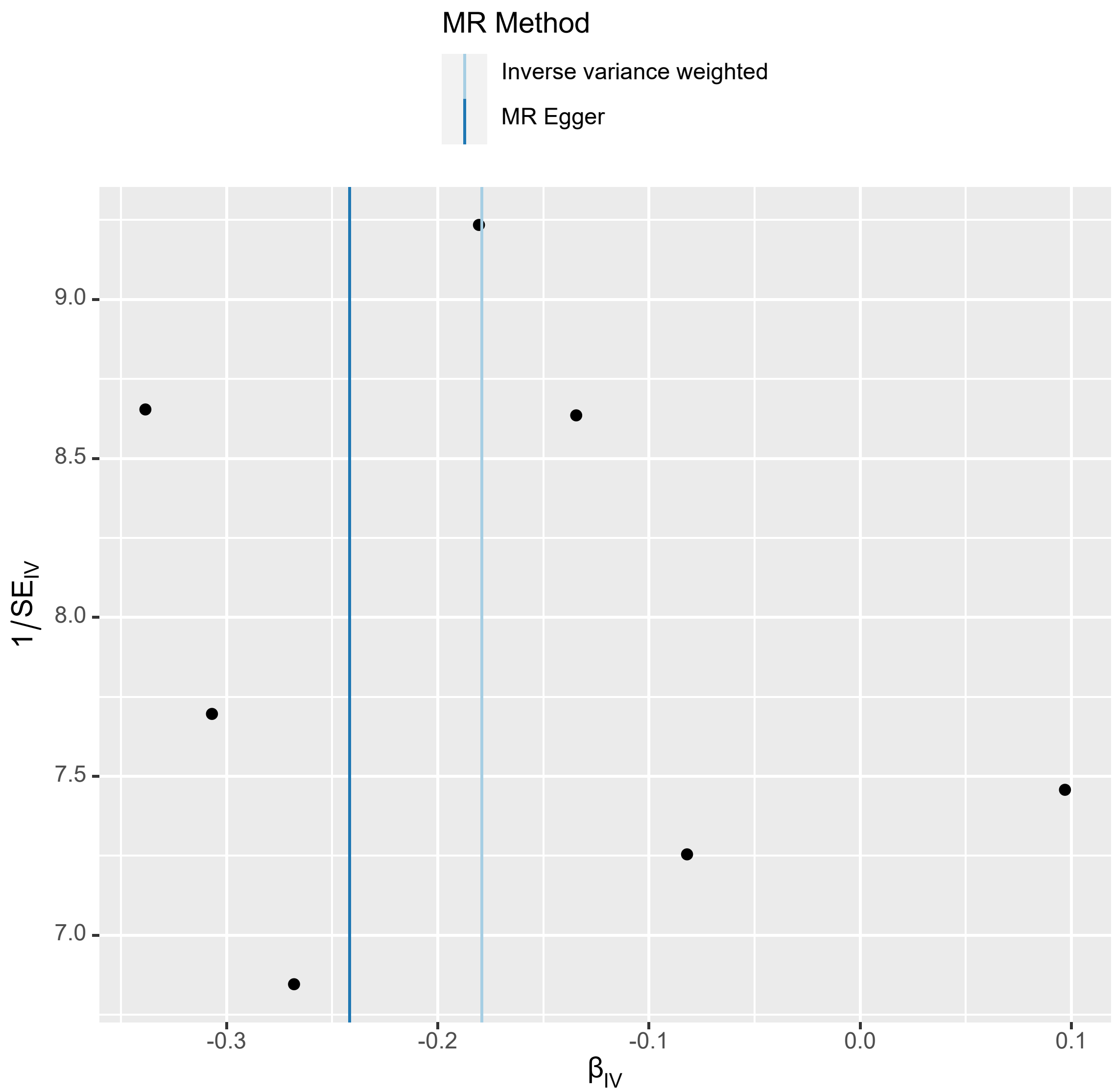

Figure S4. The funnel plot of class Alphaproteobacteria on prostate cancer in the PRACTICAL consortium

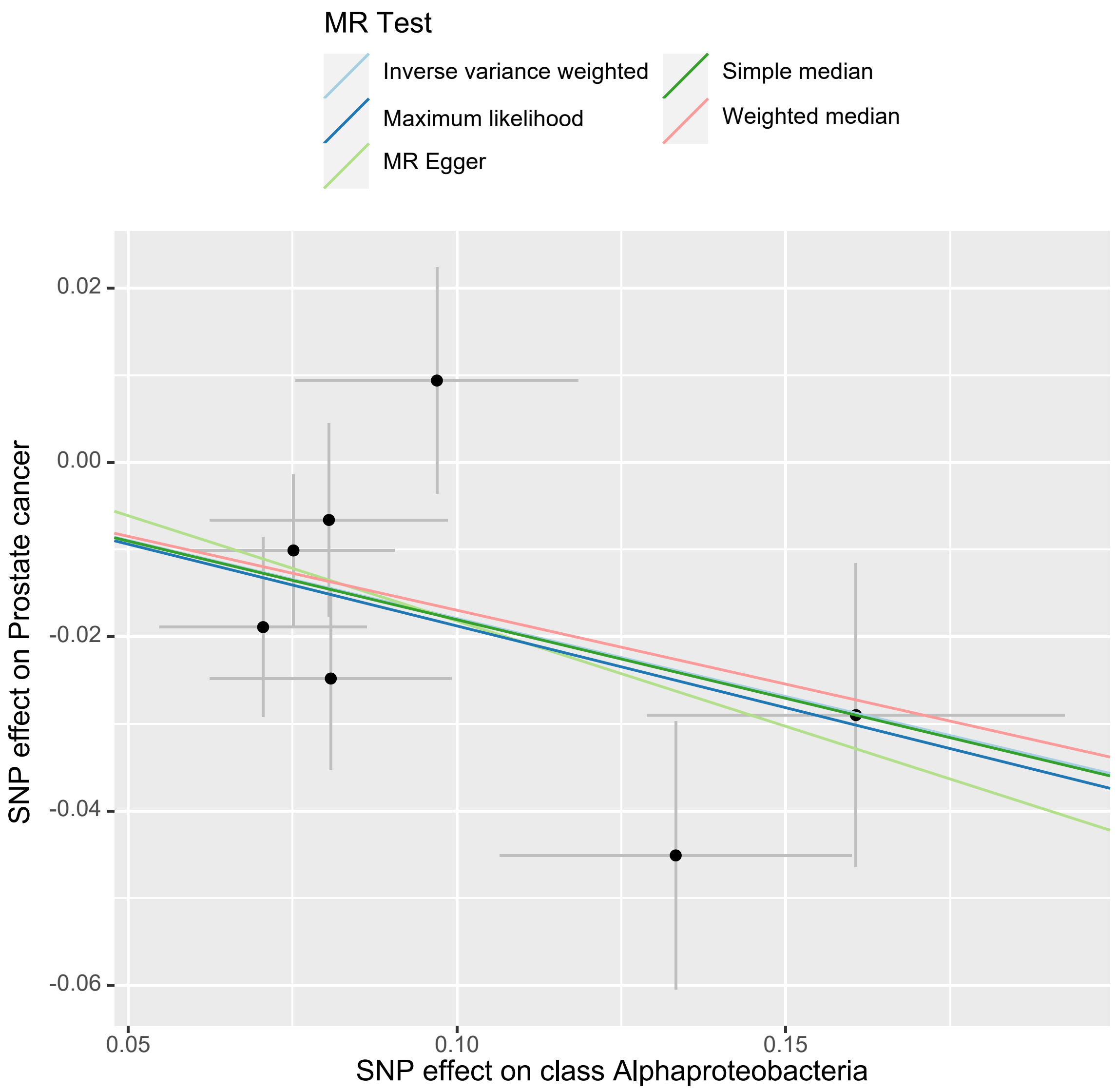

Figure S5. The scatter plot of family Porphyromonadaceae on prostate cancer in the PRACTICAL consortium

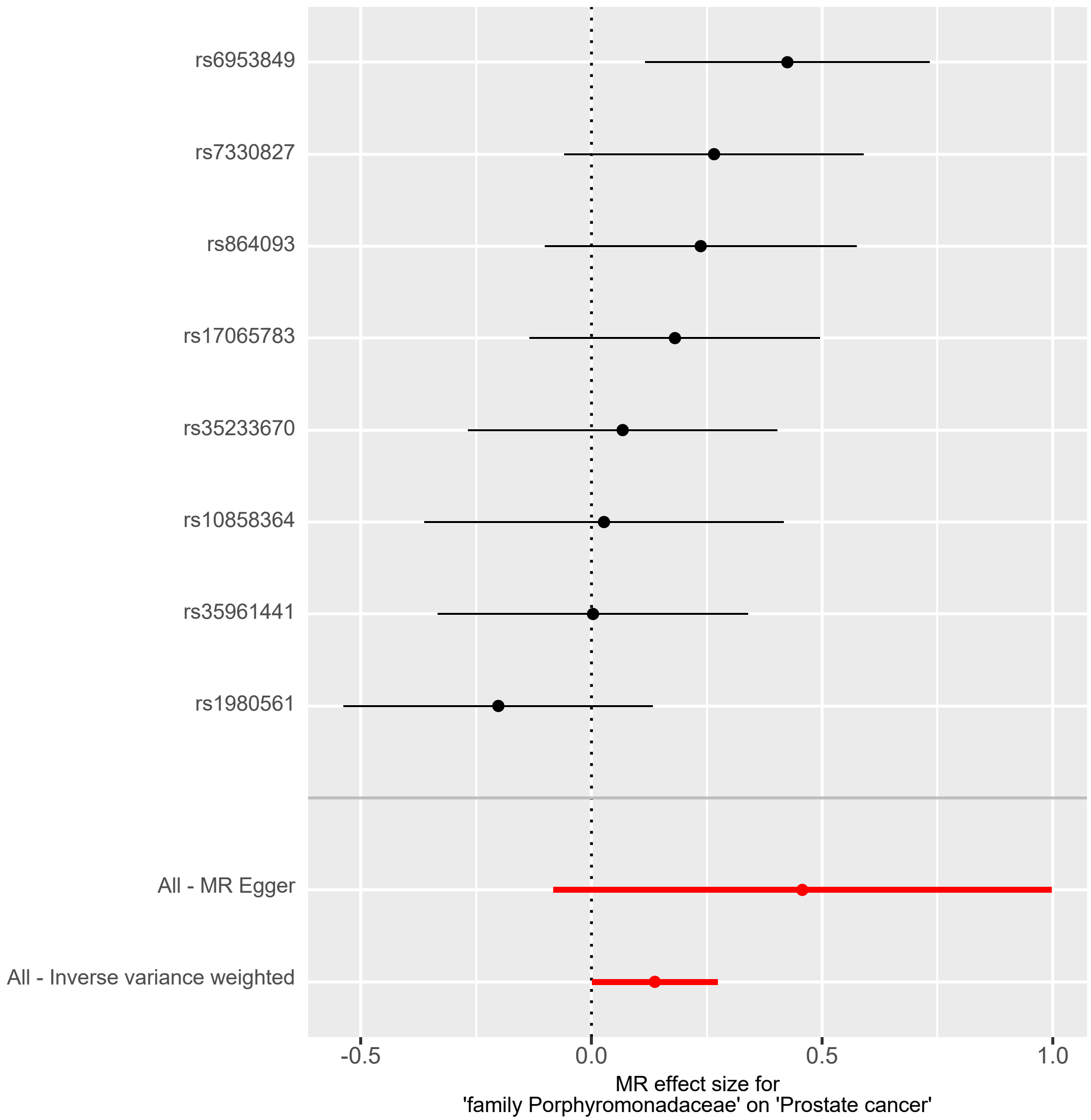

Figure S6. The forest plot of family Porphyromonadaceae on prostate cancer in the PRACTICAL consortium

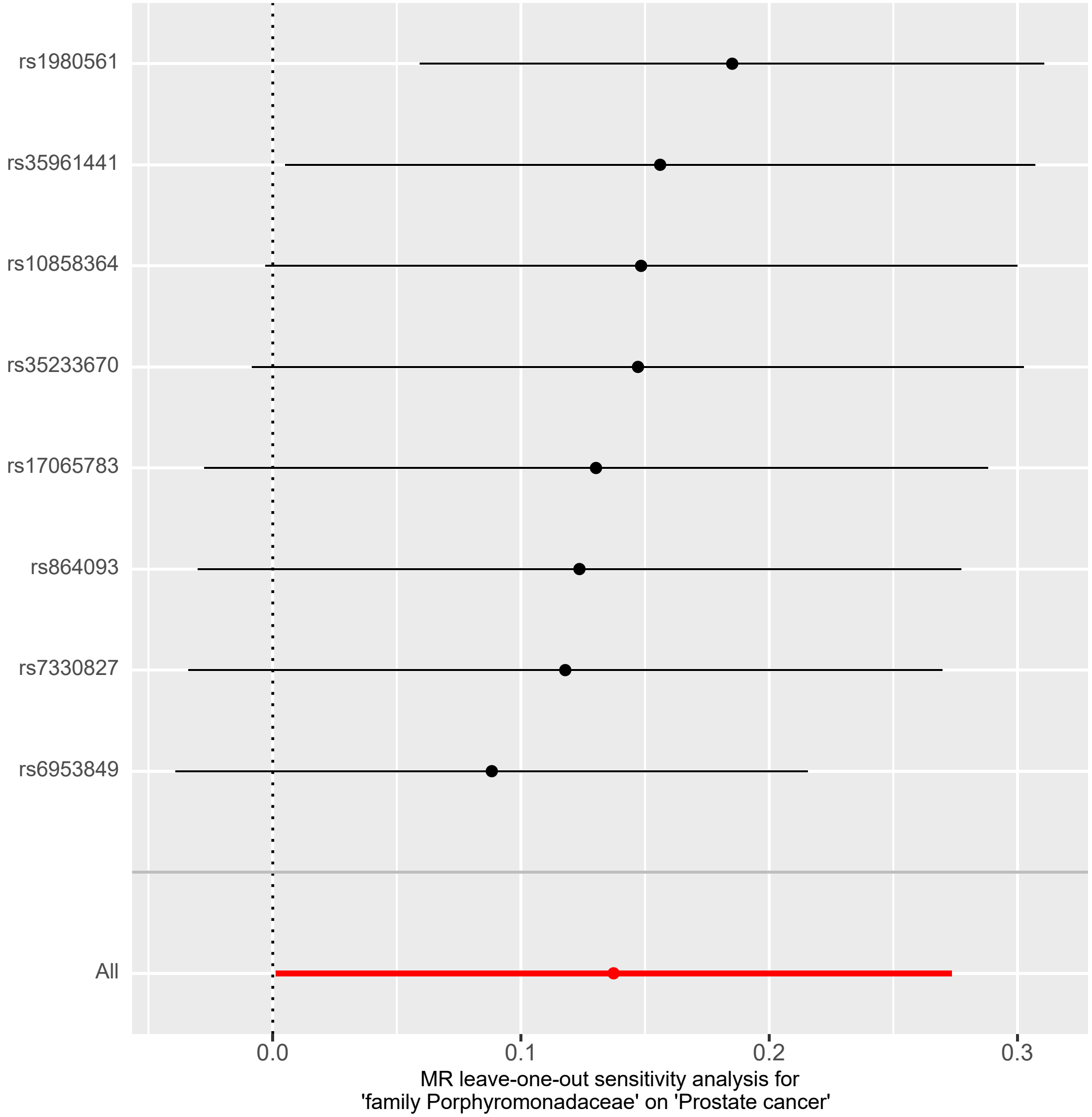

Figure S7. The leave-one-out test of family Porphyromonadaceae on prostate cancer in the PRACTICAL consortium

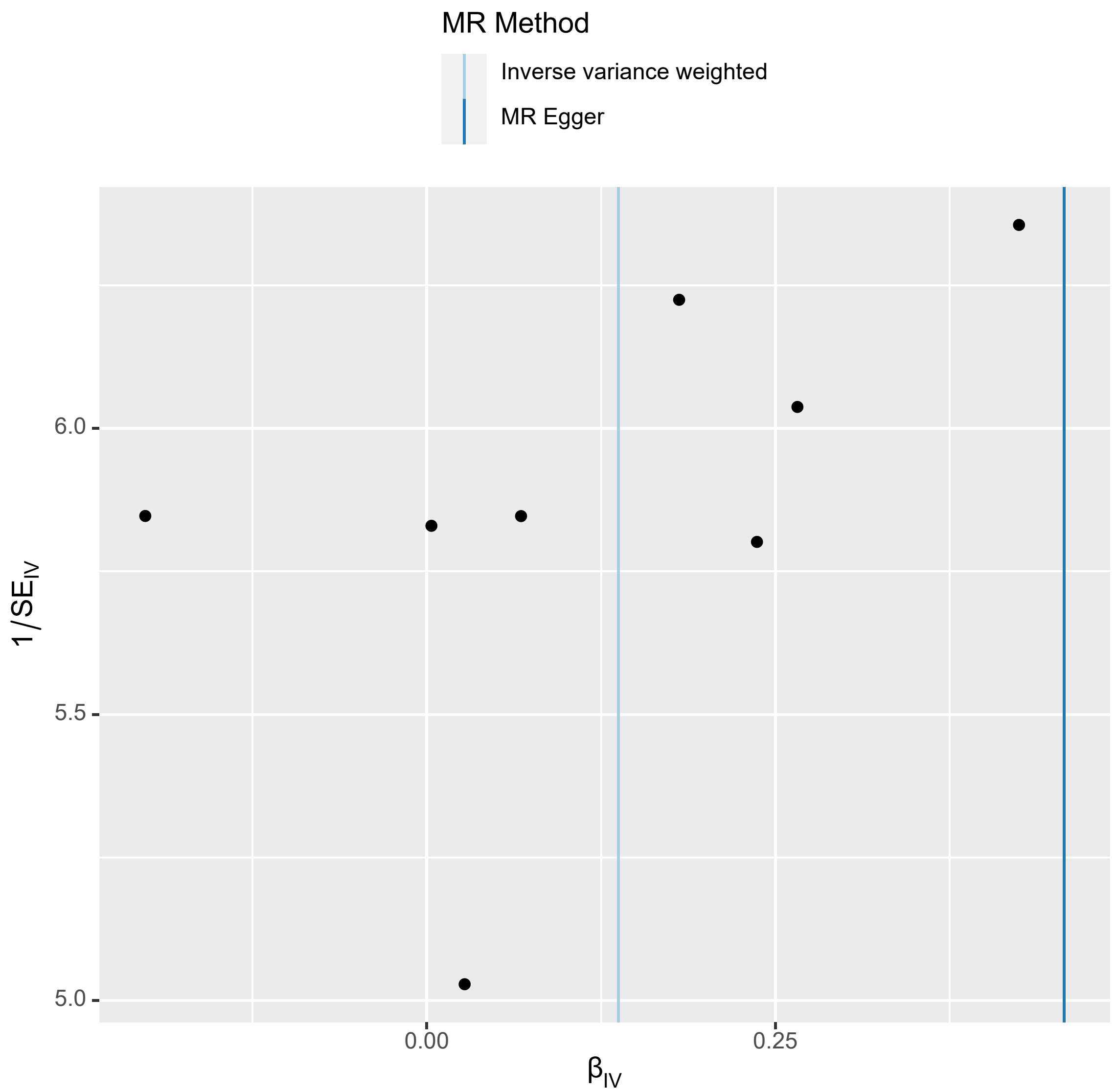

Figure S8. The funnel plot of family Porphyromonadaceae on prostate cancer in the PRACTICAL consortium

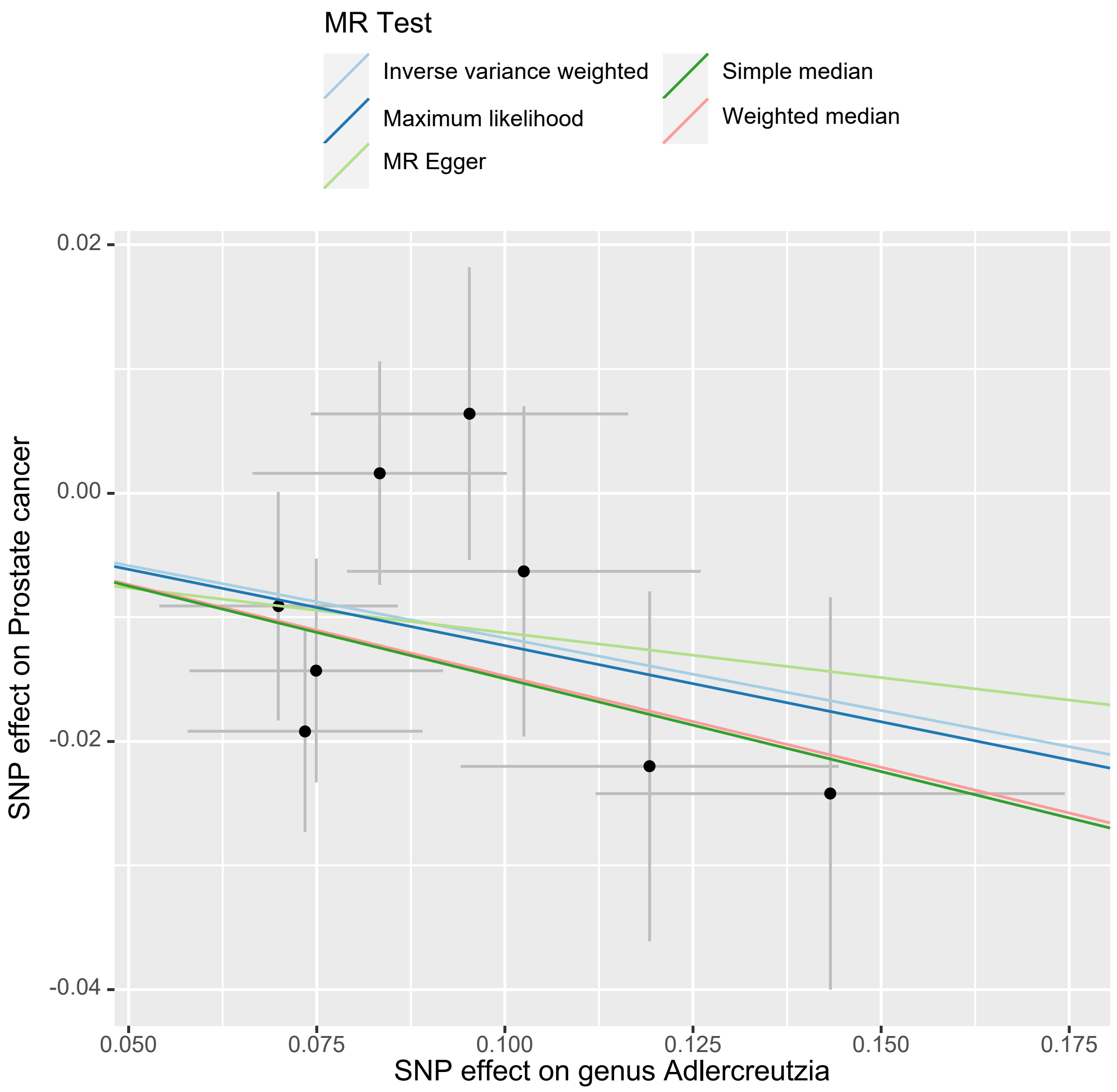

Figure S9. The scatter plot of genus Adlercreutzia on prostate cancer in the PRACTICAL consortium

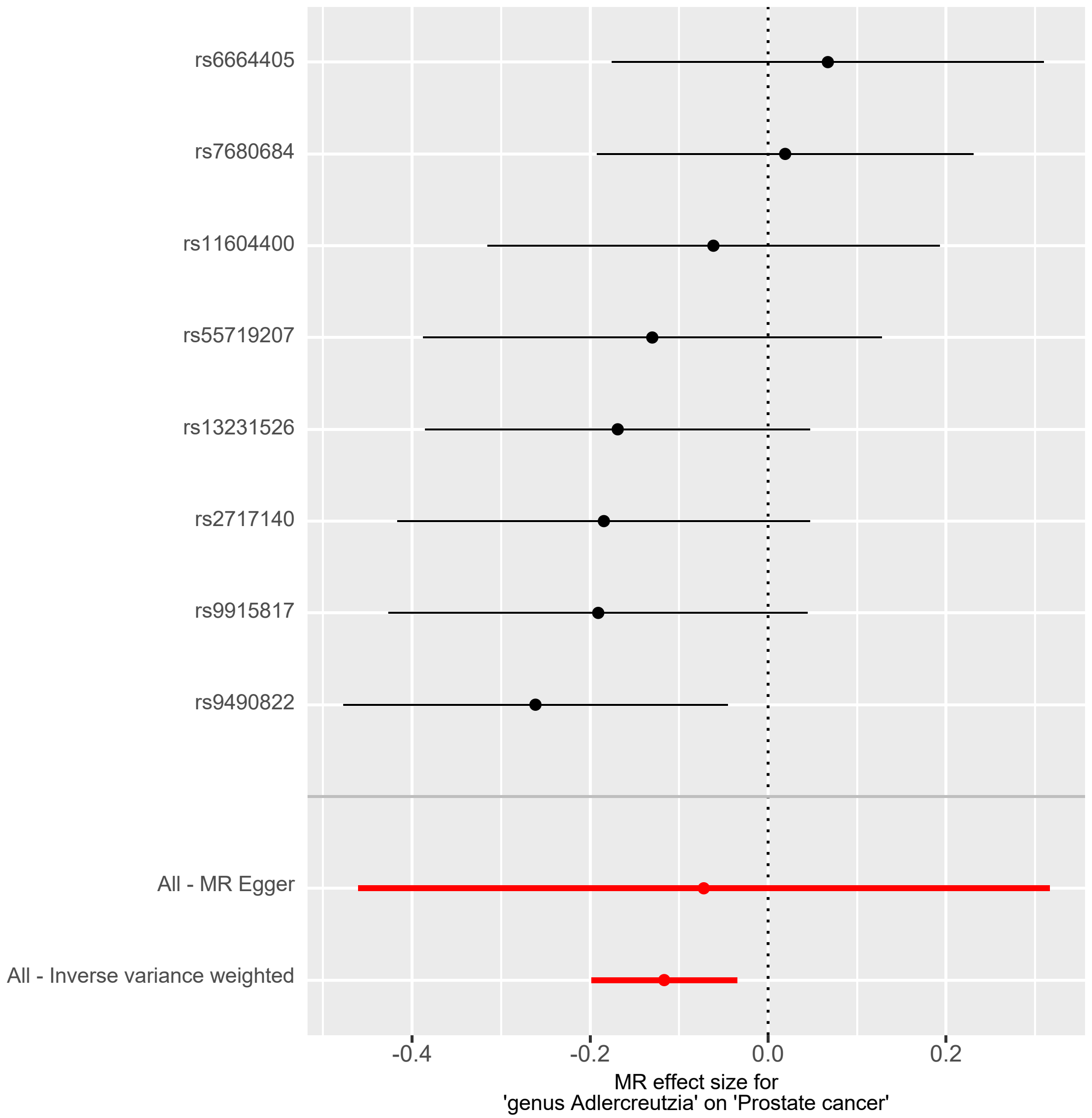

Figure S10. The forest plot of genus Adlercreutzia on prostate cancer in the PRACTICAL consortium

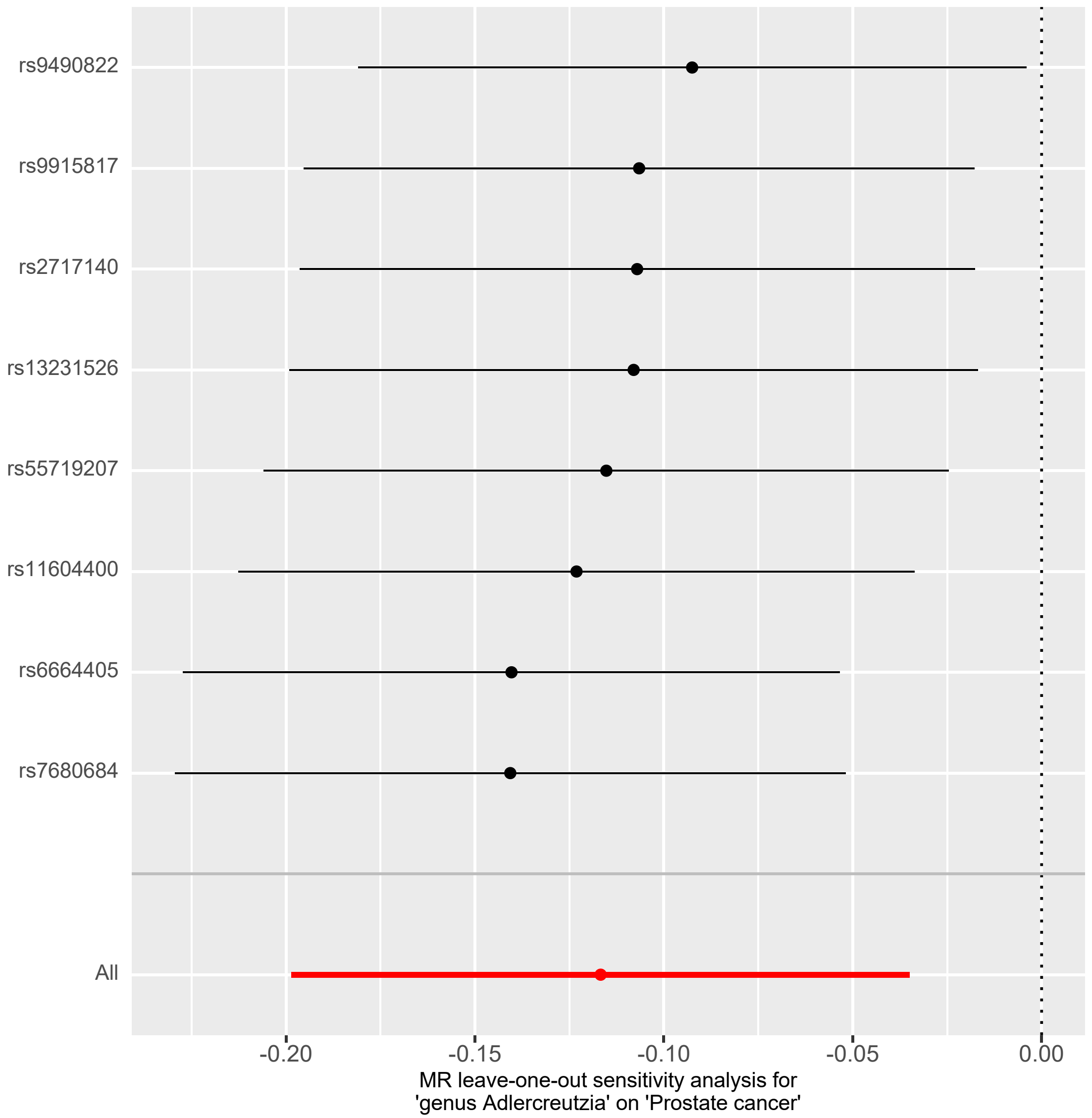

Figure S11. The leave-one-out test of genus Adlercreutzia on prostate cancer in the PRACTICAL consortium

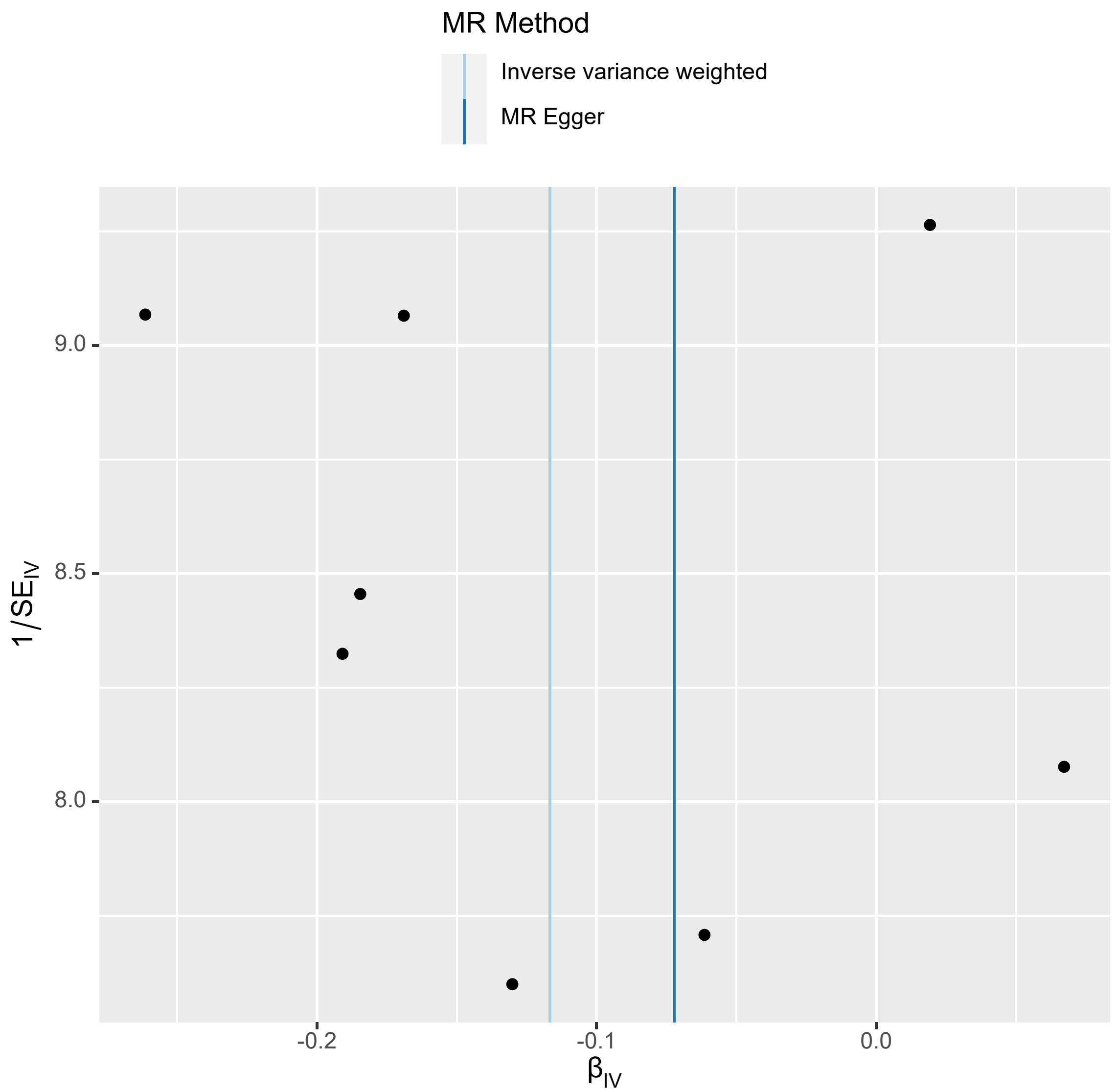

Figure S12. The funnel plot of genus Adlercreutzia on prostate cancer in the PRACTICAL consortium

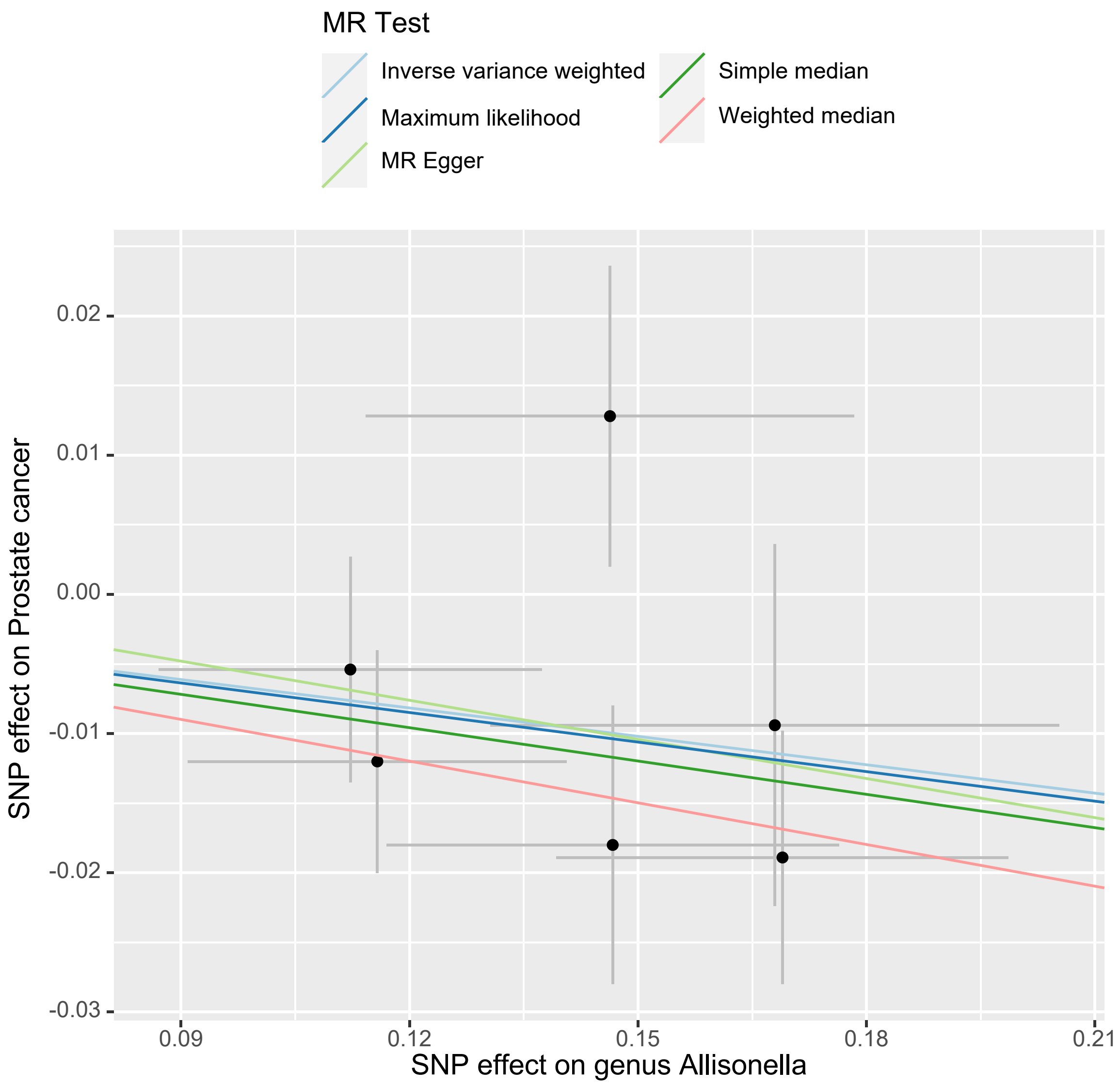

Figure S13. The scatter plot of genus Allisonella on prostate cancer in the PRACTICAL consortium

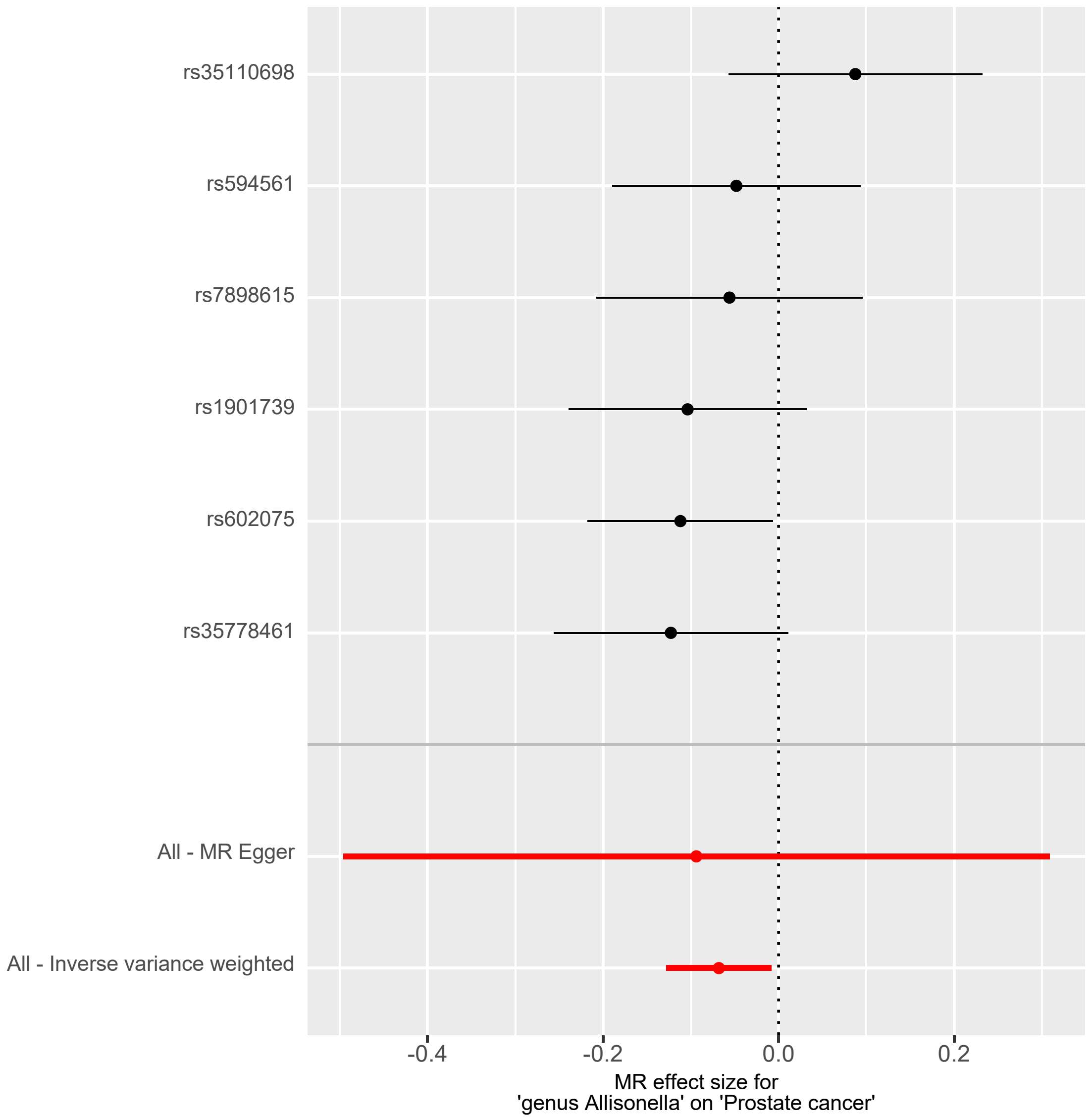

Figure S14. The forest plot of genus Allisonella on prostate cancer in the PRACTICAL consortium

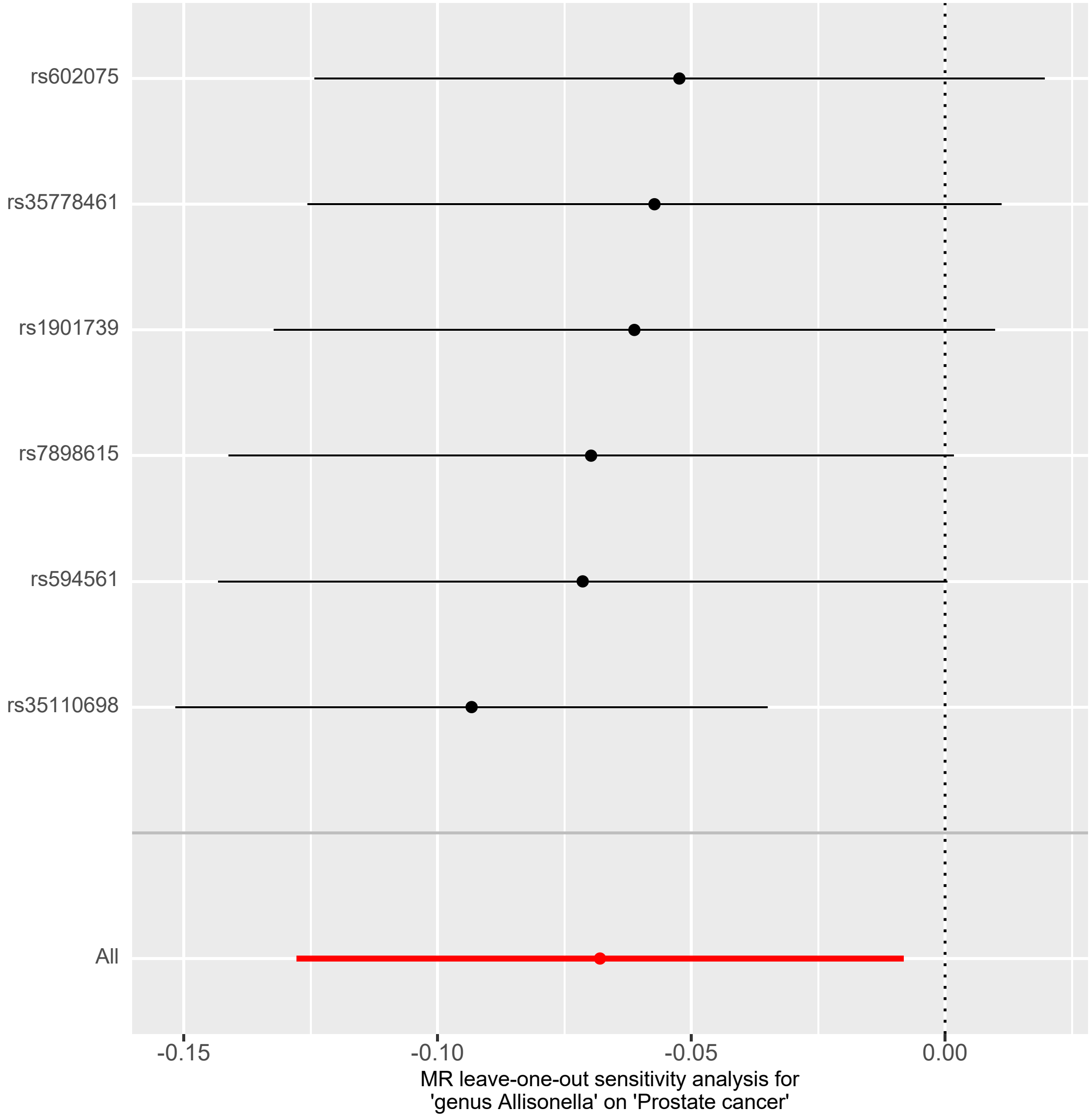

Figure S15. The leave-one-out test of genus Allisonella on prostate cancer in the PRACTICAL consortium

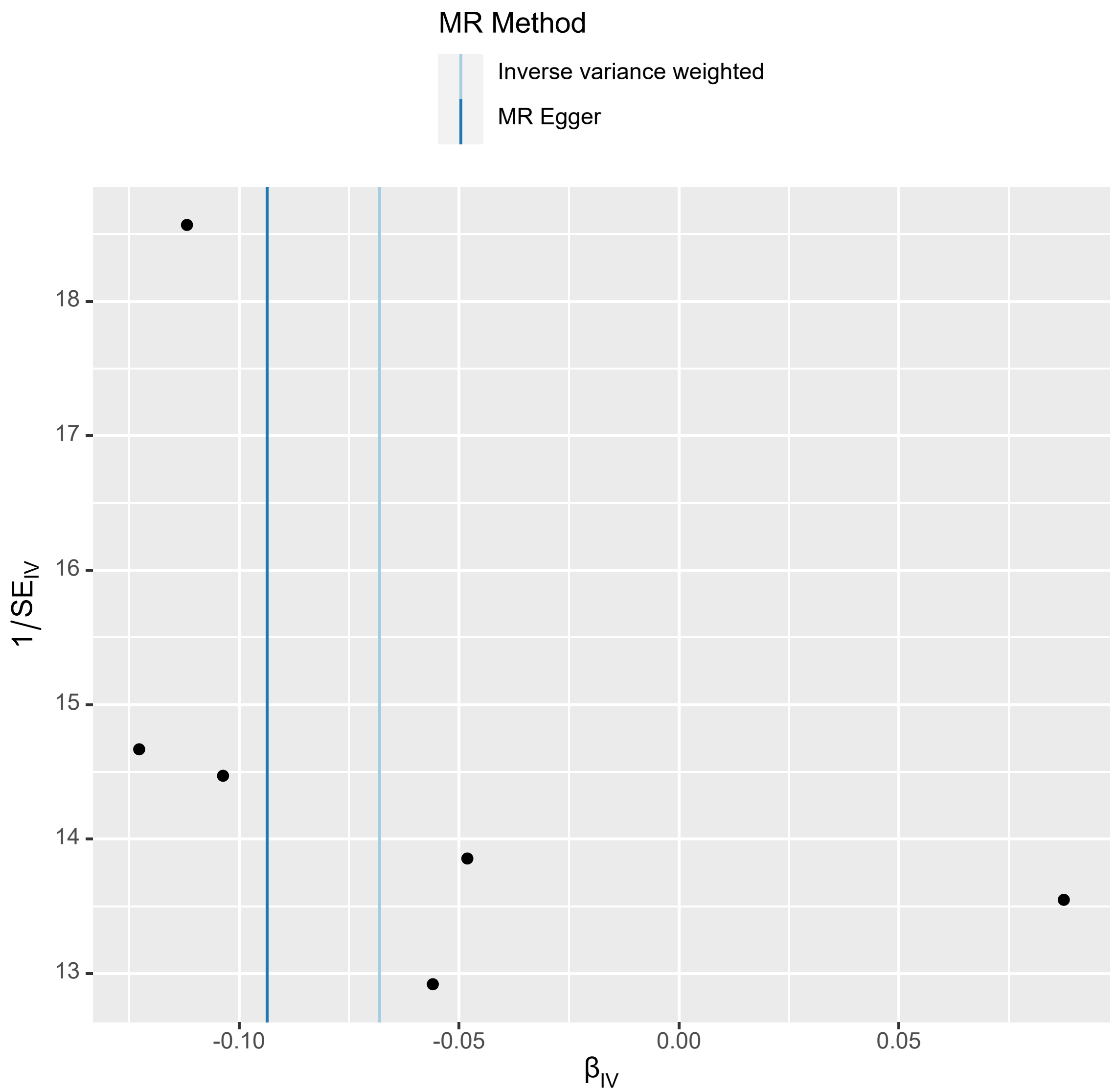

Figure S16. The funnel plot of genus Adlercreutzia on prostate cancer in the PRACTICAL consortium

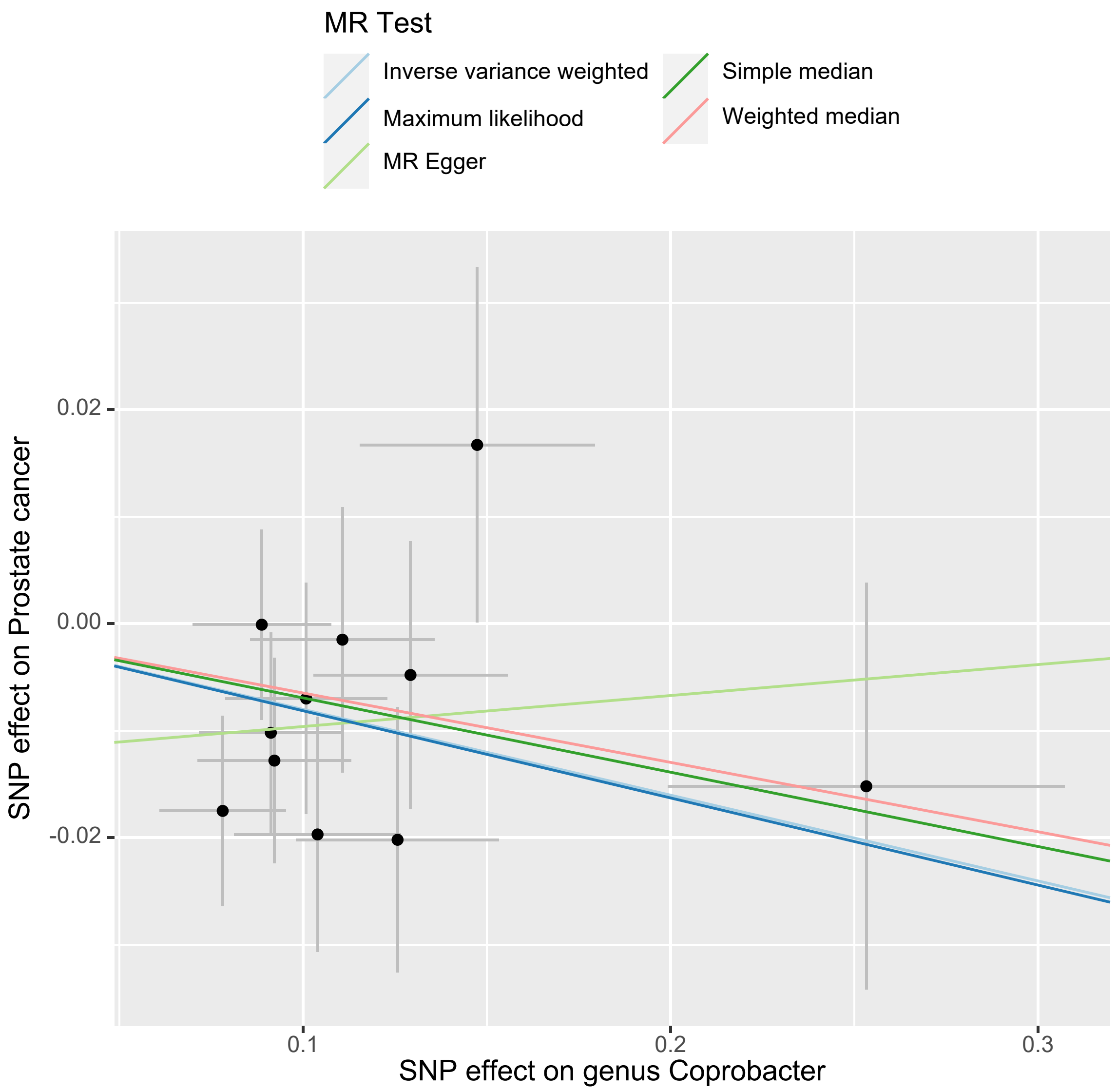

Figure S17. The scatter plot of genus Coprobacter on prostate cancer in the PRACTICAL consortium

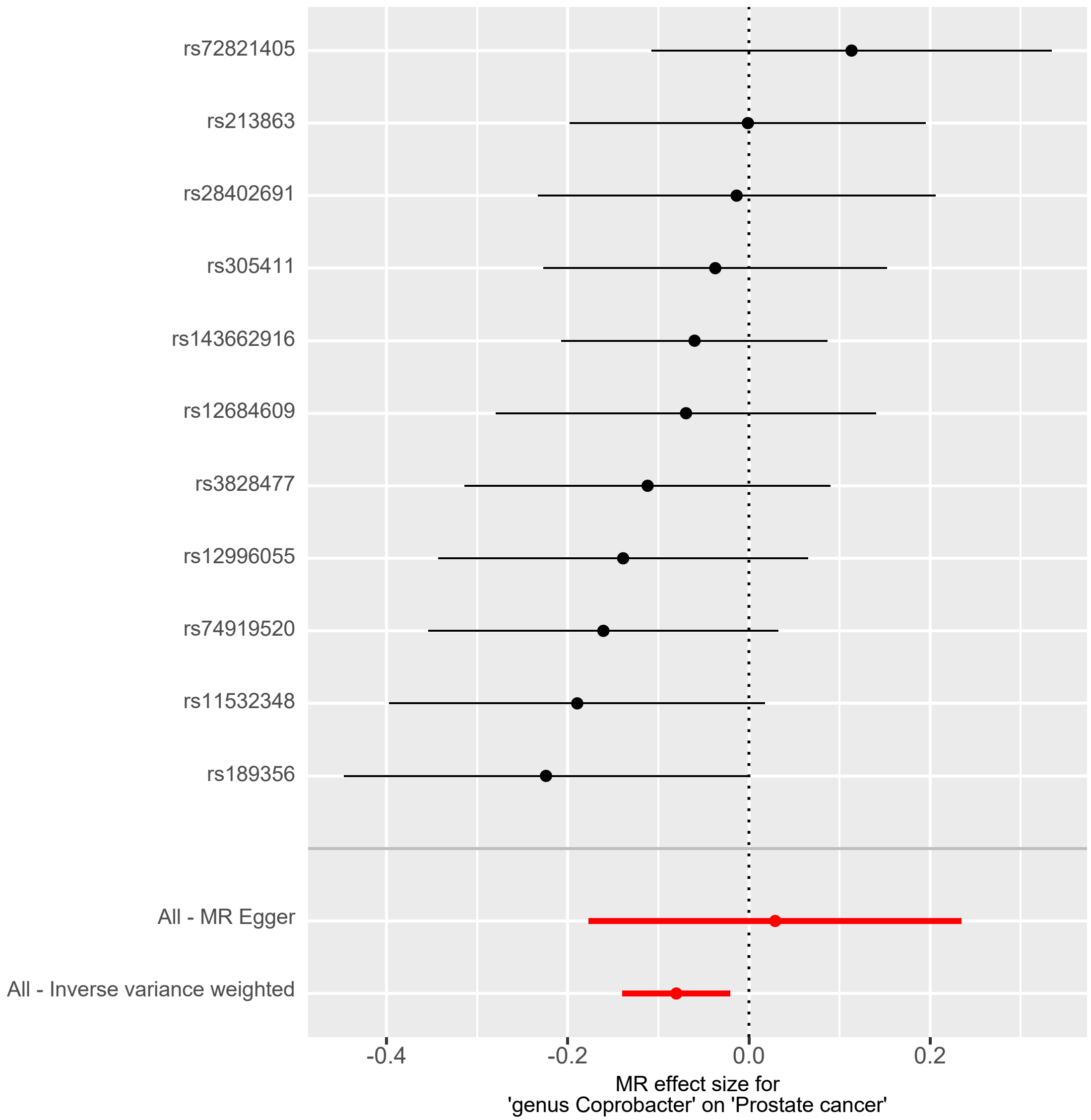

Figure S18. The forest plot of genus Coprobacter on prostate cancer in the PRACTICAL consortium

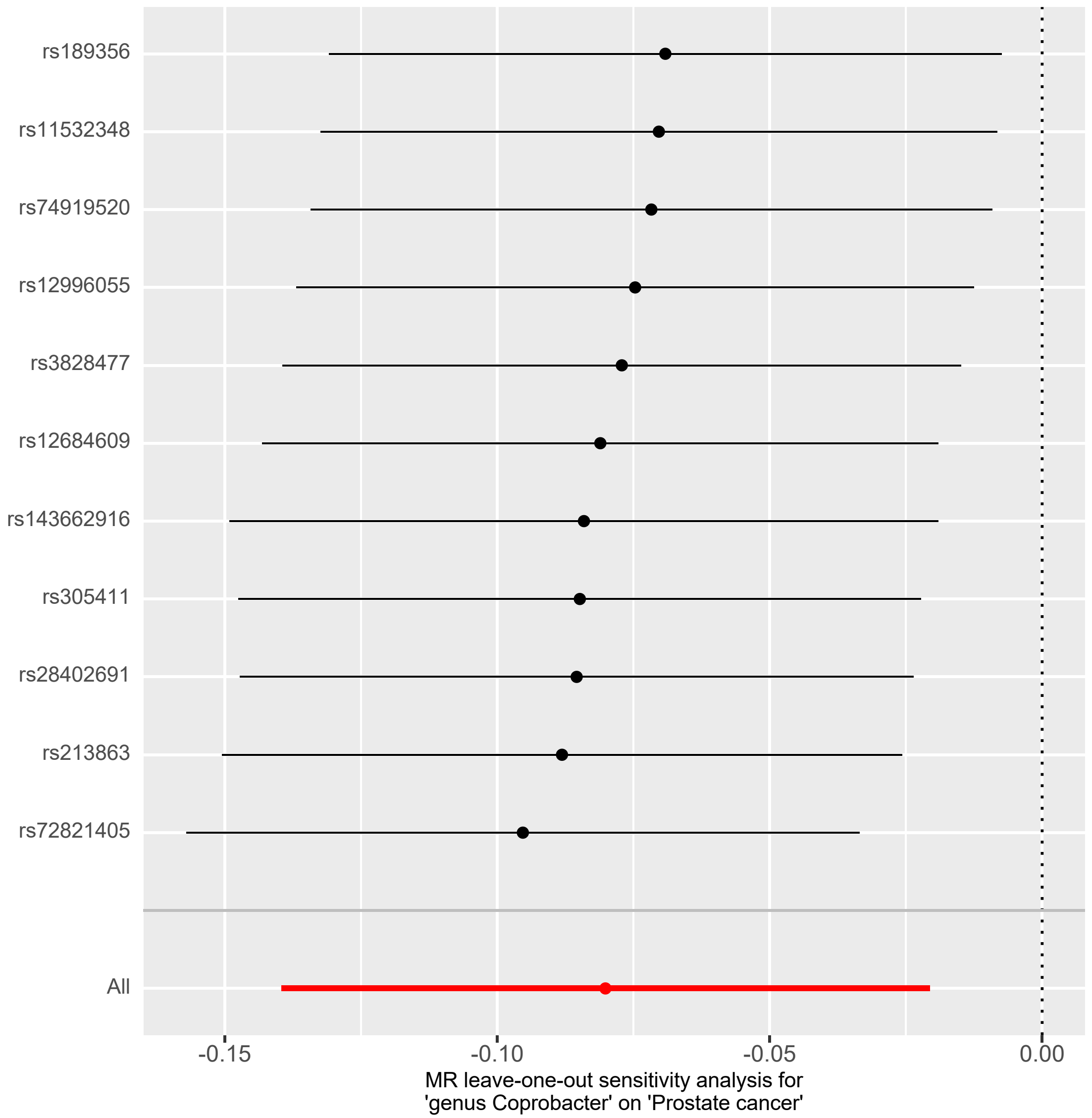

Figure S19. The leave-one-out test of genus Coprobacter on prostate cancer in the PRACTICAL consortium

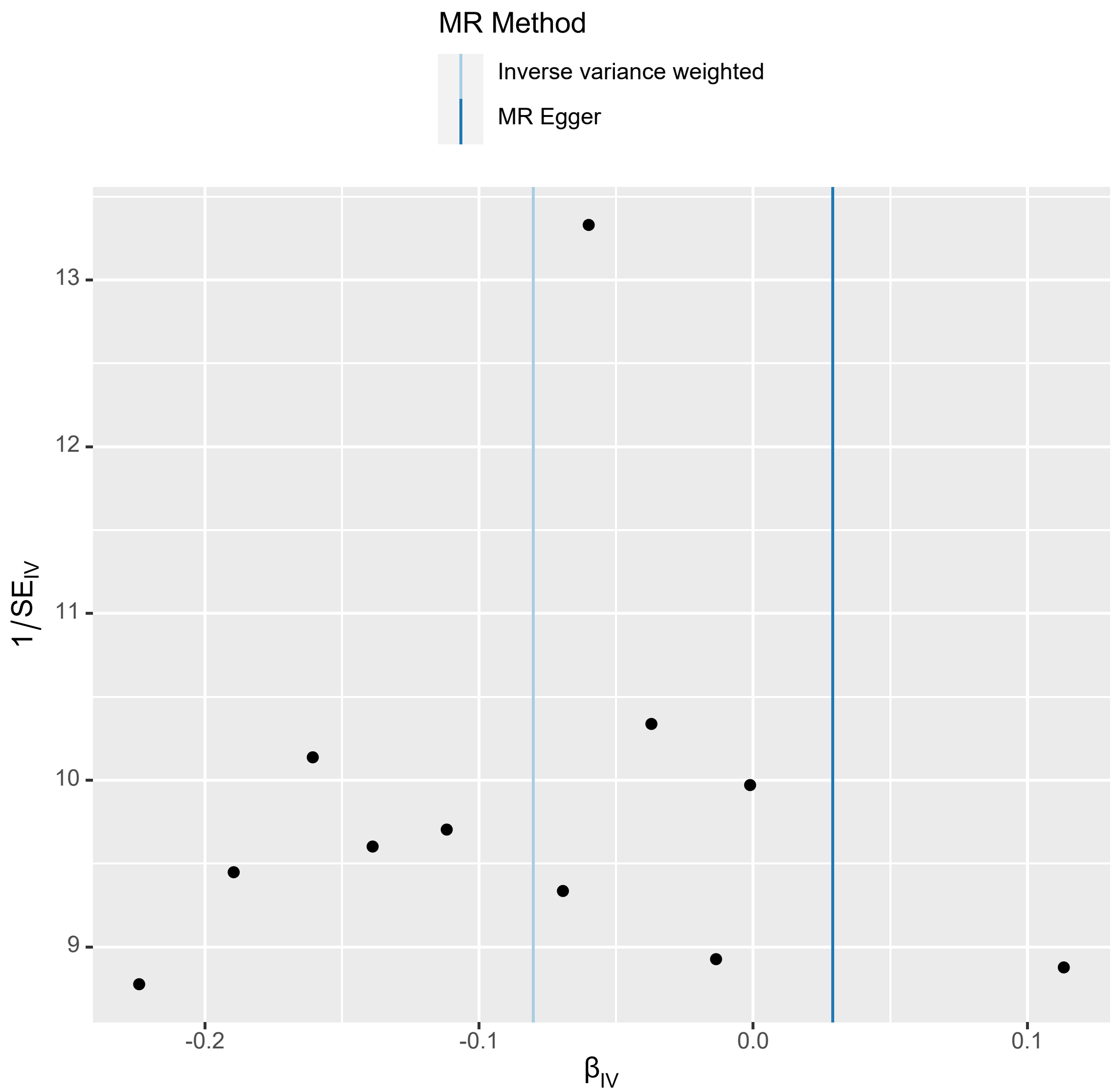

Figure S20. The funnel plot of genus Coprobacter on prostate cancer in the PRACTICAL consortium

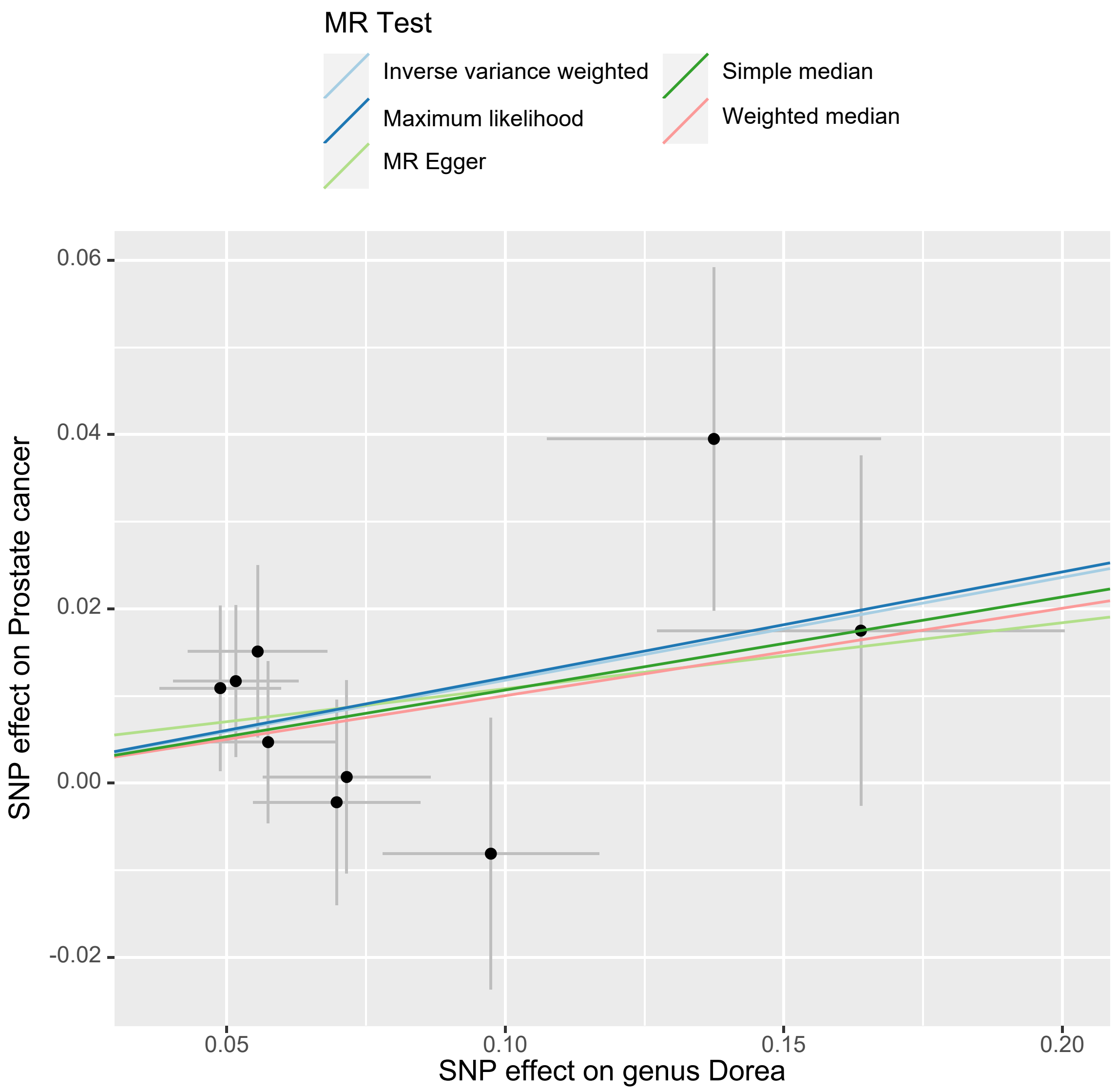

Figure S21. The scatter plot of genus Dorea on prostate cancer in the PRACTICAL consortium

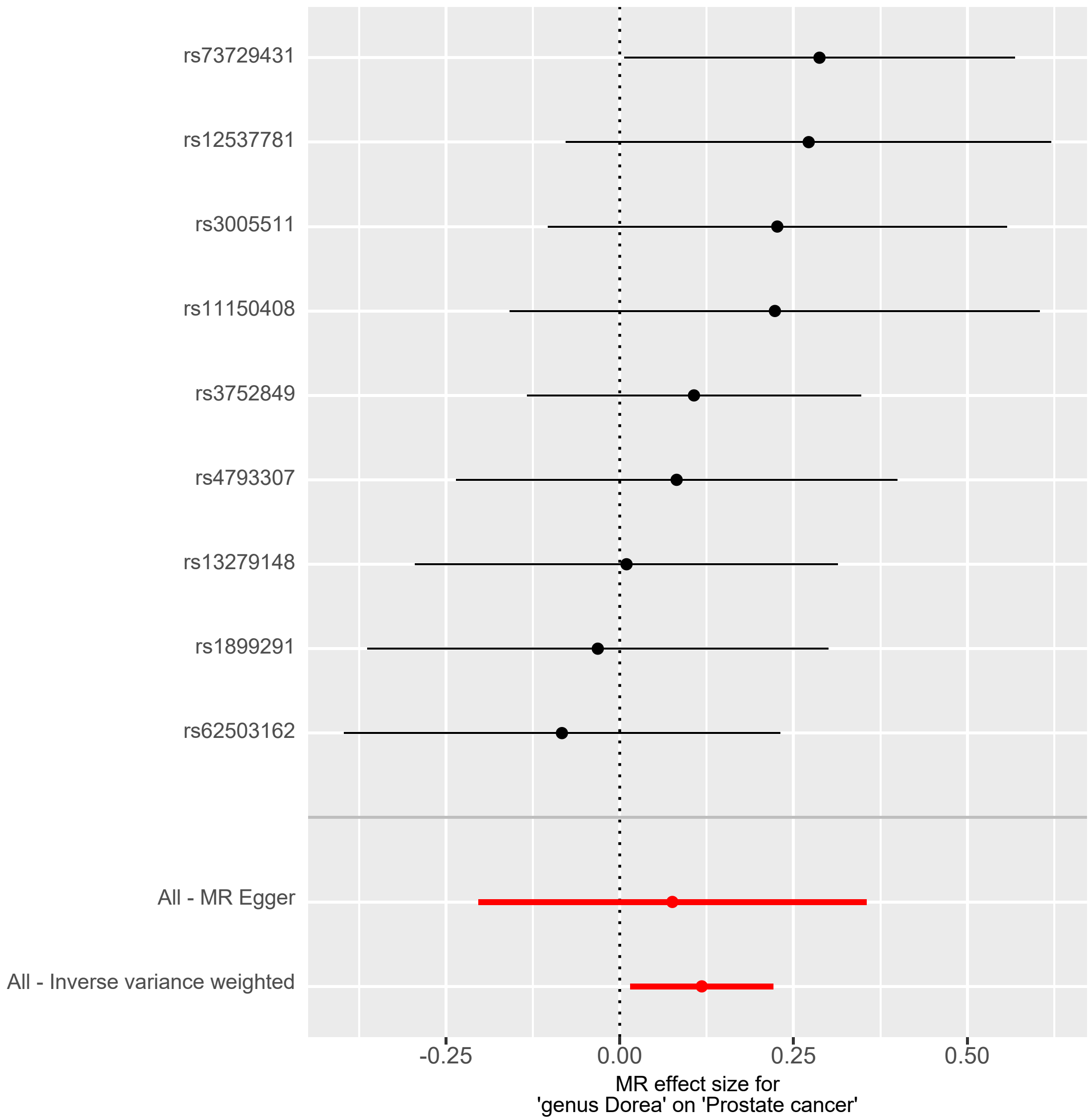

Figure S22. The forest plot of genus Dorea on prostate cancer in the PRACTICAL consortium

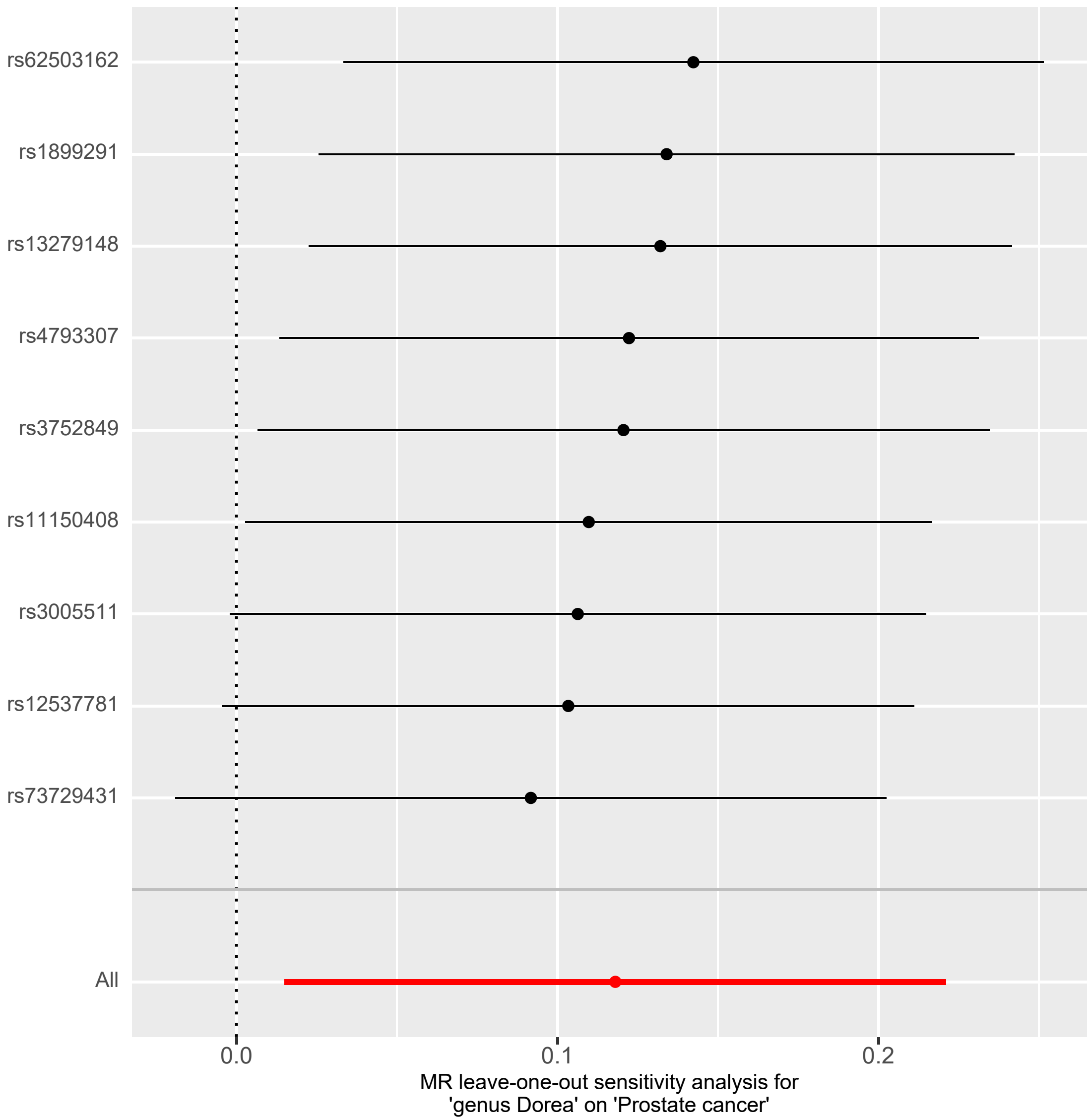

Figure S23. The leave-one-out test of genus Dorea on prostate cancer in the PRACTICAL consortium

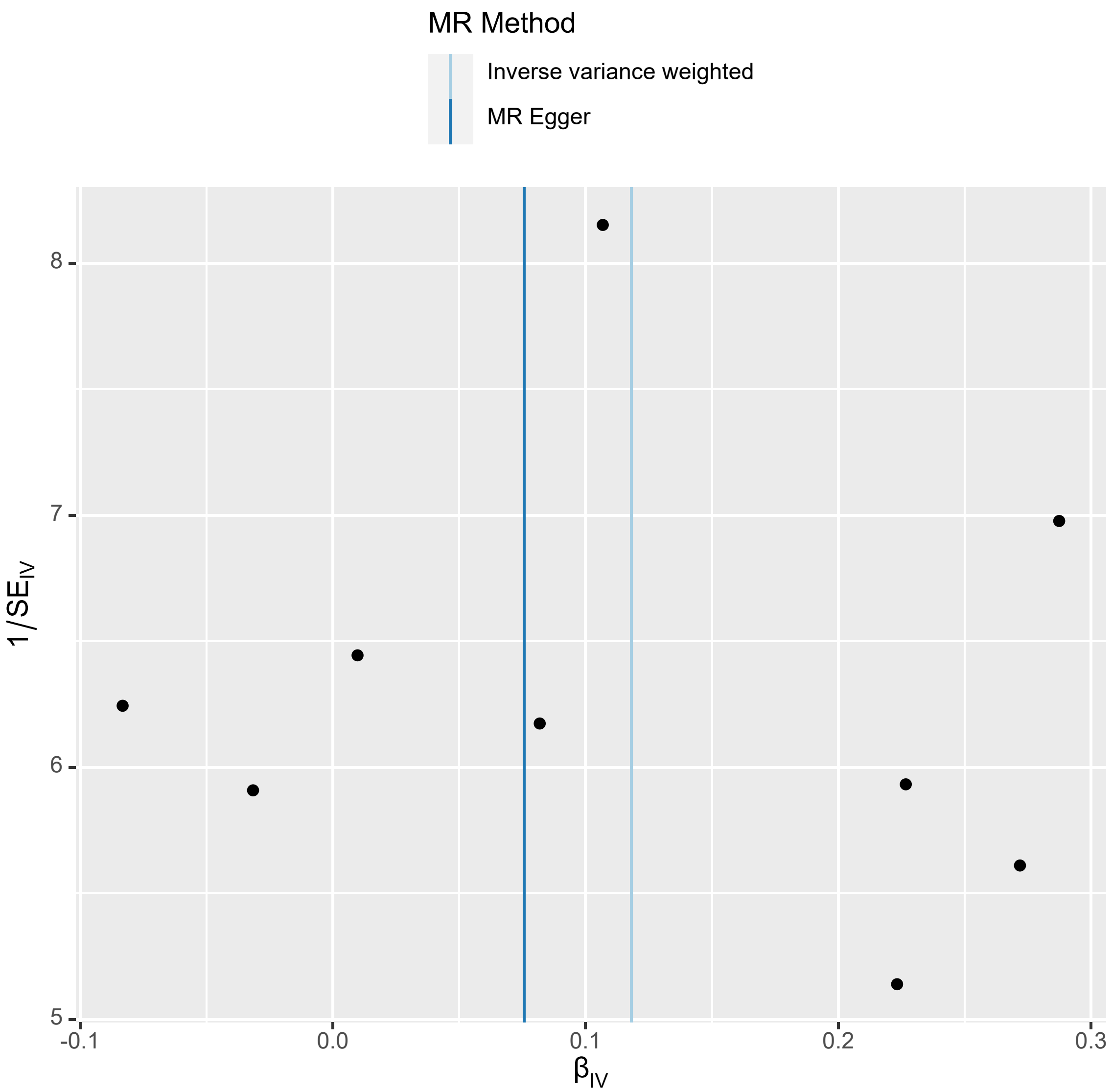

Figure S24. The funnel plot of genus Dorea on prostate cancer in the PRACTICAL consortium

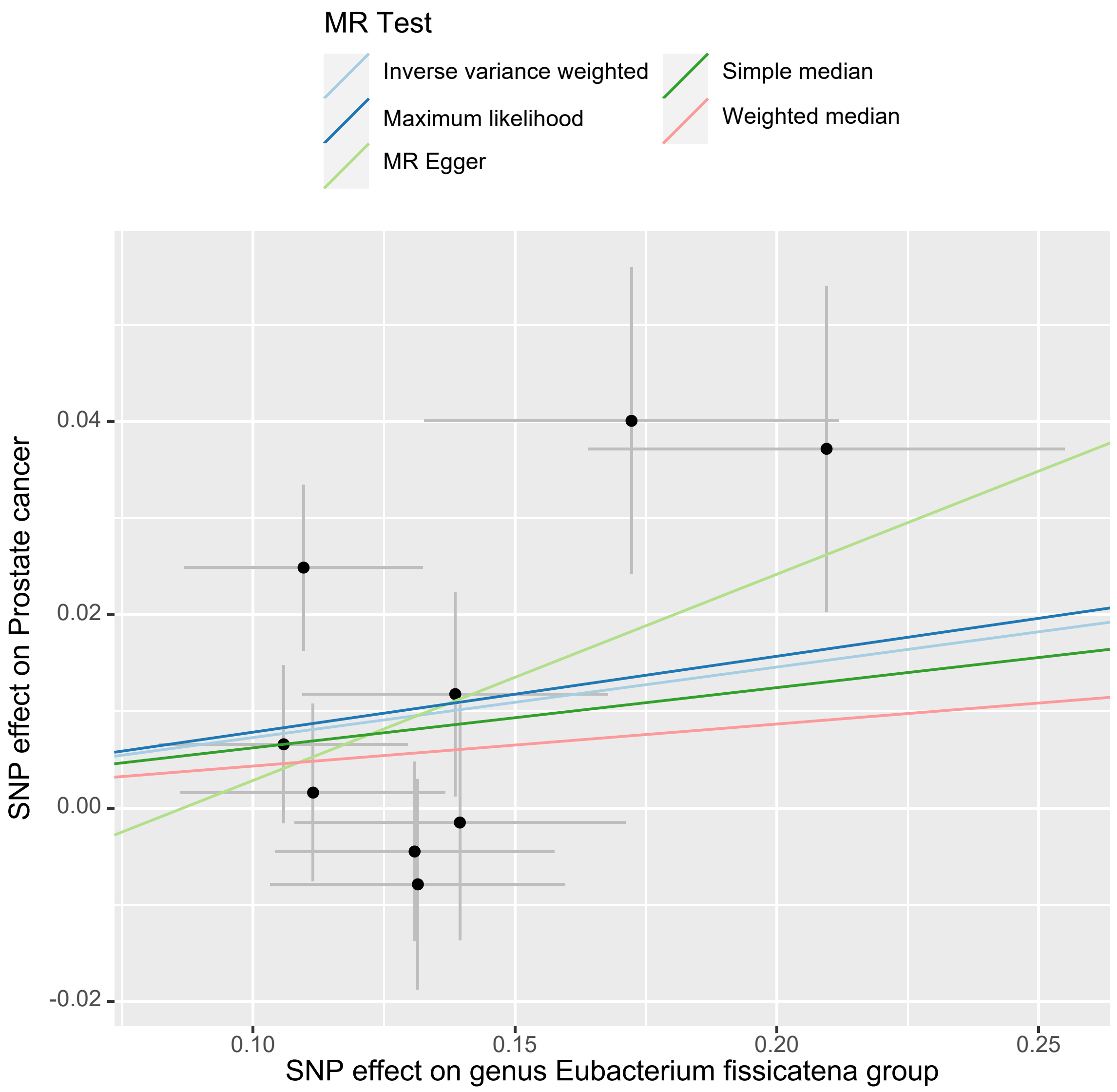

Figure S25. The scatter plot of genus Eubacterium fissicatena group on prostate cancer in the PRACTICAL consortium

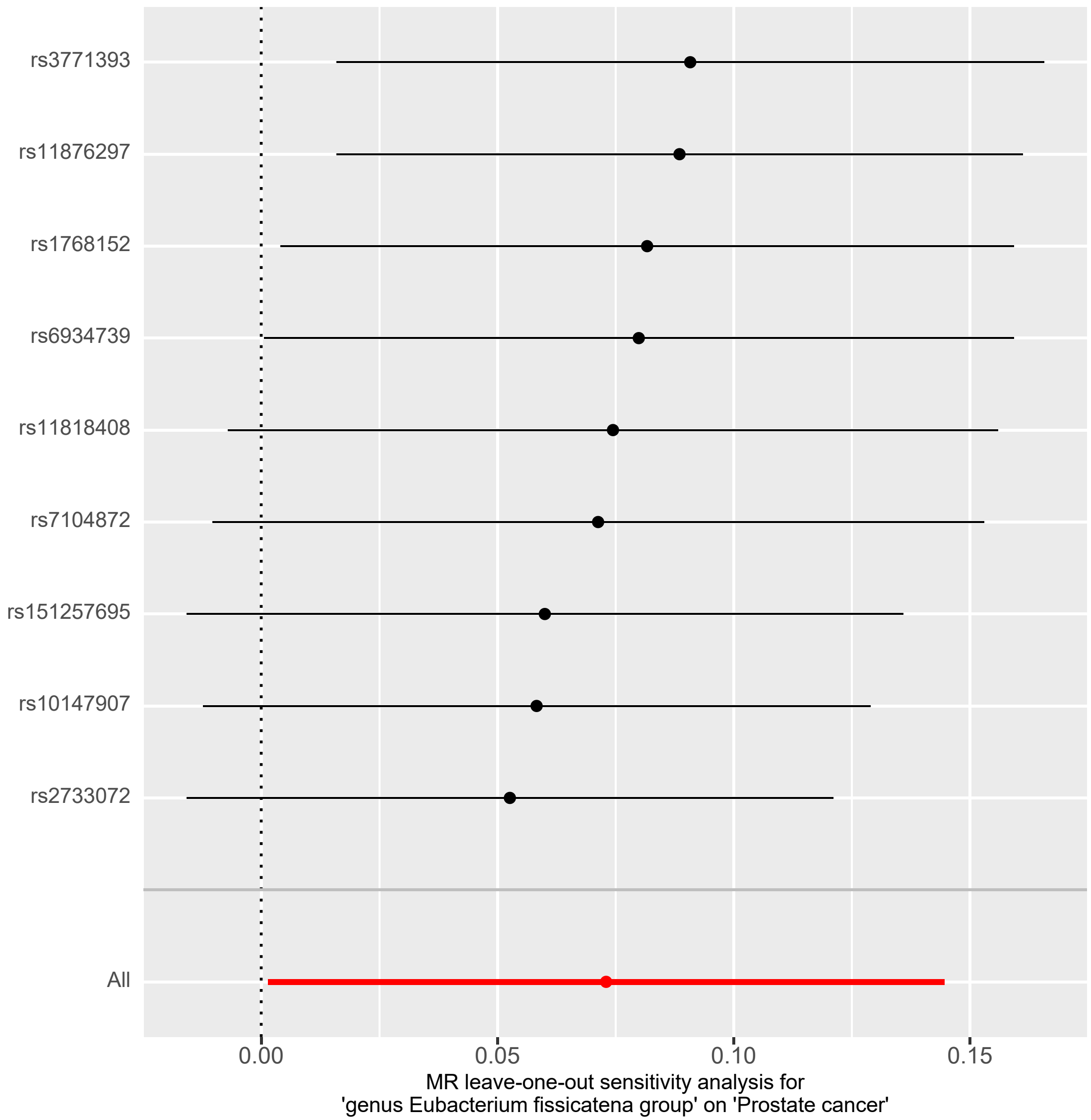

Figure S26. The forest plot of genus Eubacterium fissicatena group on prostate cancer in the PRACTICAL consortium

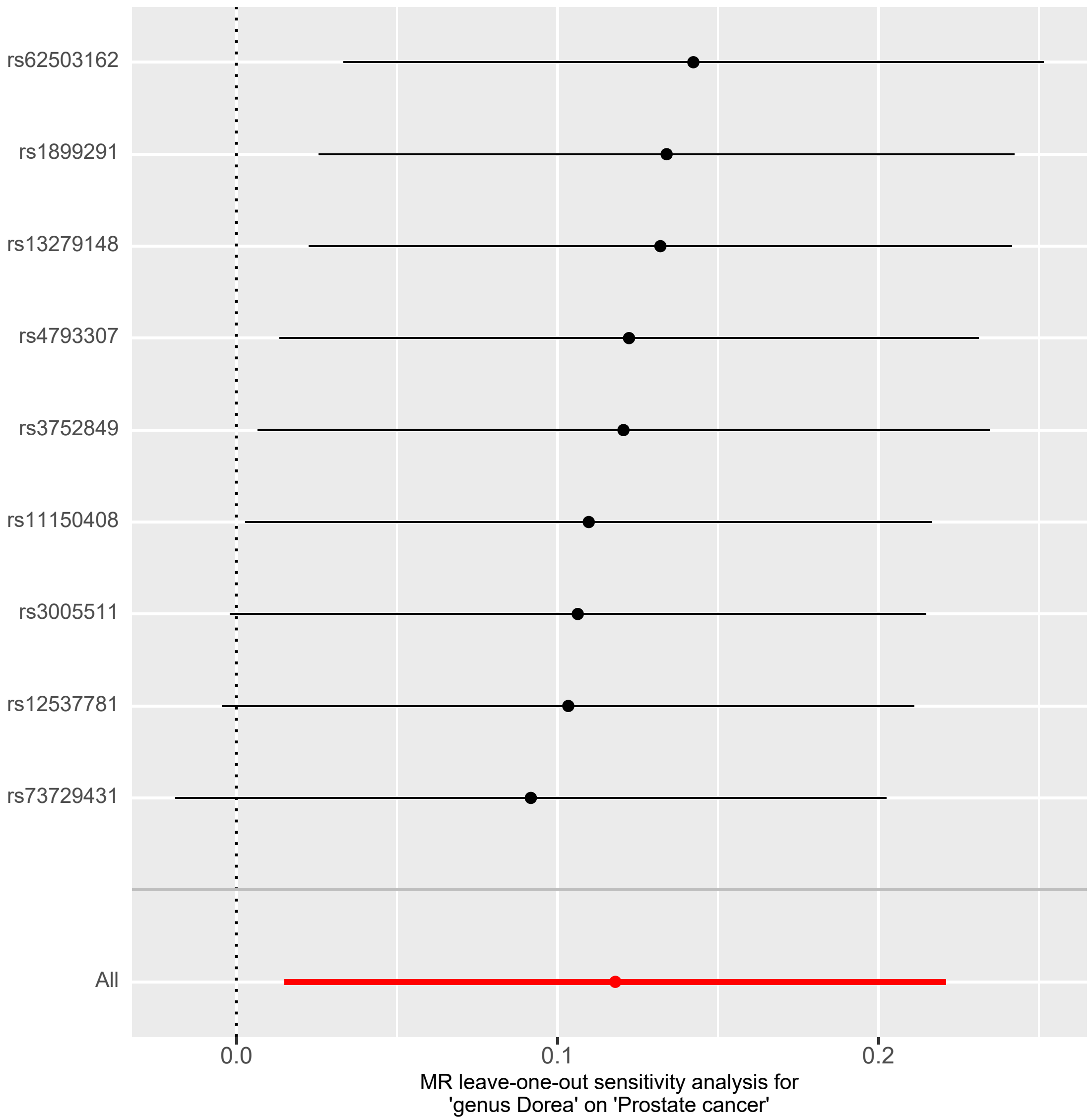

Figure S27. The leave-one-out test of genus Eubacterium fissicatena group on prostate cancer in the PRACTICAL consortium

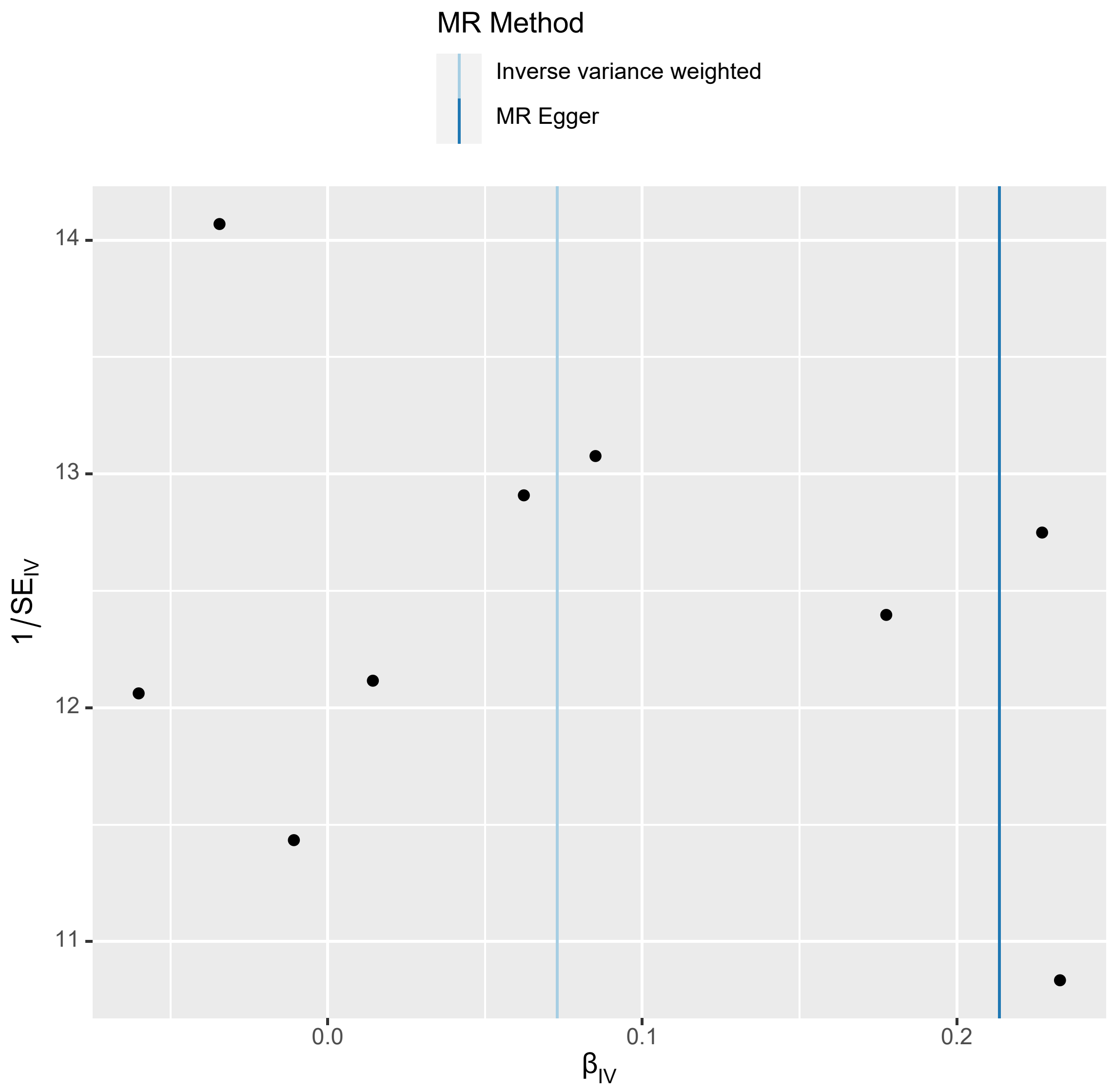

Figure S28. The funnel plot of genus Eubacterium fissicatena group on prostate cancer in the PRACTICAL consortium

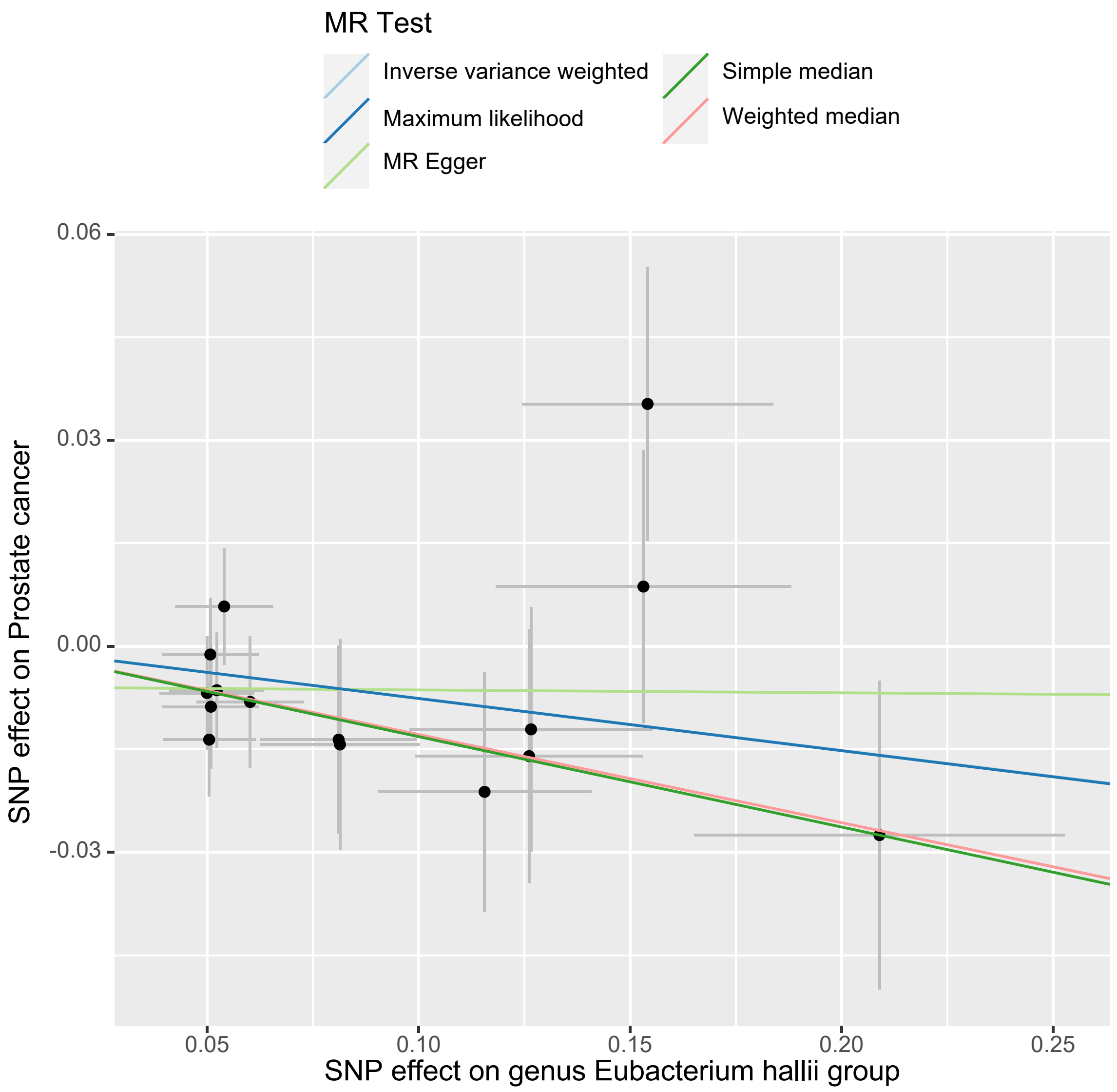

Figure S29. The scatter plot of genus Eubacterium hallii group on prostate cancer in the PRACTICAL consortium

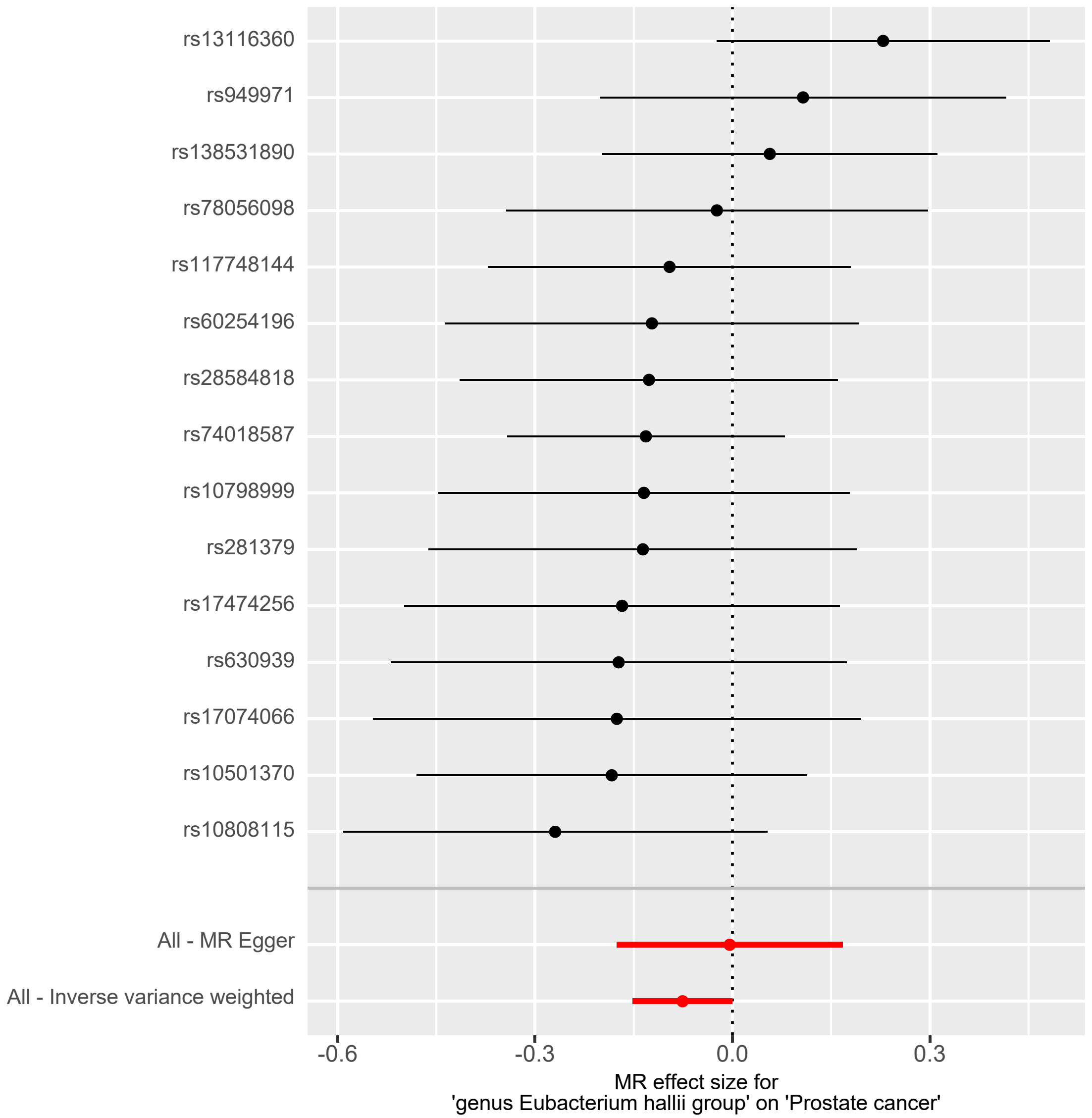

Figure S30. The forest plot of genus Eubacterium hallii group on prostate cancer in the PRACTICAL consortium

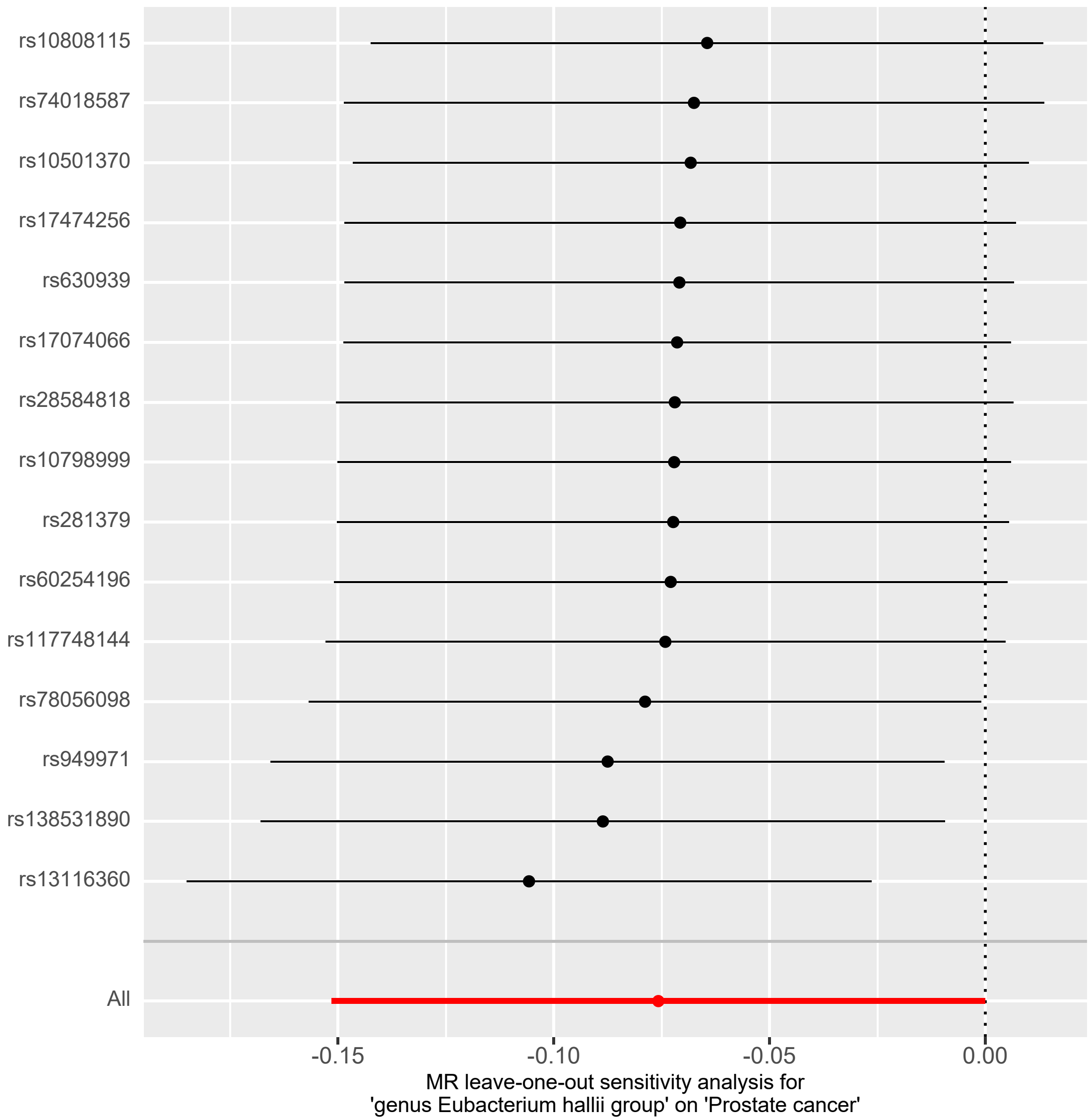

Figure S31. The leave-one-out test of genus Eubacterium hallii group on prostate cancer in the PRACTICAL consortium

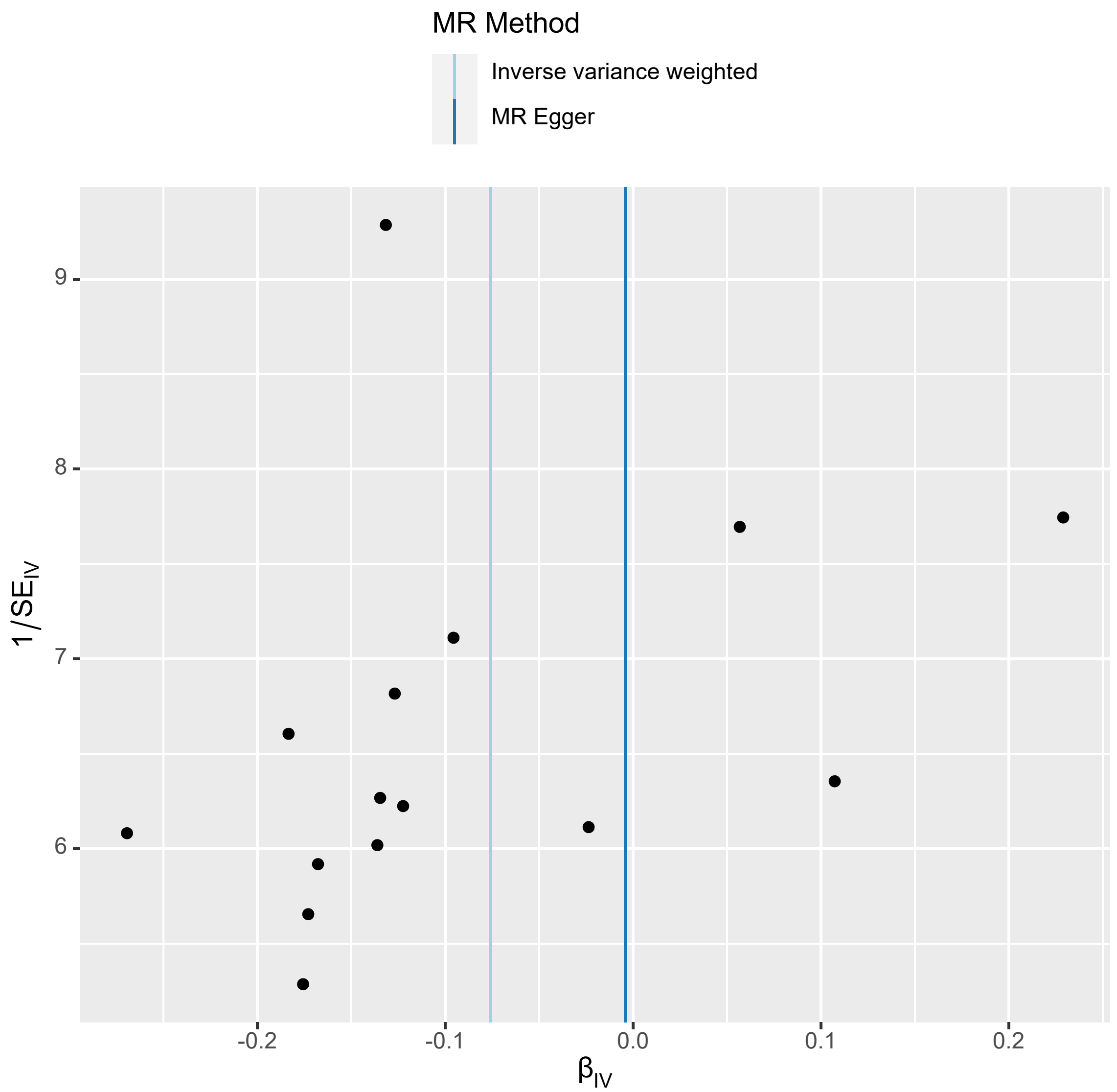

Figure S32. The funnel plot of genus Eubacterium hallii group on prostate cancer in the PRACTICAL consortium

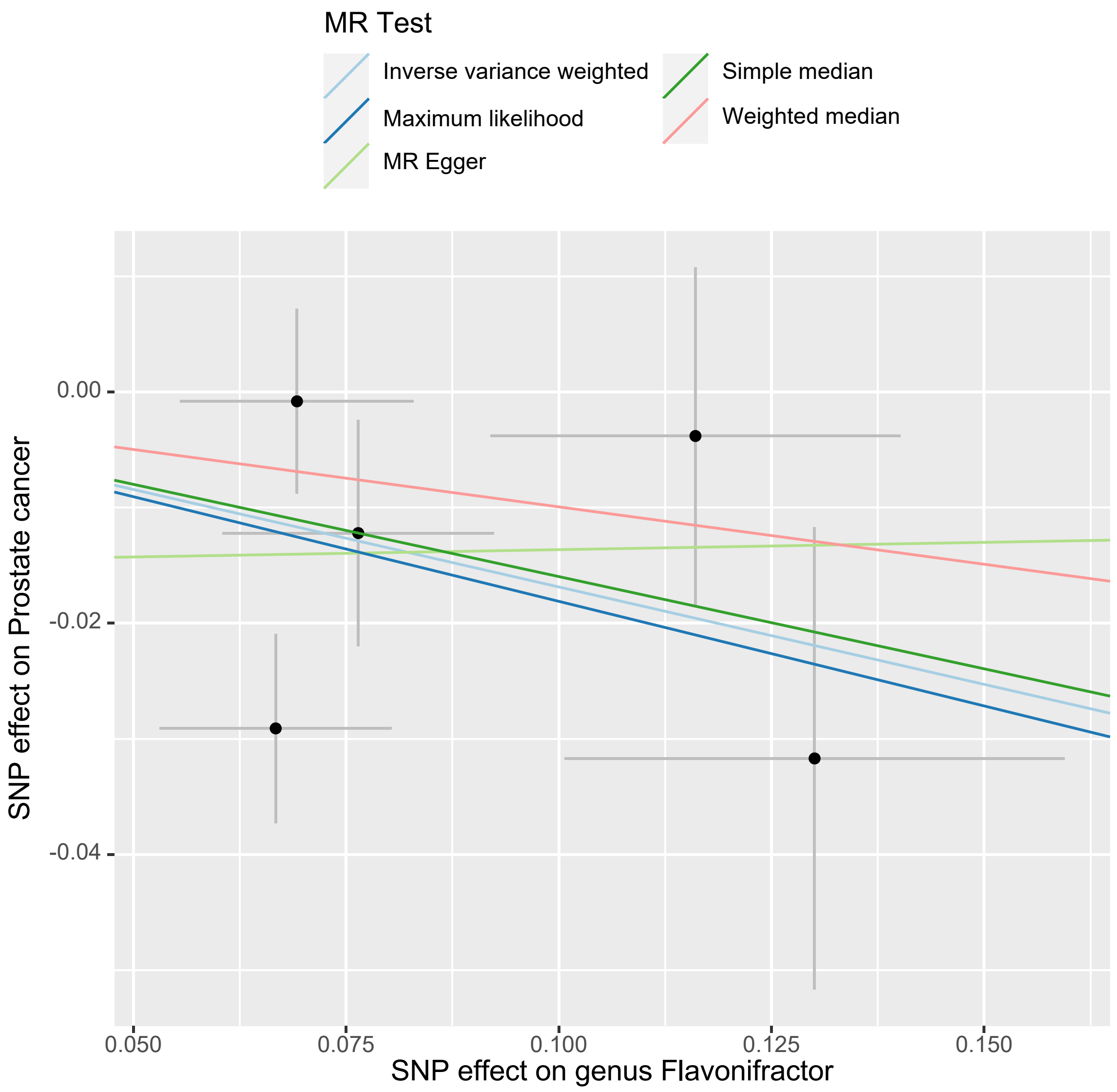

Figure S33. The scatter plot of genus Flavonifractor on prostate cancer in the PRACTICAL consortium

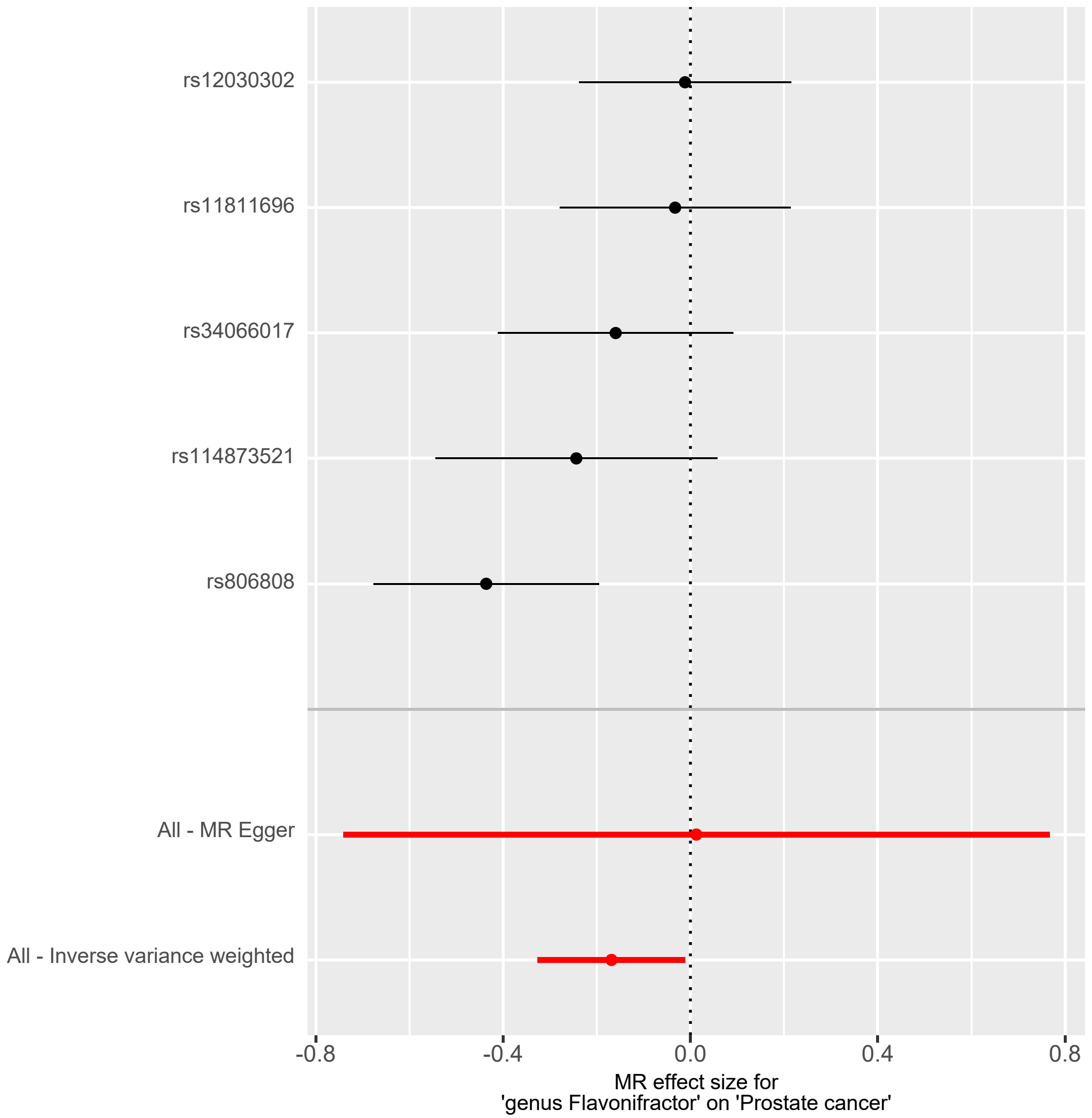

Figure S34. The forest plot of genus Flavonifractor on prostate cancer in the PRACTICAL consortium

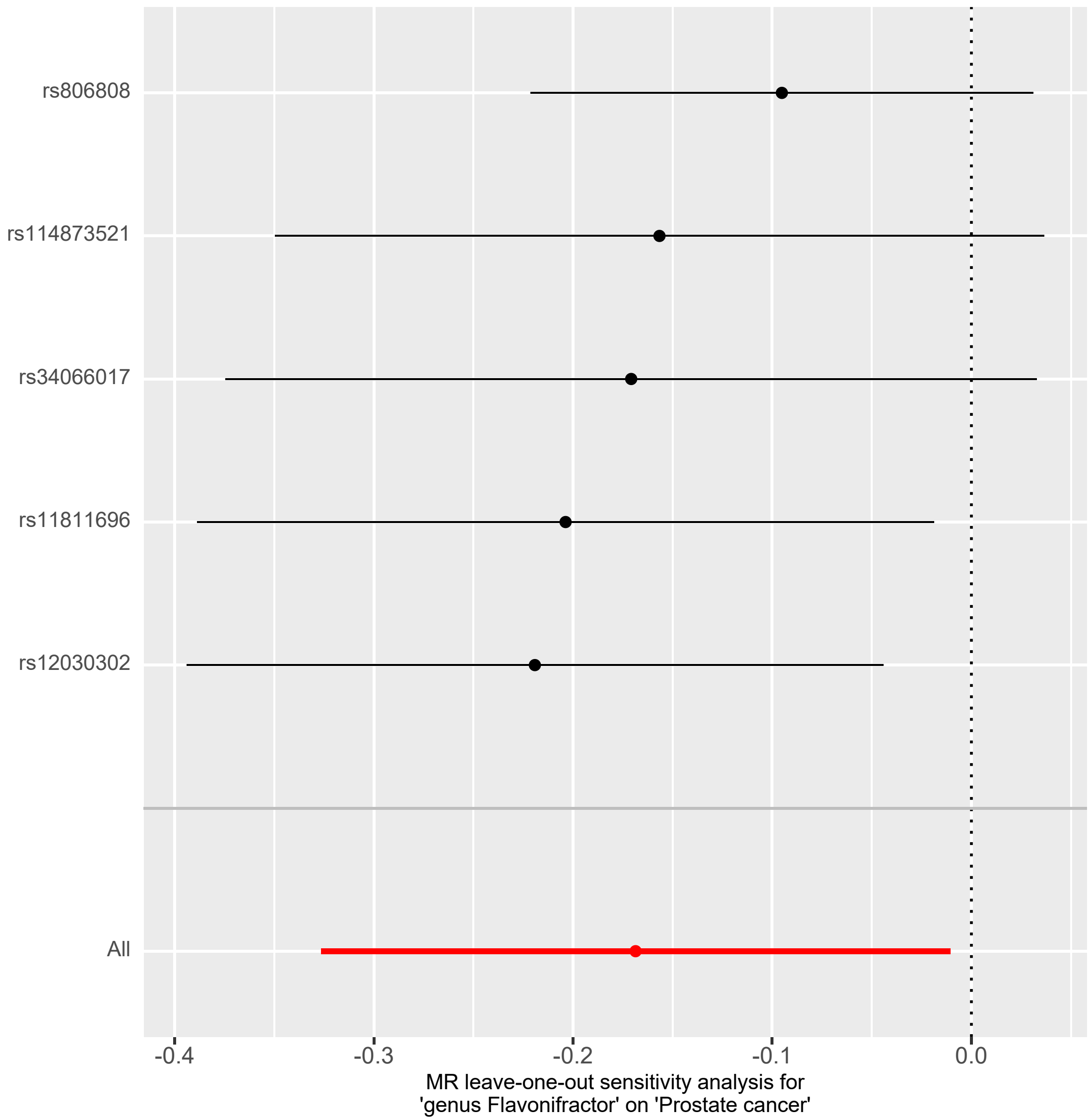

Figure S35. The leave-one-out test of genus Flavonifractor on prostate cancer in the PRACTICAL consortium

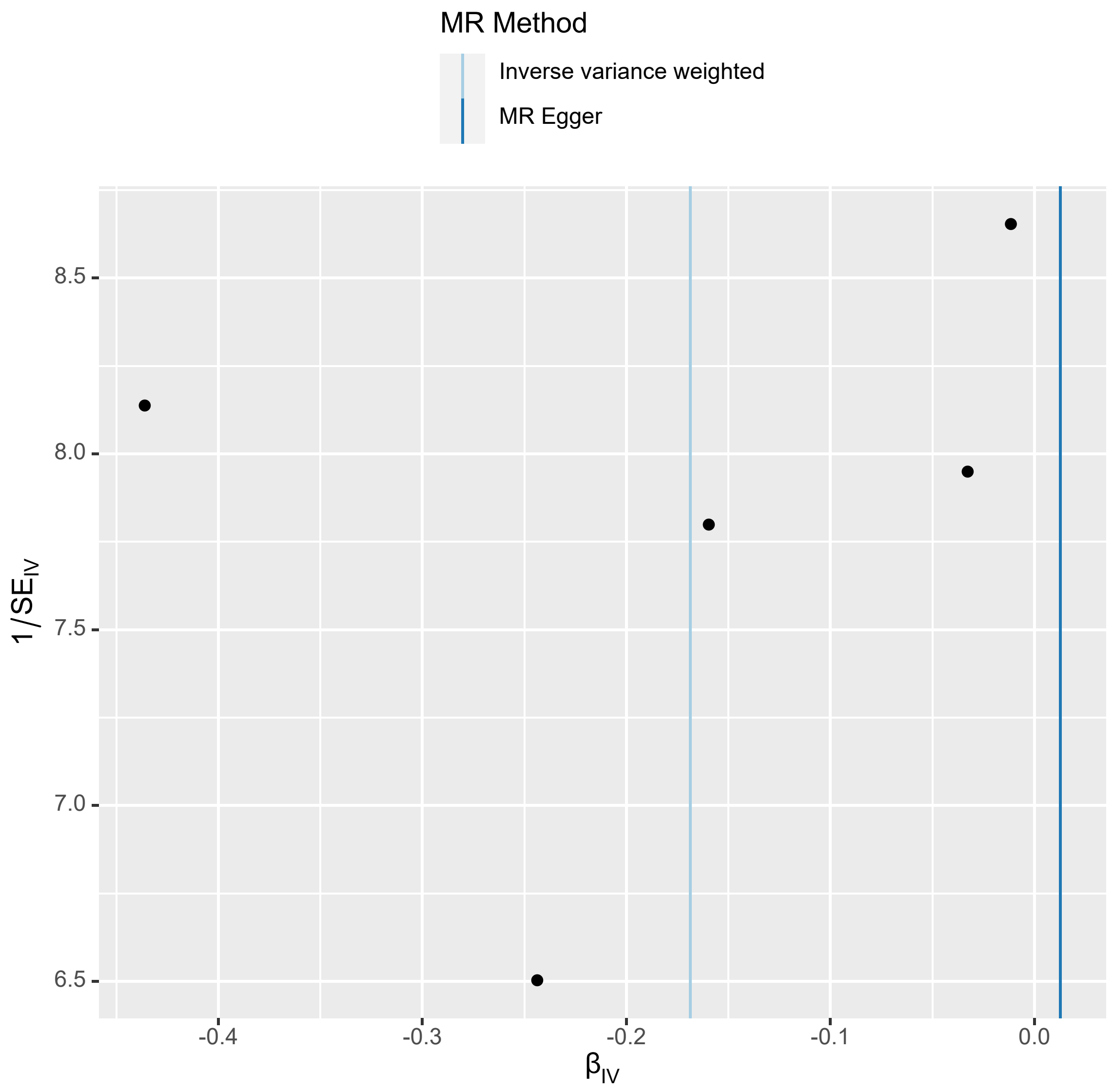

Figure S36. The funnel plot of genus Flavonifractor on prostate cancer in the PRACTICAL consortium

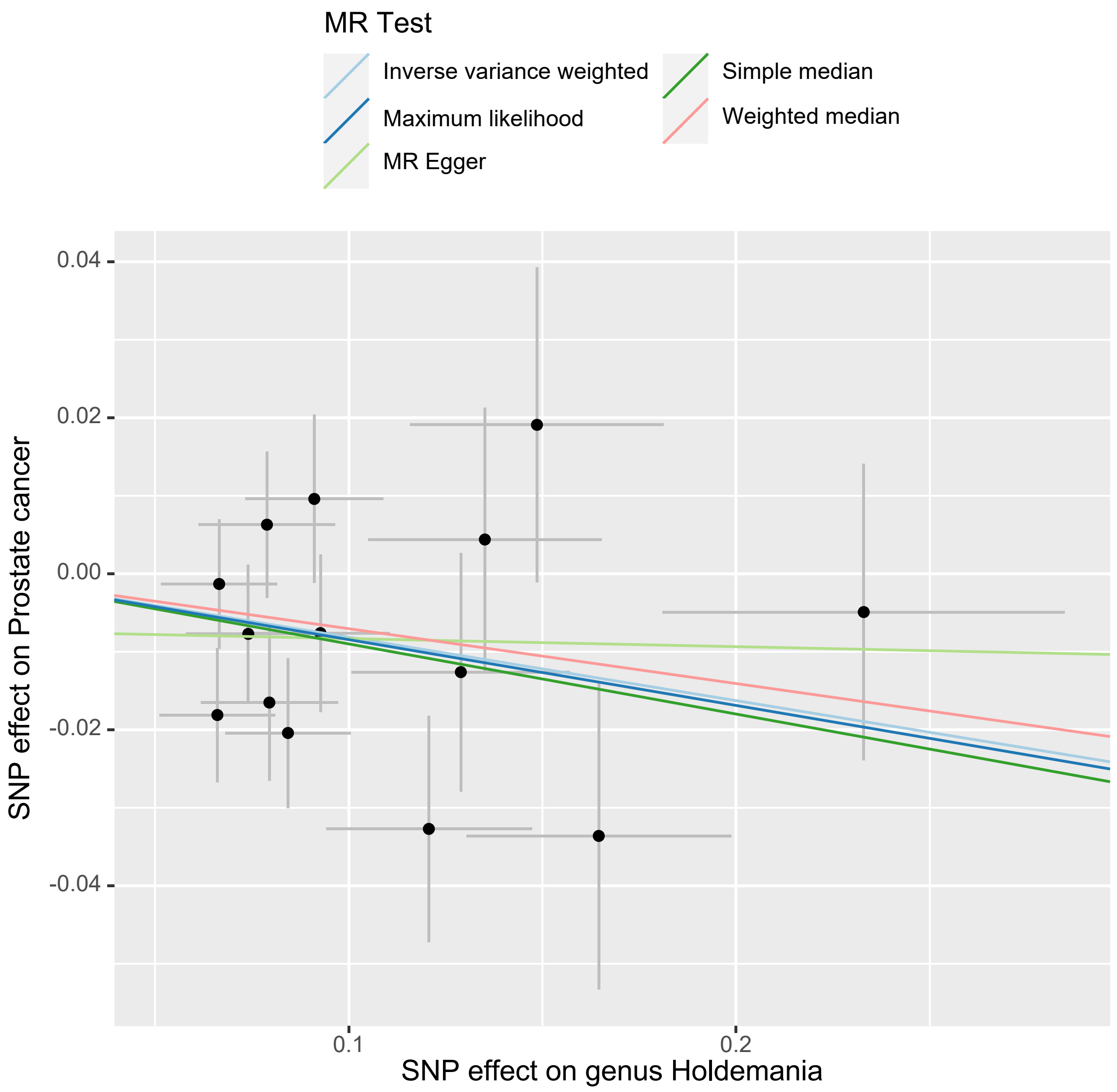

Figure S37. The scatter plot of genus Holdemania on prostate cancer in the PRACTICAL consortium

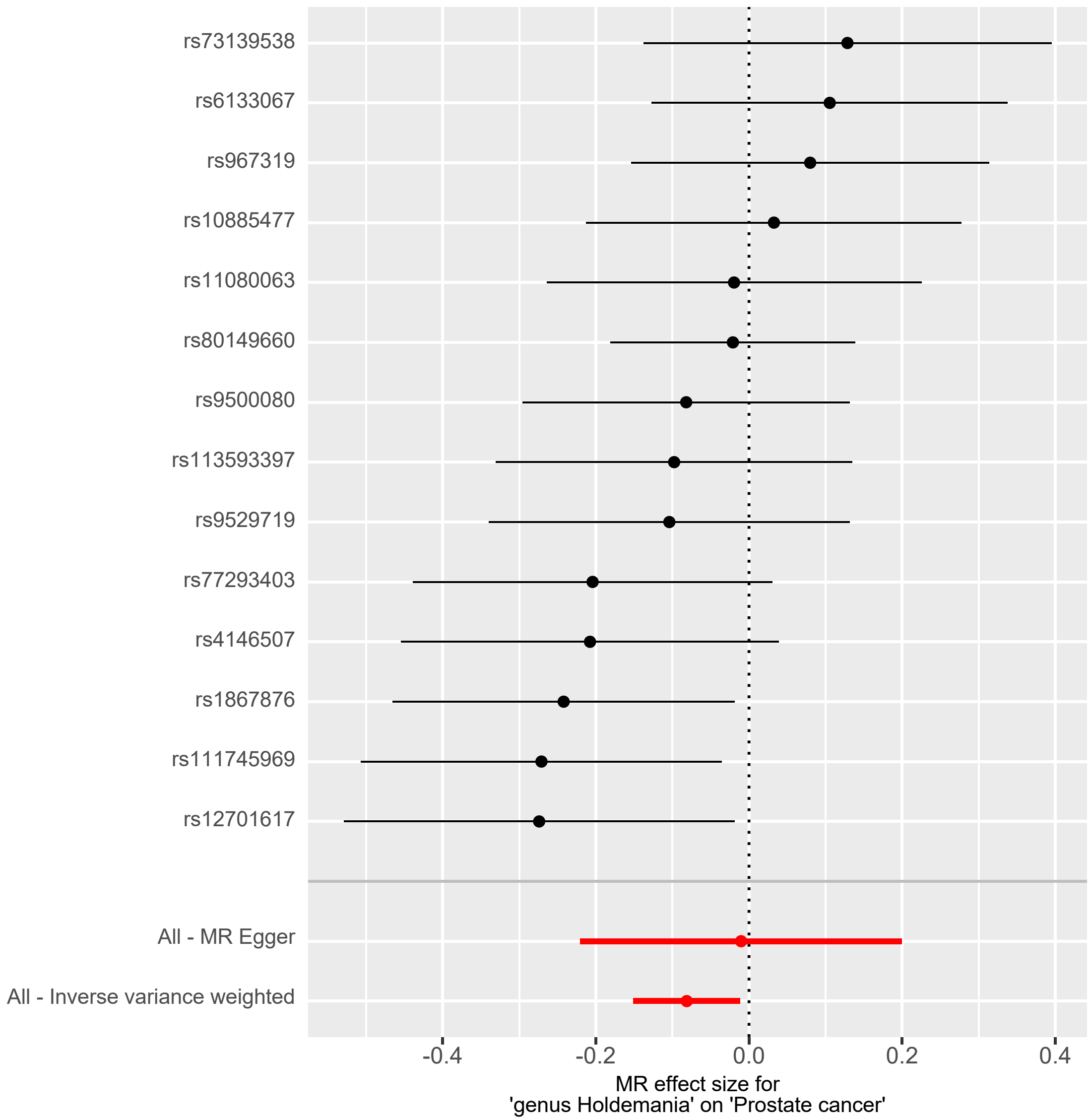

Figure S38. The forest plot of genus Holdemania on prostate cancer in the PRACTICAL consortium

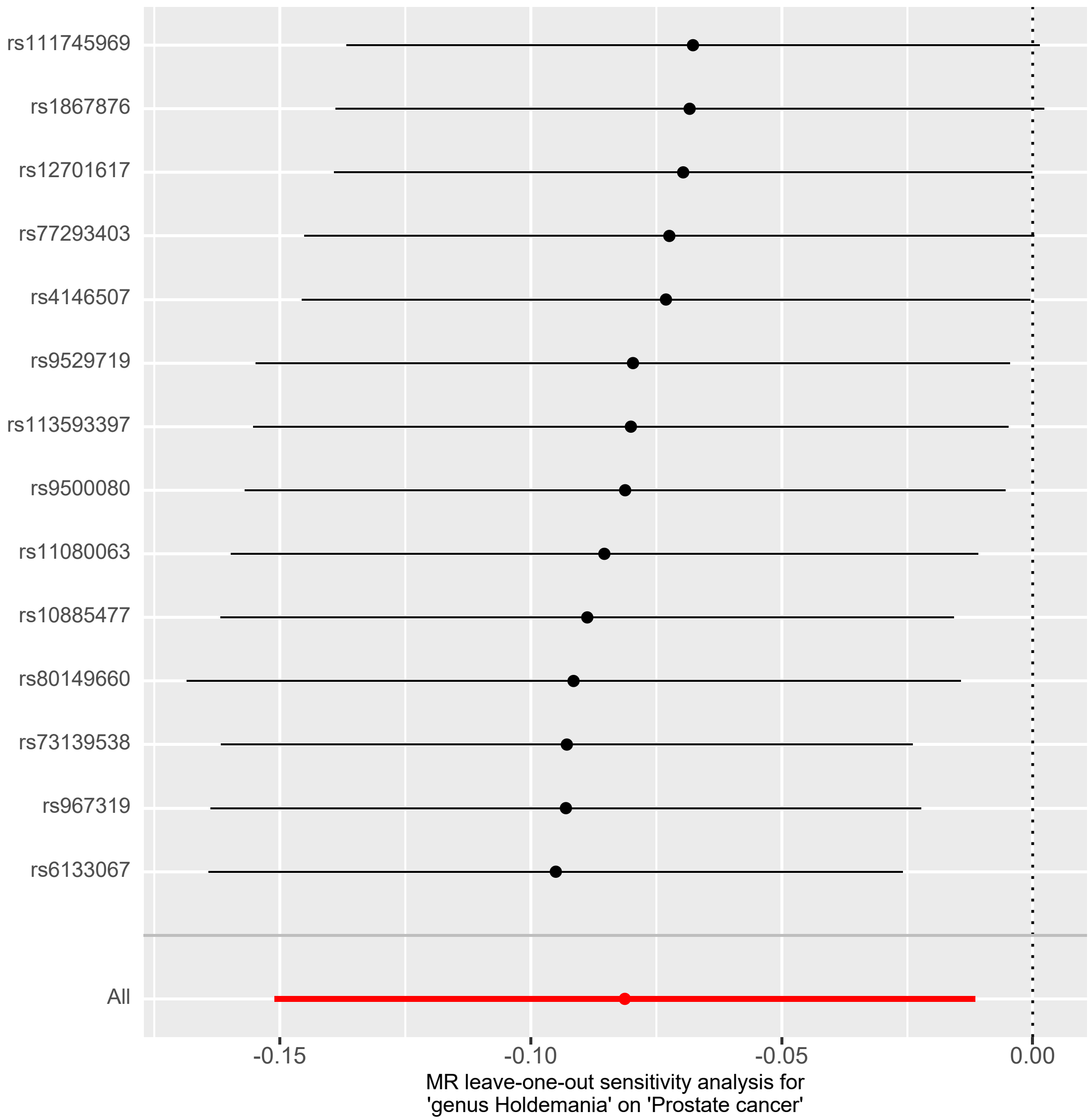

Figure S39. The leave-one-out test of genus Holdemania on prostate cancer in the PRACTICAL consortium

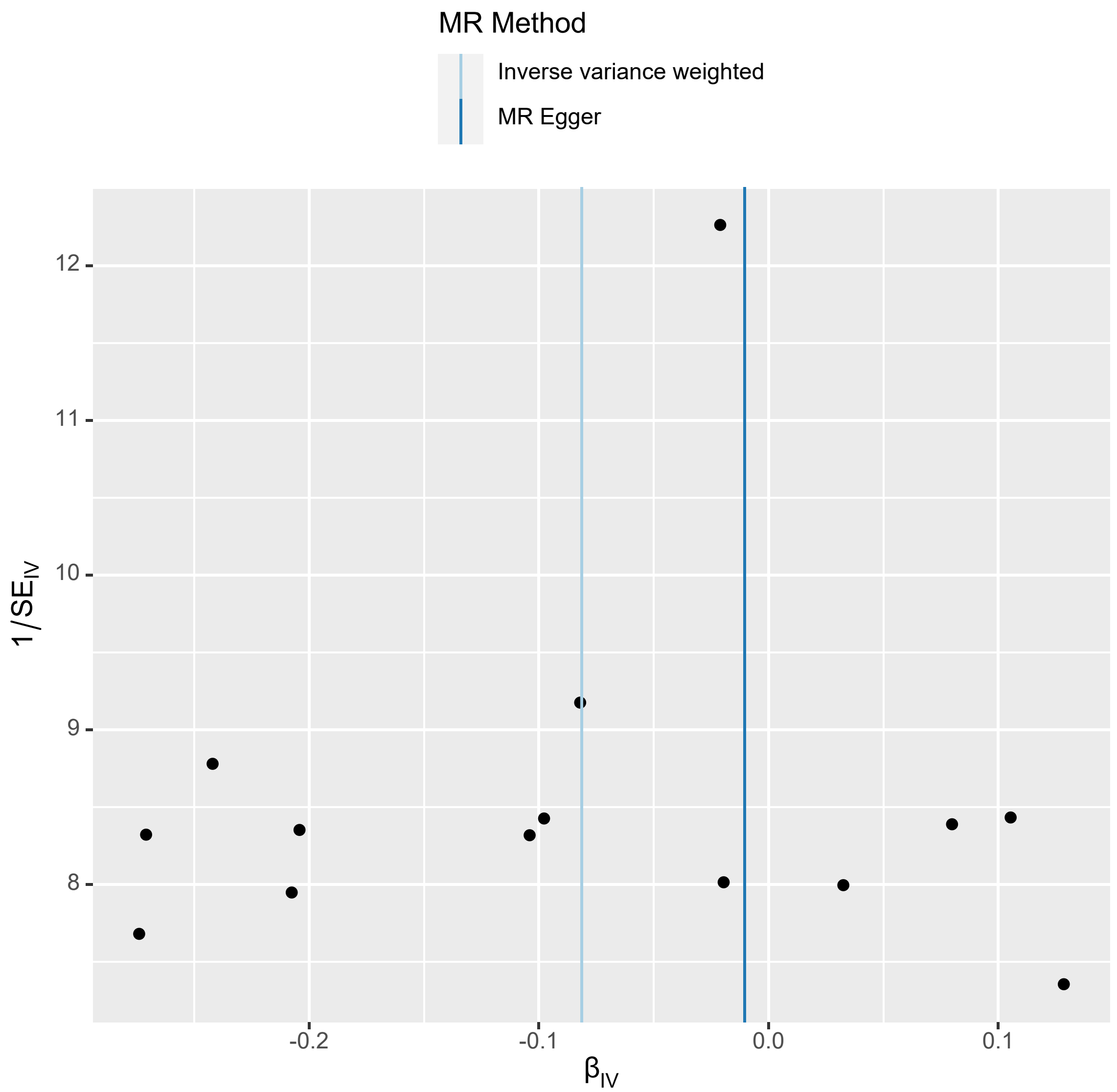

Figure S40. The funnel plot of genus Holdemania on prostate cancer in the PRACTICAL consortium

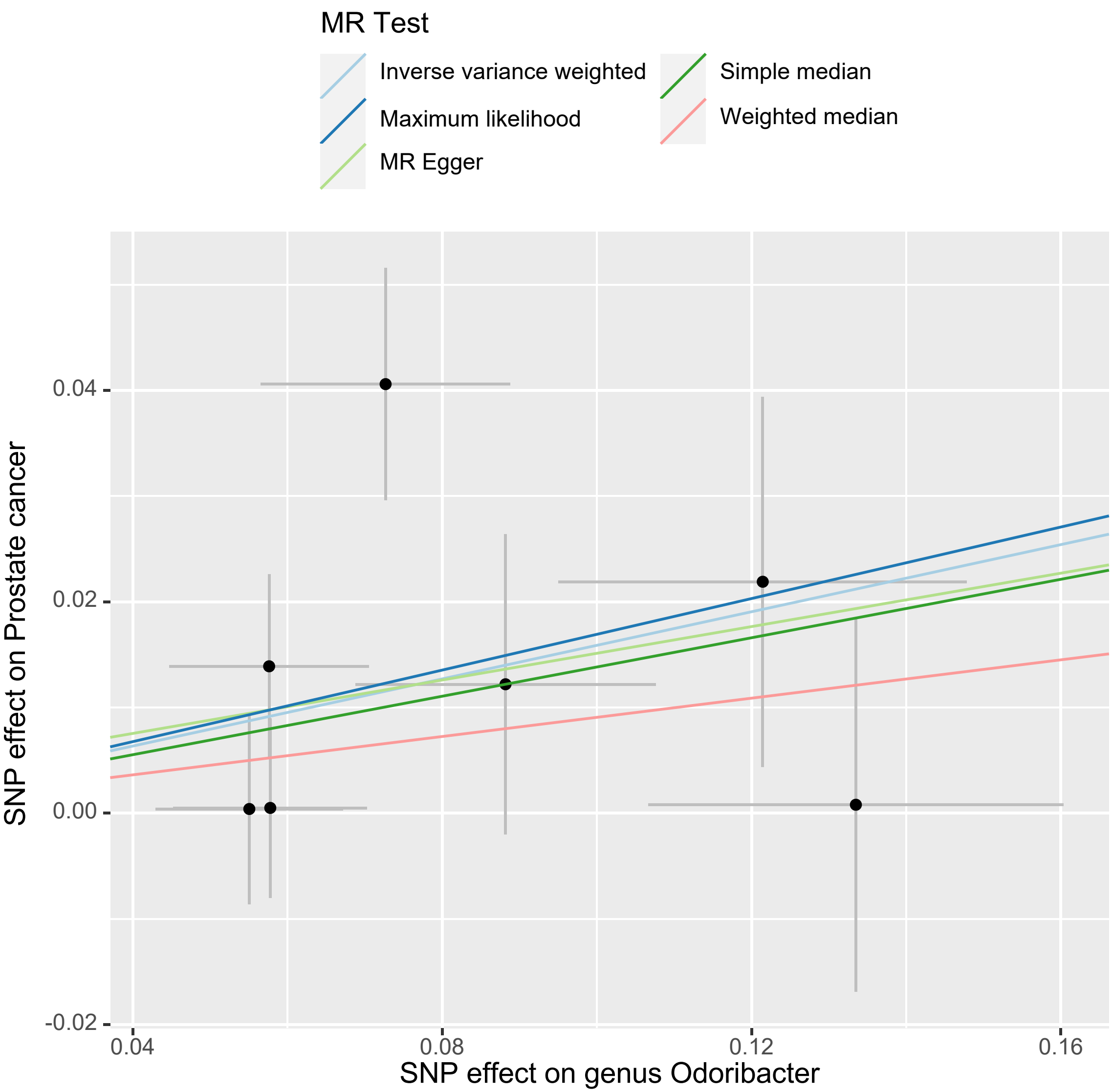

Figure S41. The scatter plot of genus Odoribacter on prostate cancer in the PRACTICAL consortium

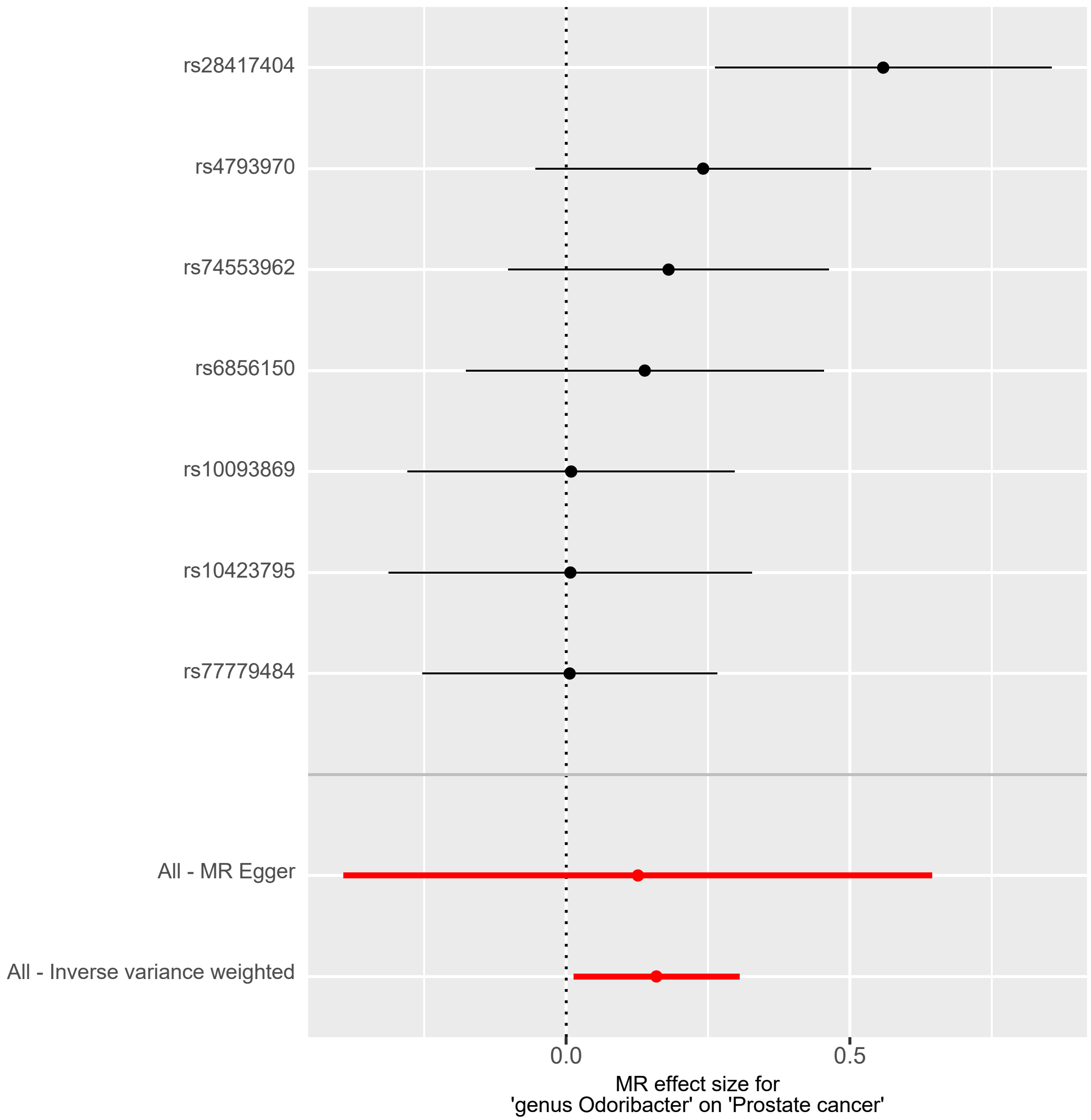

Figure S42. The forest plot of genus Odoribacter on prostate cancer in the PRACTICAL consortium

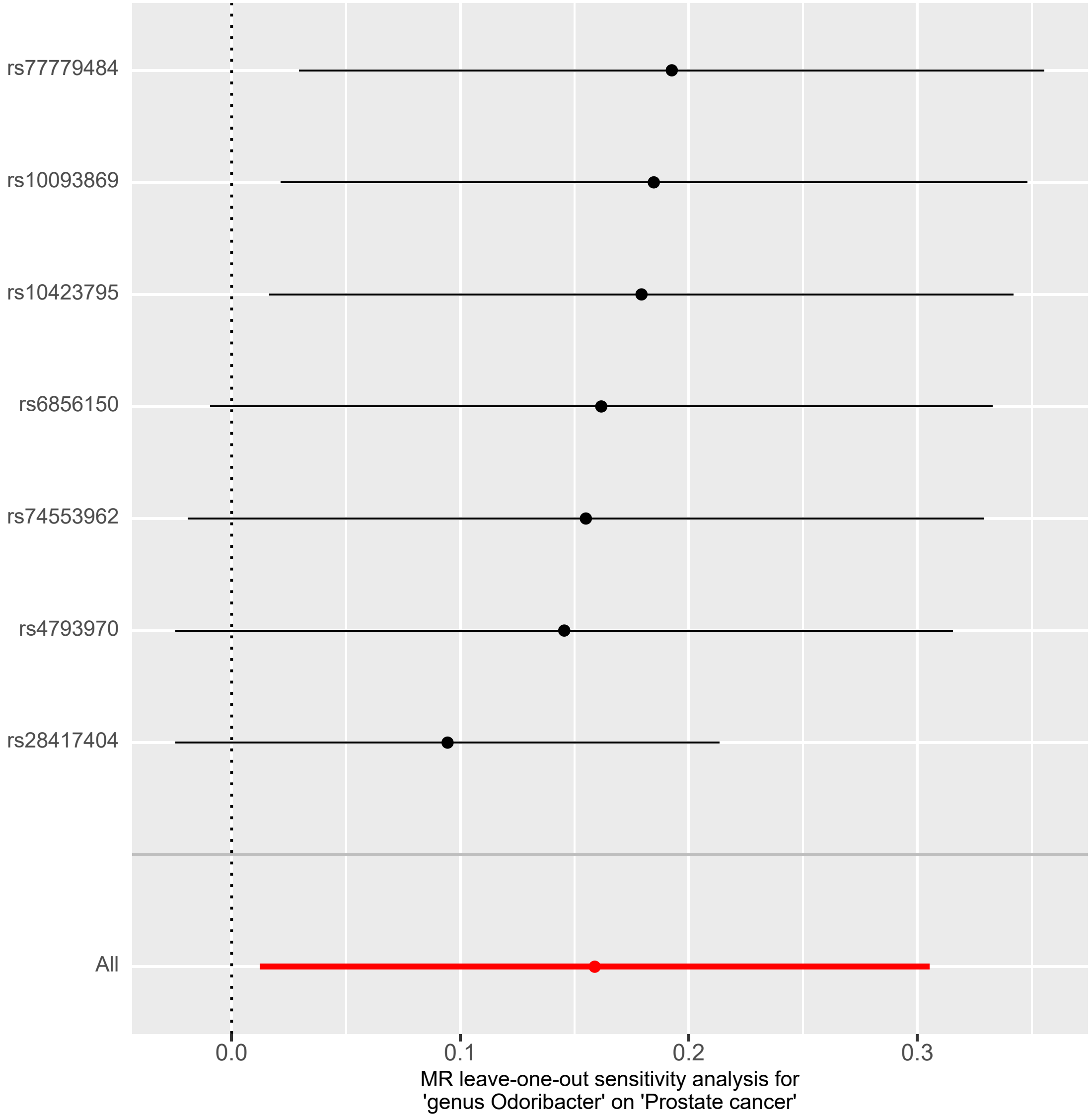

Figure S43. The leave-one-out test of genus Odoribacter on prostate cancer in the PRACTICAL consortium

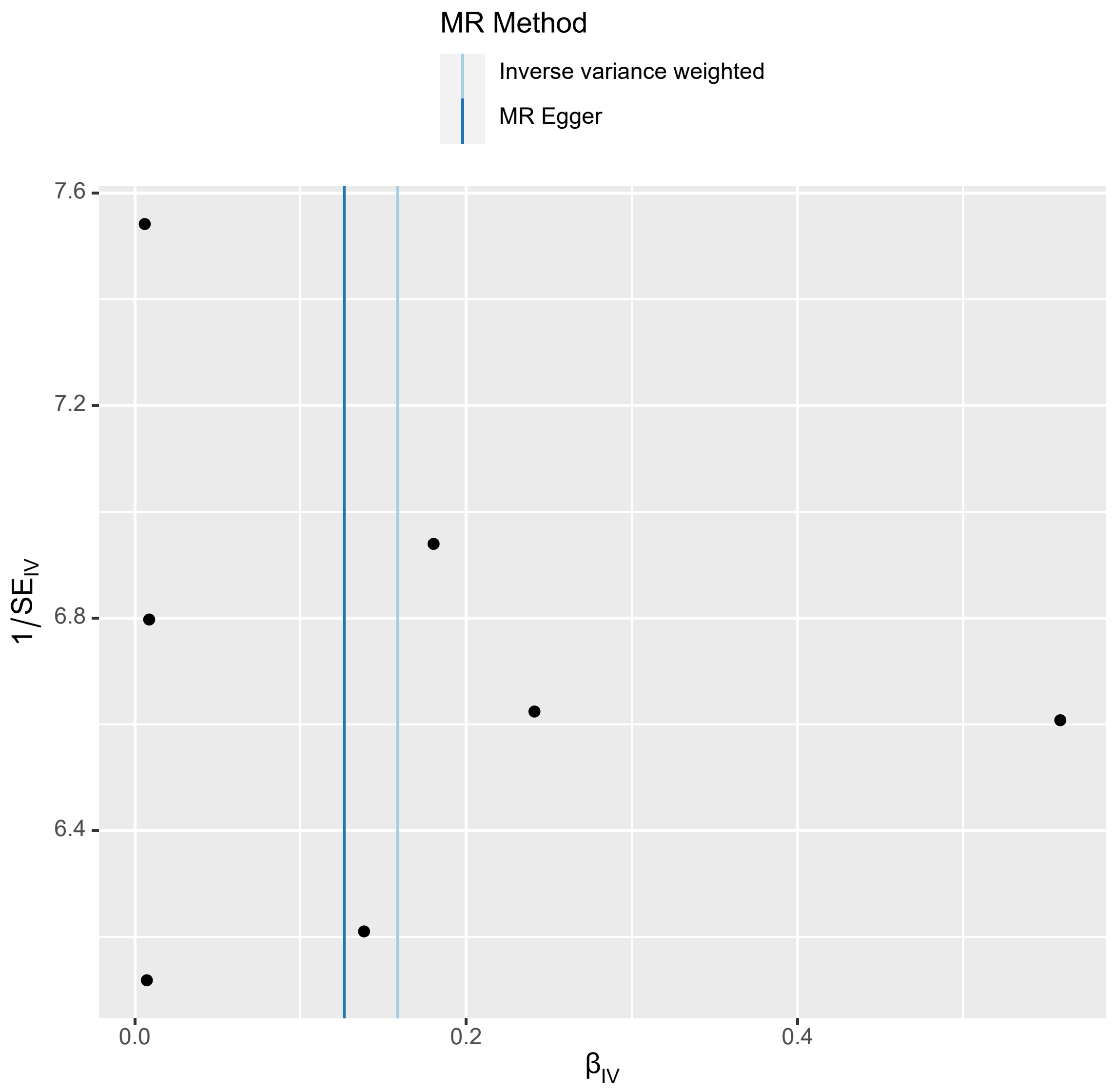

Figure S44. The funnel plot of genus Odoribacter on prostate cancer in the PRACTICAL consortium

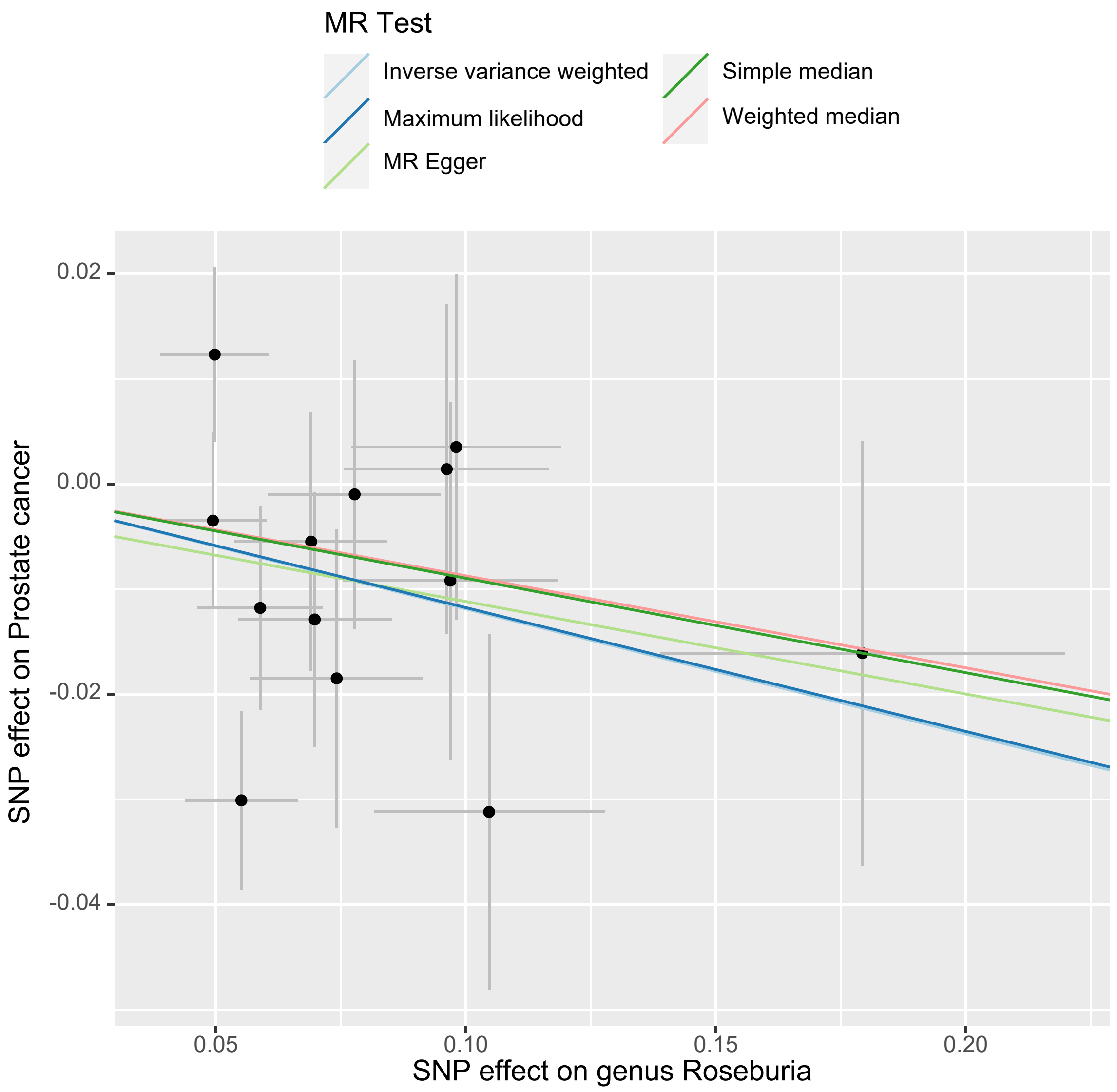

Figure S45. The scatter plot of genus Roseburia on prostate cancer in the PRACTICAL consortium

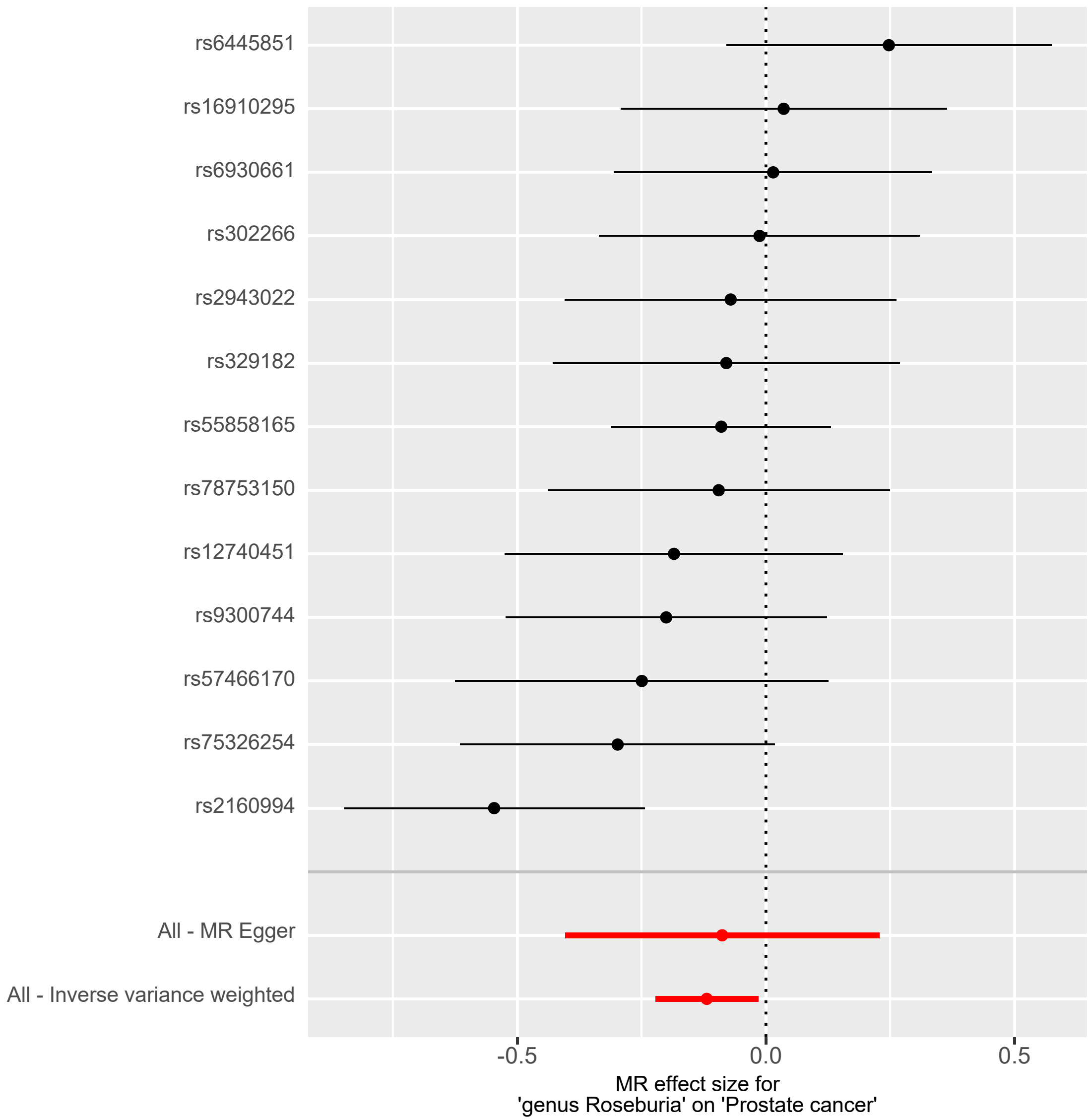

Figure S46. The forest plot of genus Roseburia on prostate cancer in the PRACTICAL consortium

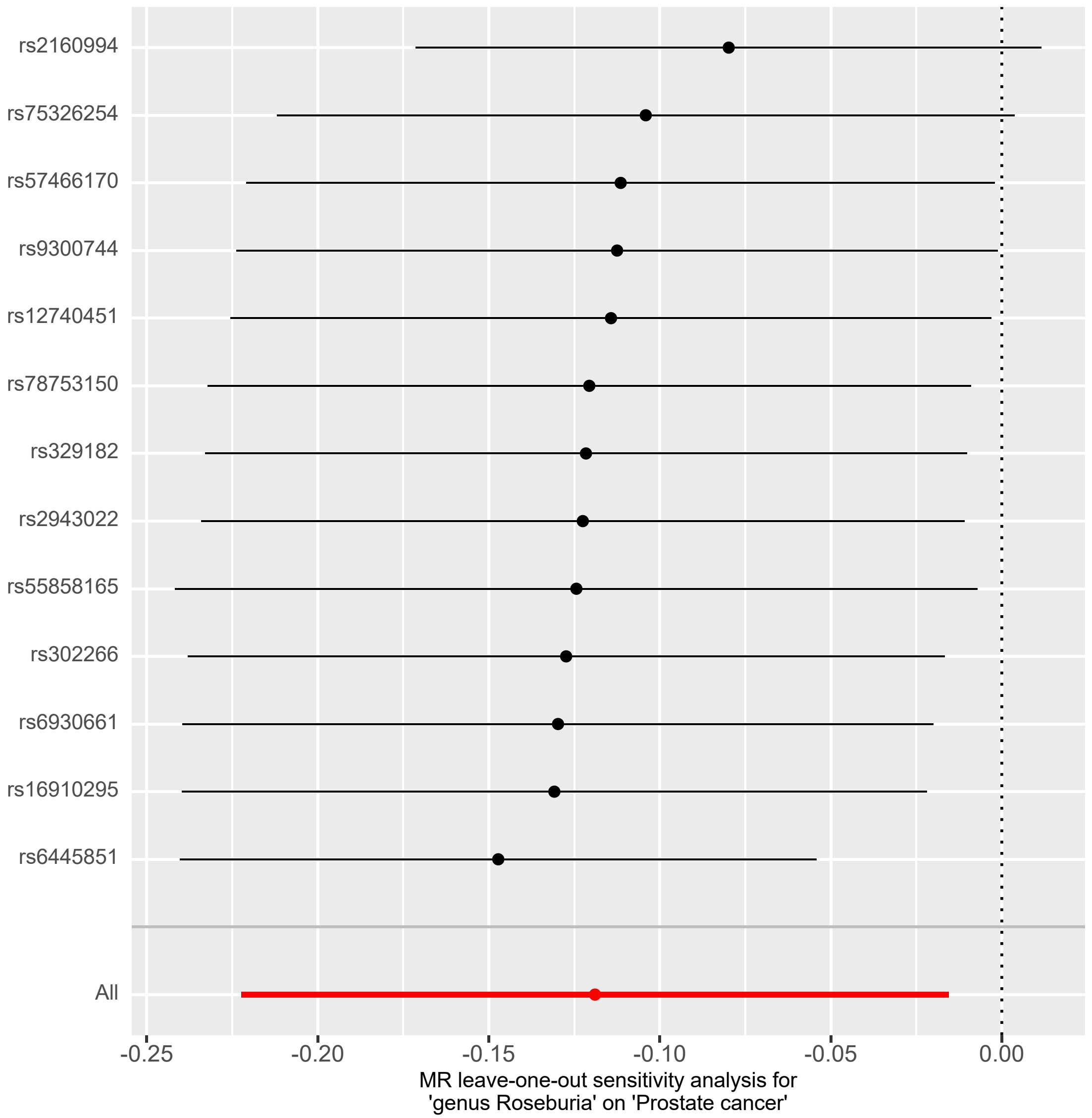

Figure S47. The leave-one-out test of genus Roseburia on prostate cancer in the PRACTICAL consortium

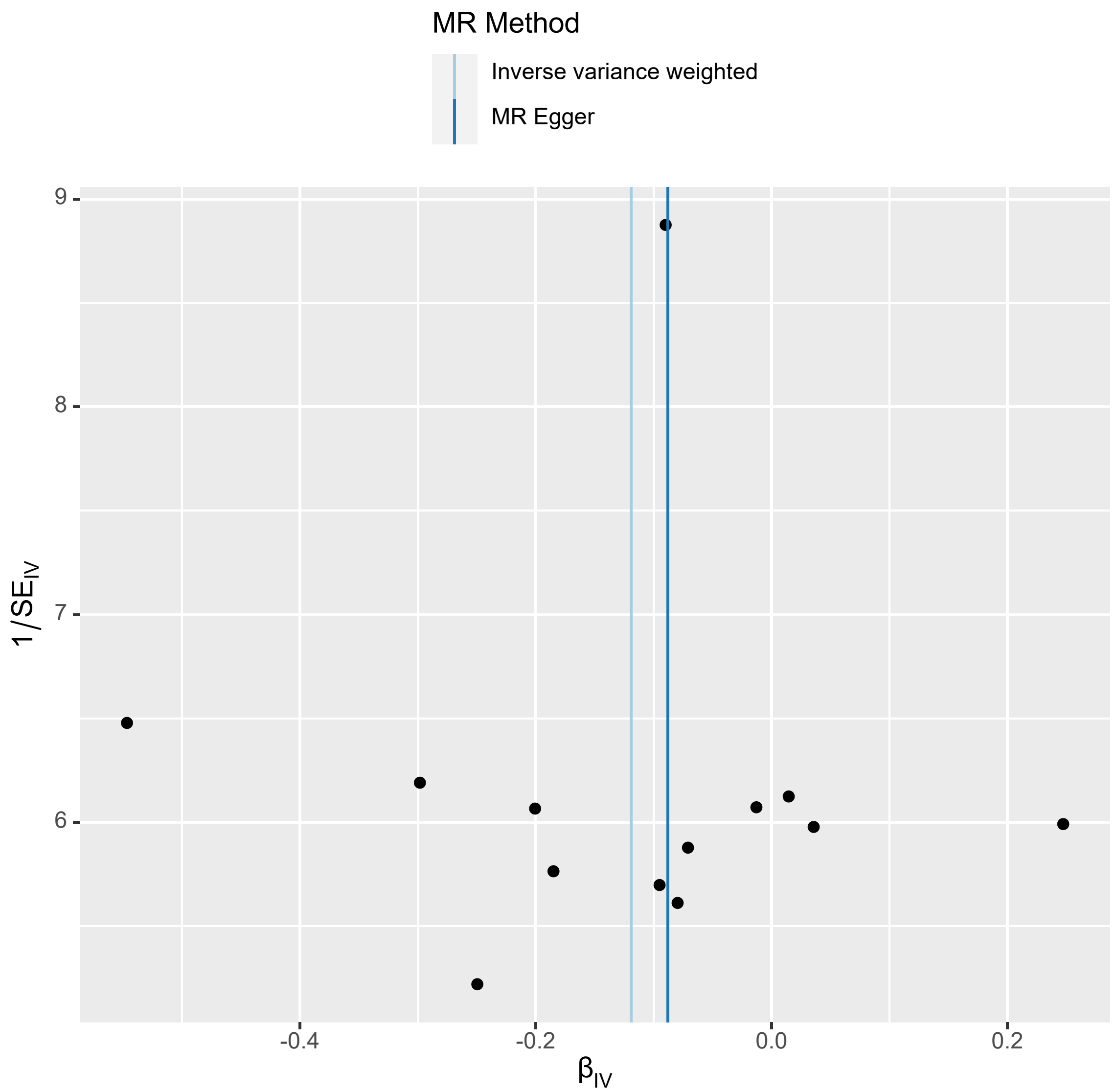

Figure S48. The funnel plot of genus Roseburia on prostate cancer in the PRACTICAL consortium

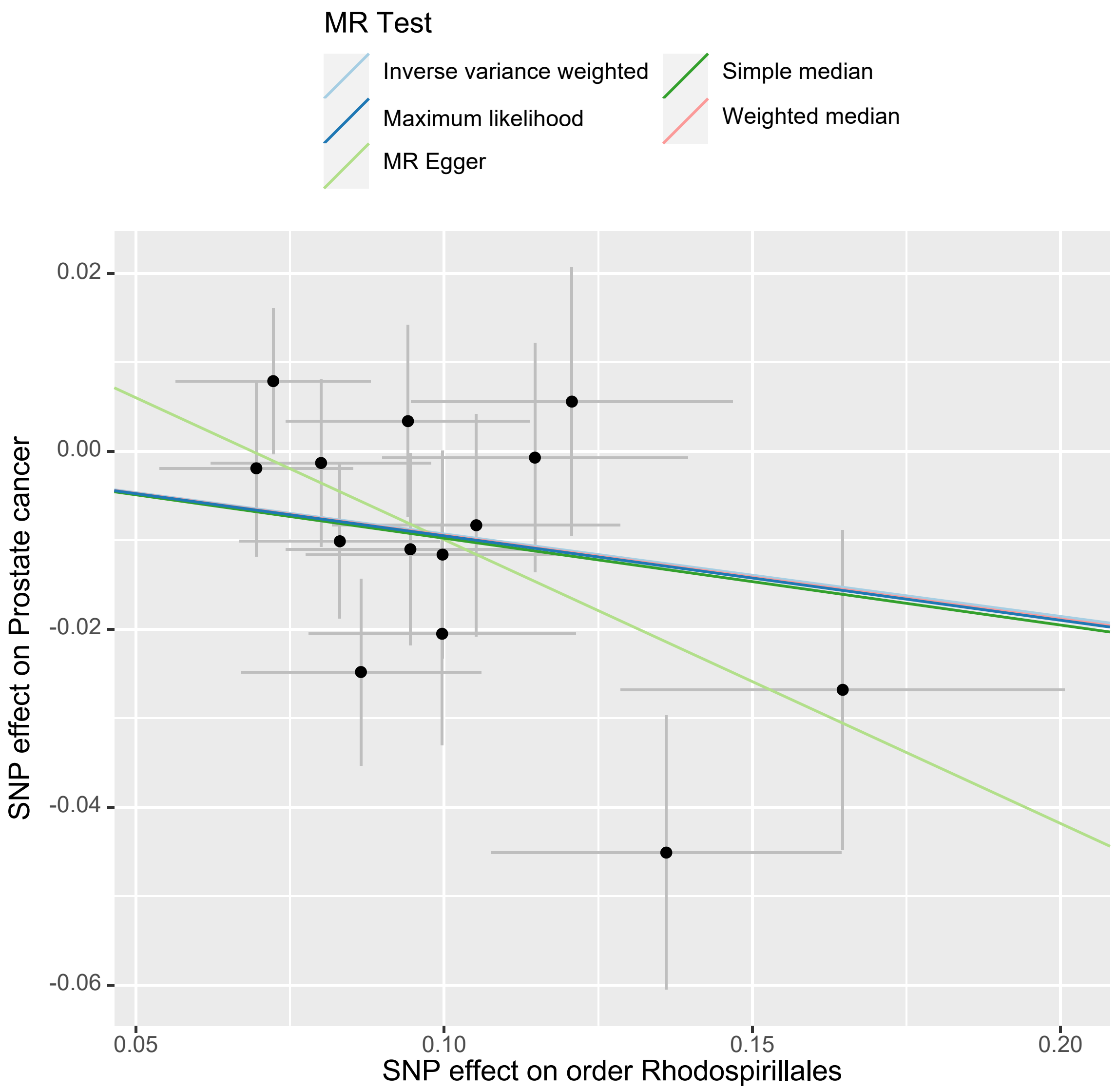

Figure S49. The scatter plot of order Rhodospirillales on prostate cancer in the PRACTICAL consortium

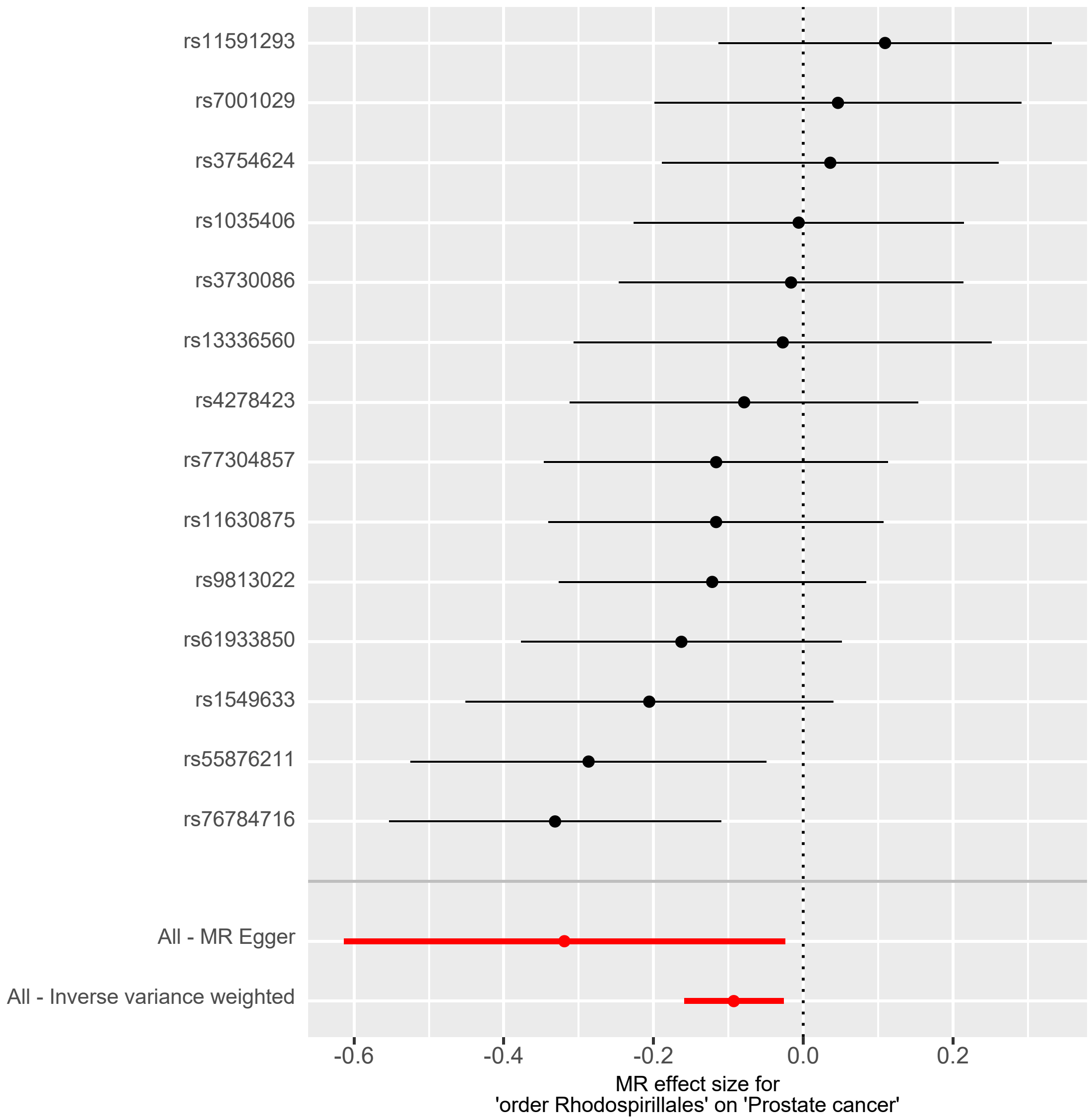

Figure S50. The forest plot of order Rhodospirillales on prostate cancer in the PRACTICAL consortium

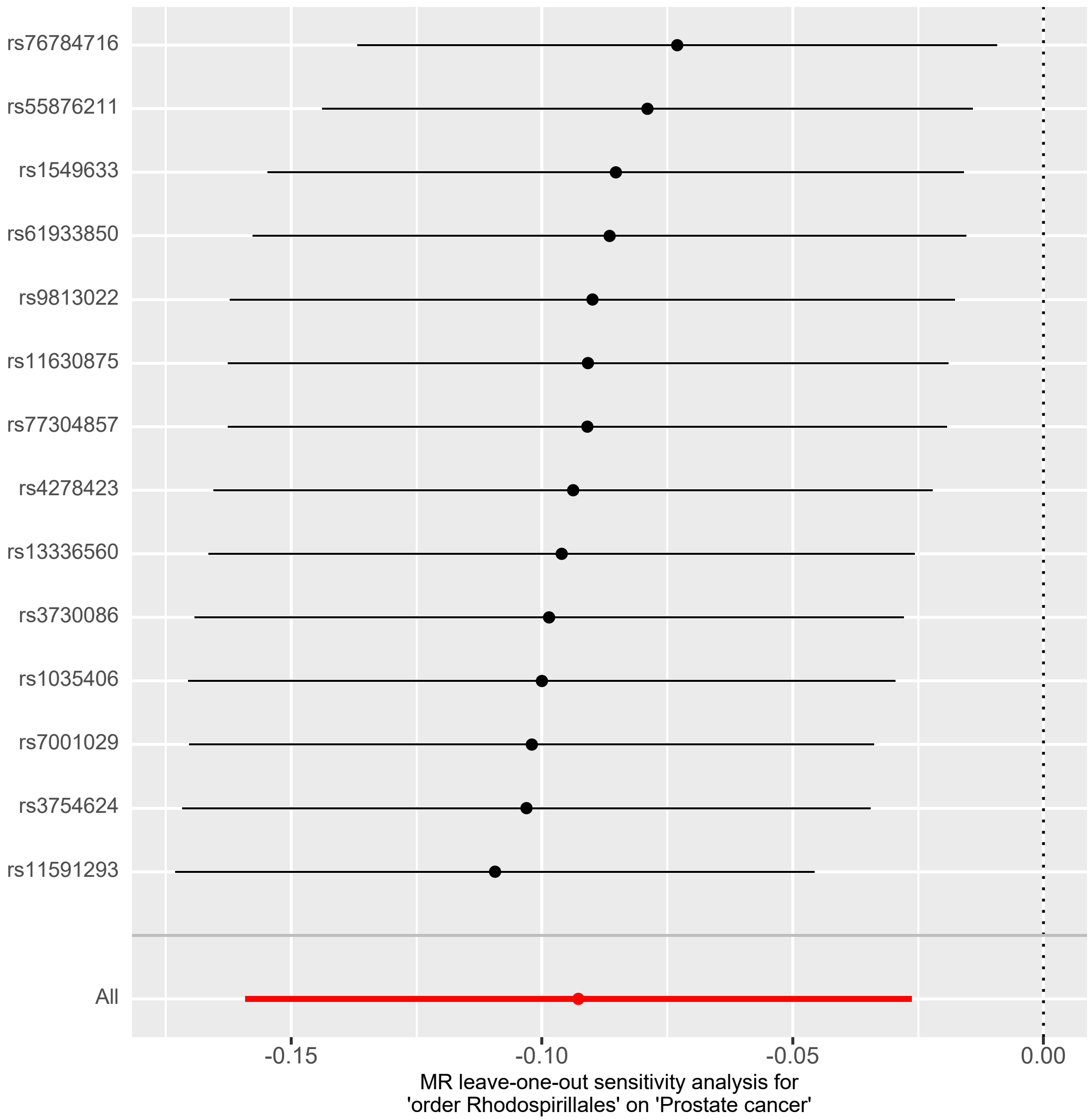

Figure S51. The leave-one-out test of order Rhodospirillales on prostate cancer in the PRACTICAL consortium

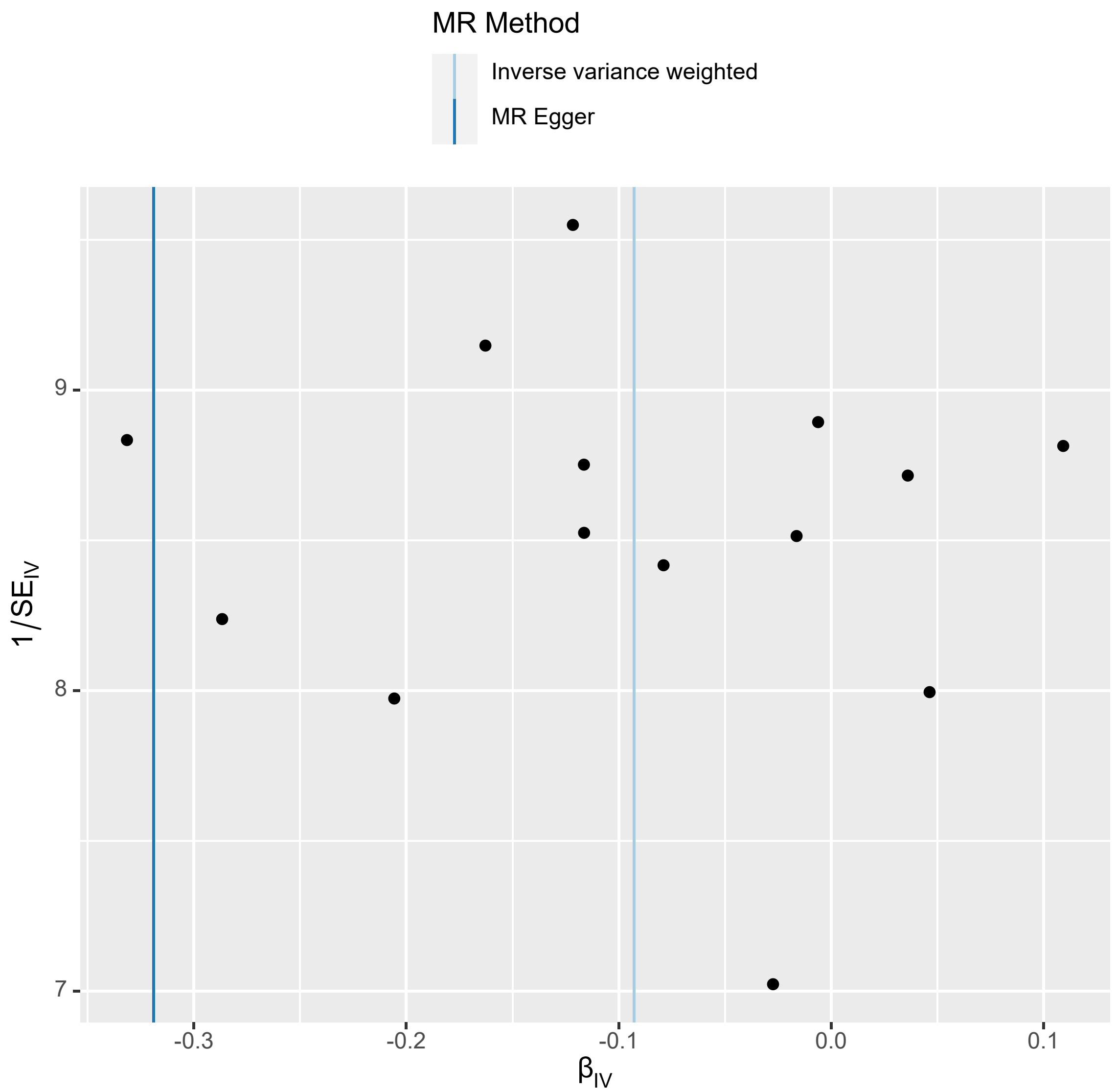

Figure S52. The funnel plot of order Rhodospirillales on prostate cancer in the PRACTICAL consortium

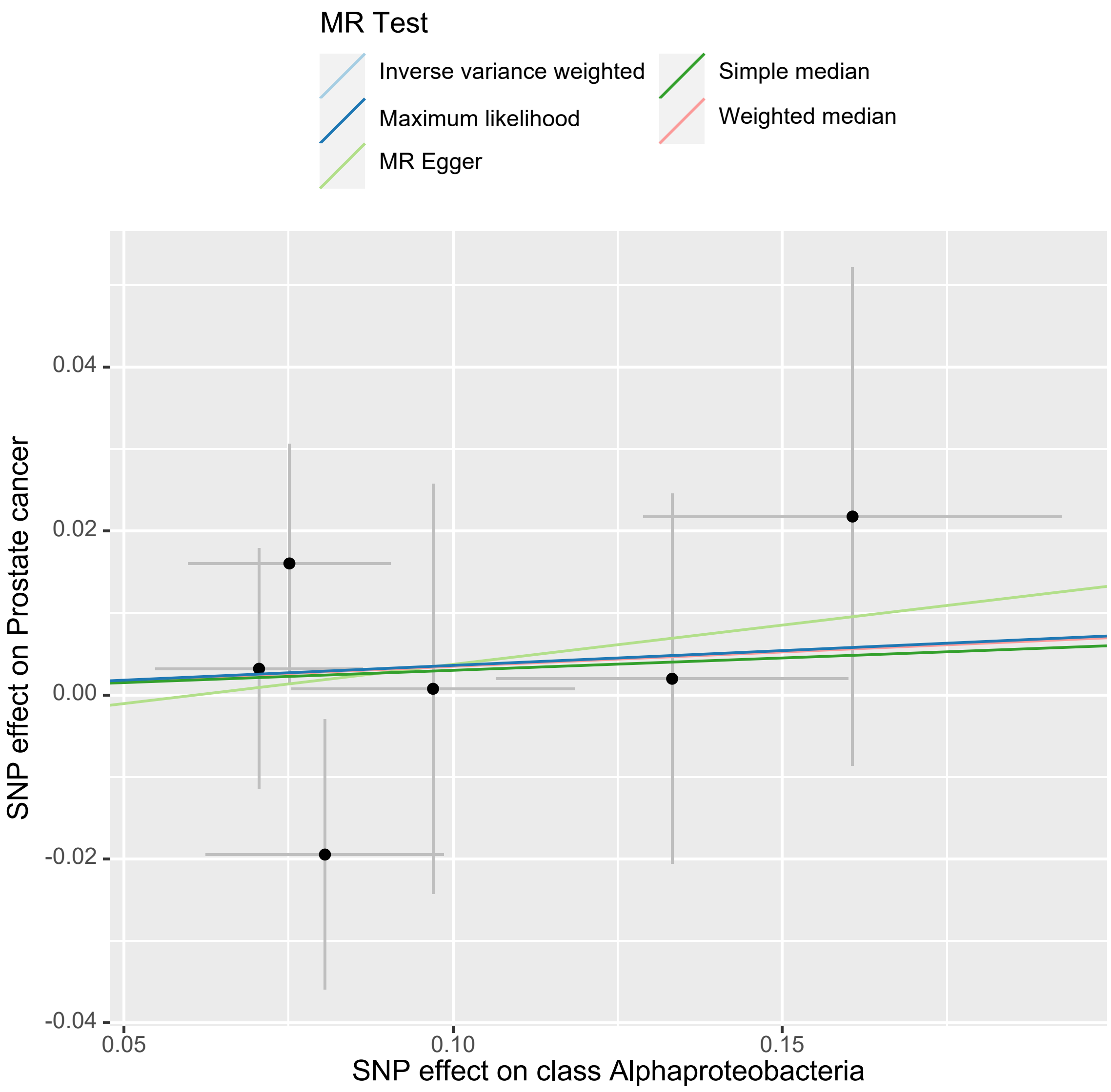

Figure S53. The scatter plot of class Alphaproteobacteria on prostate cancer in the FinnGen consortium

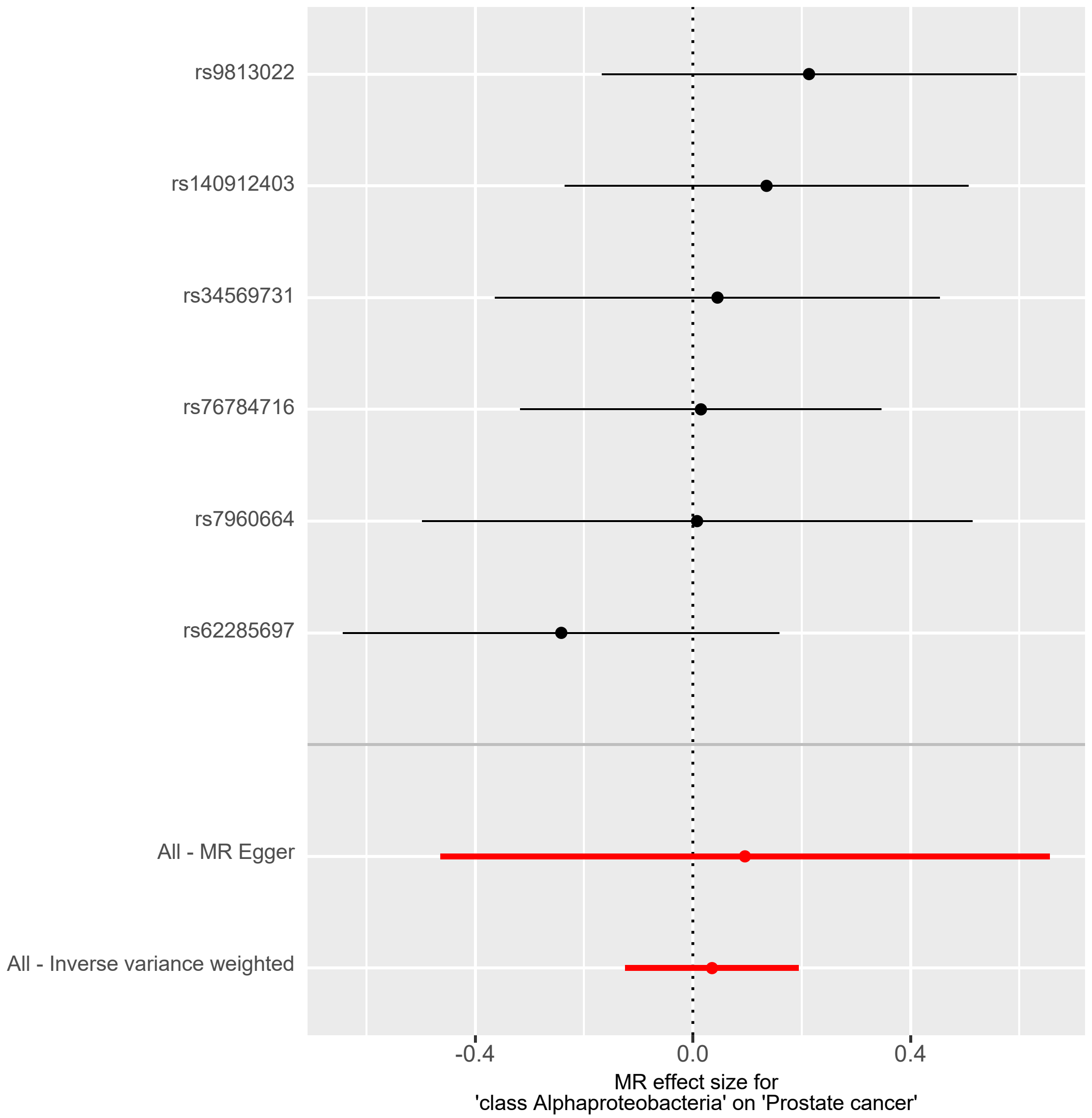

Figure S54. The forest plot of class Alphaproteobacteria on prostate cancer in the FinnGen consortium

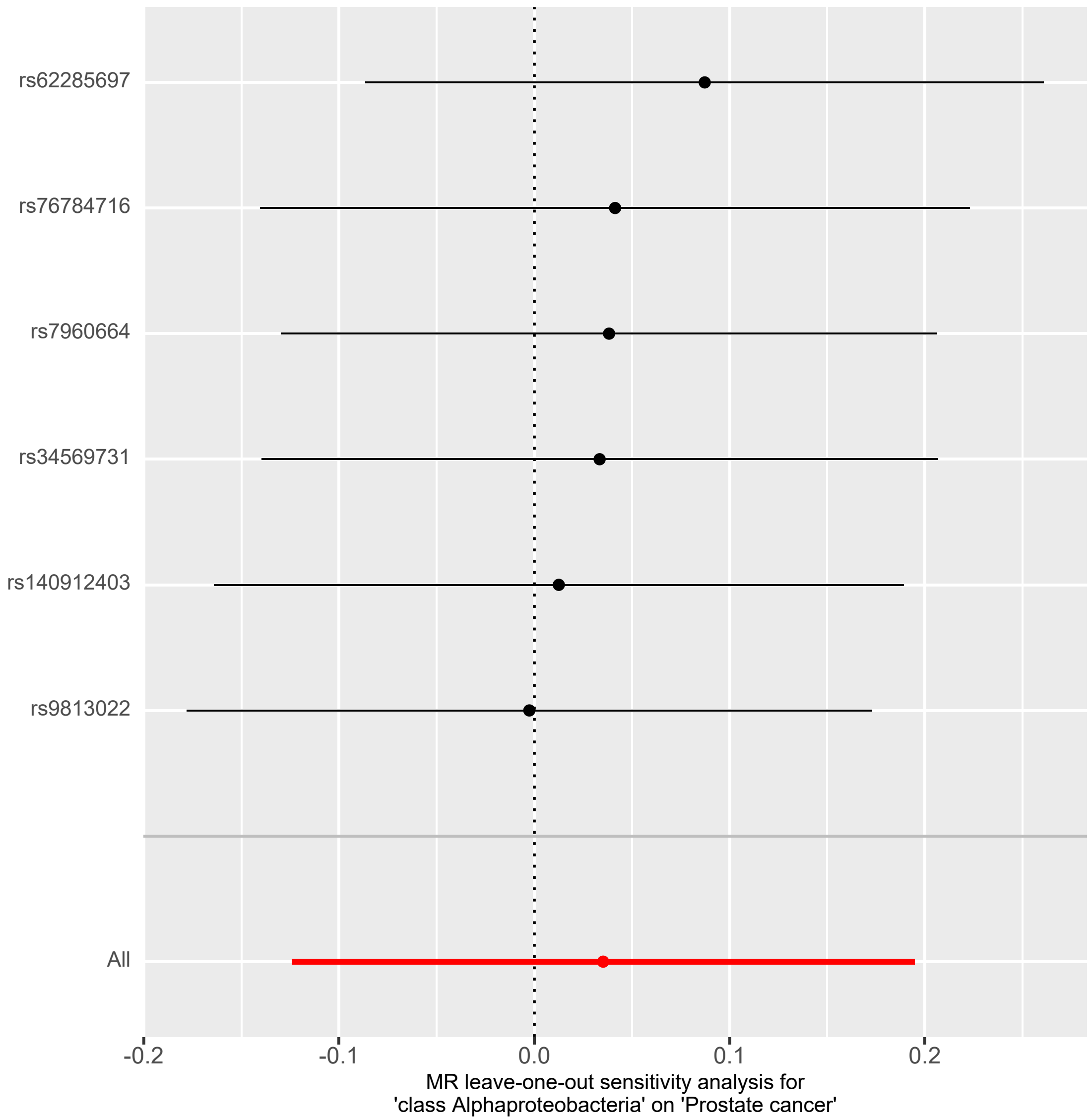

Figure S55. The leave-one-out test of class Alphaproteobacteria on prostate cancer in the FinnGen consortium

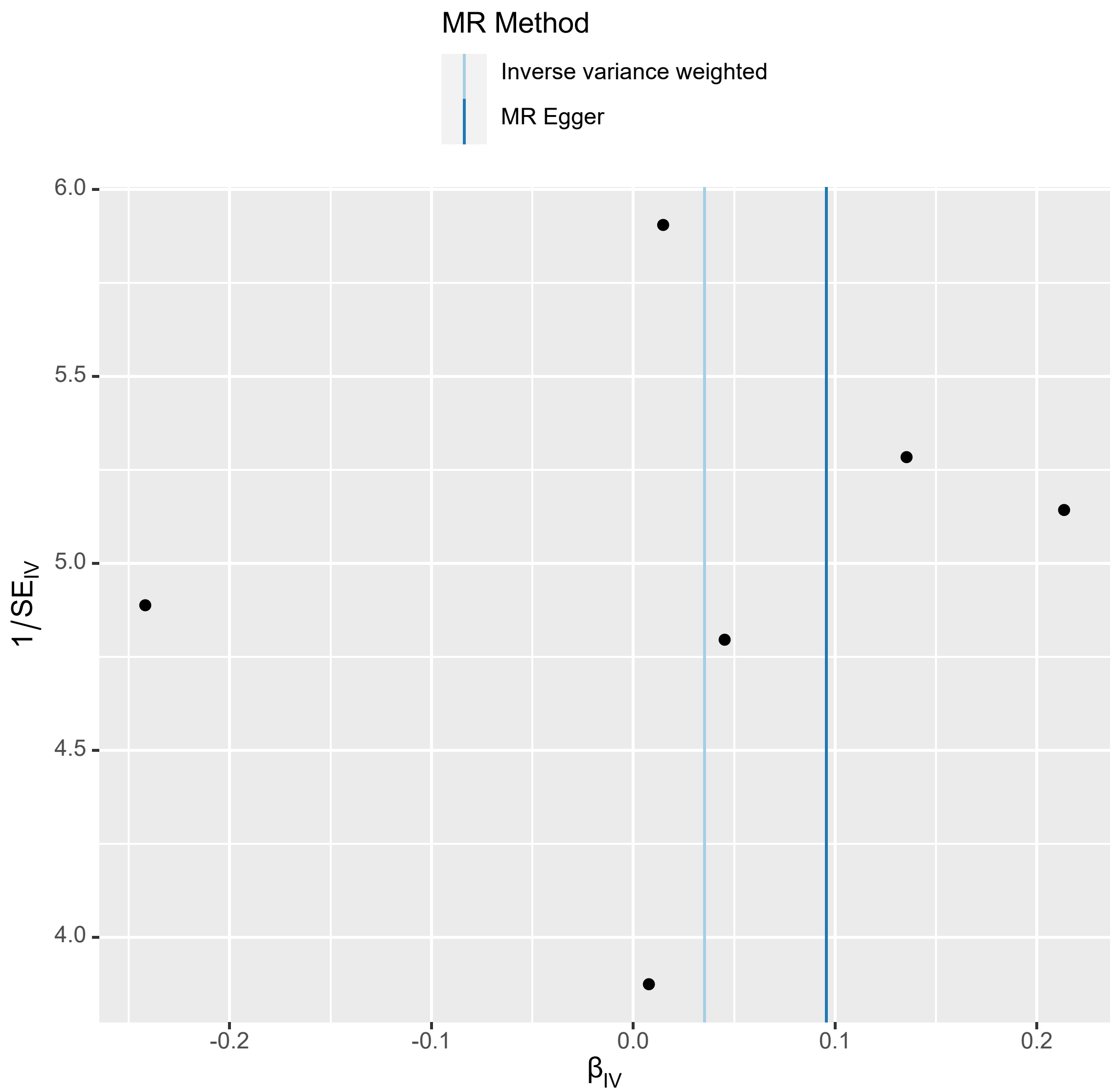

Figure S56. The funnel plot of class Alphaproteobacteria on prostate cancer in the FinnGen consortium

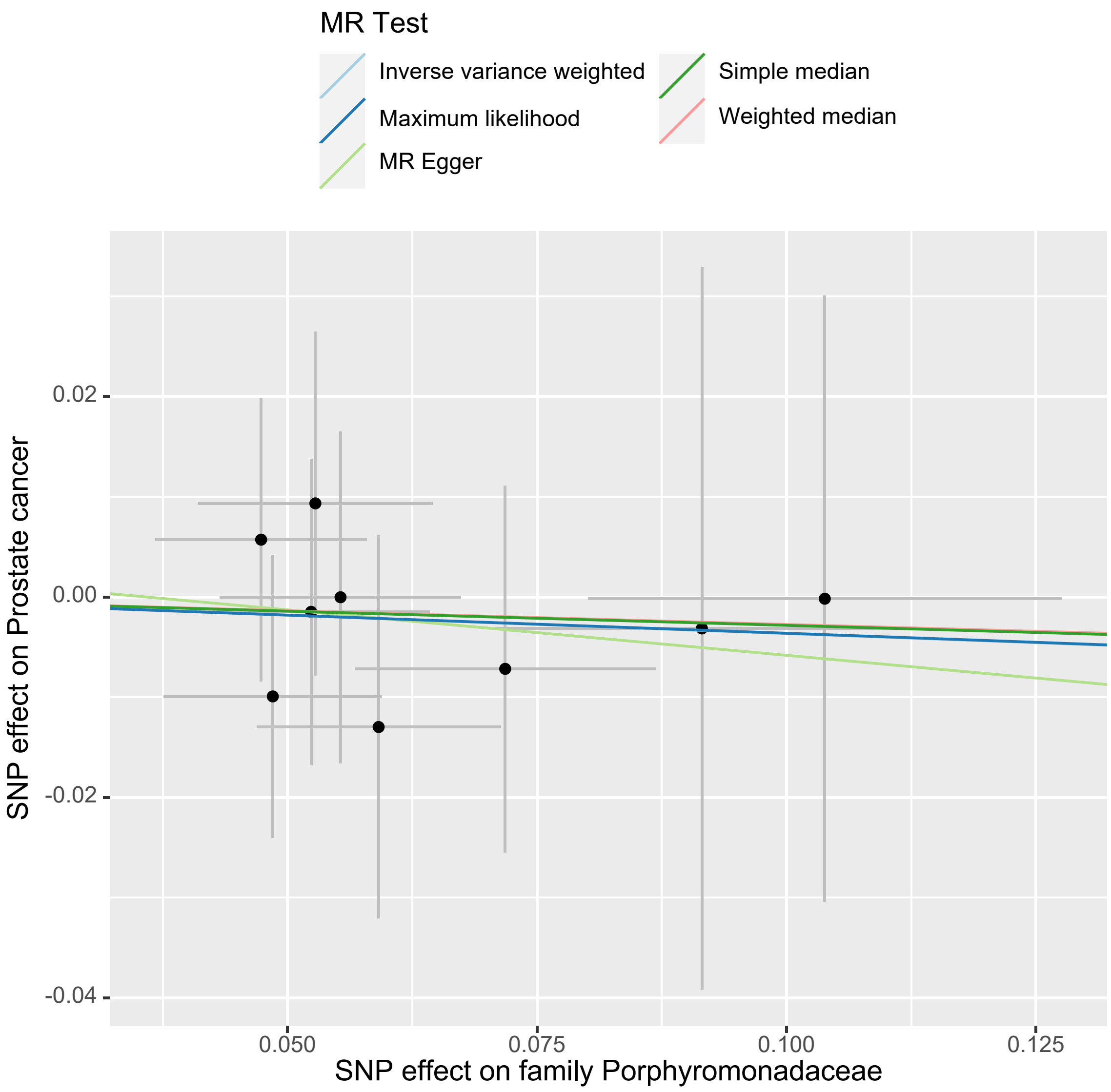

Figure S57. The scatter plot of family Porphyromonadaceae on prostate cancer in the FinnGen consortium

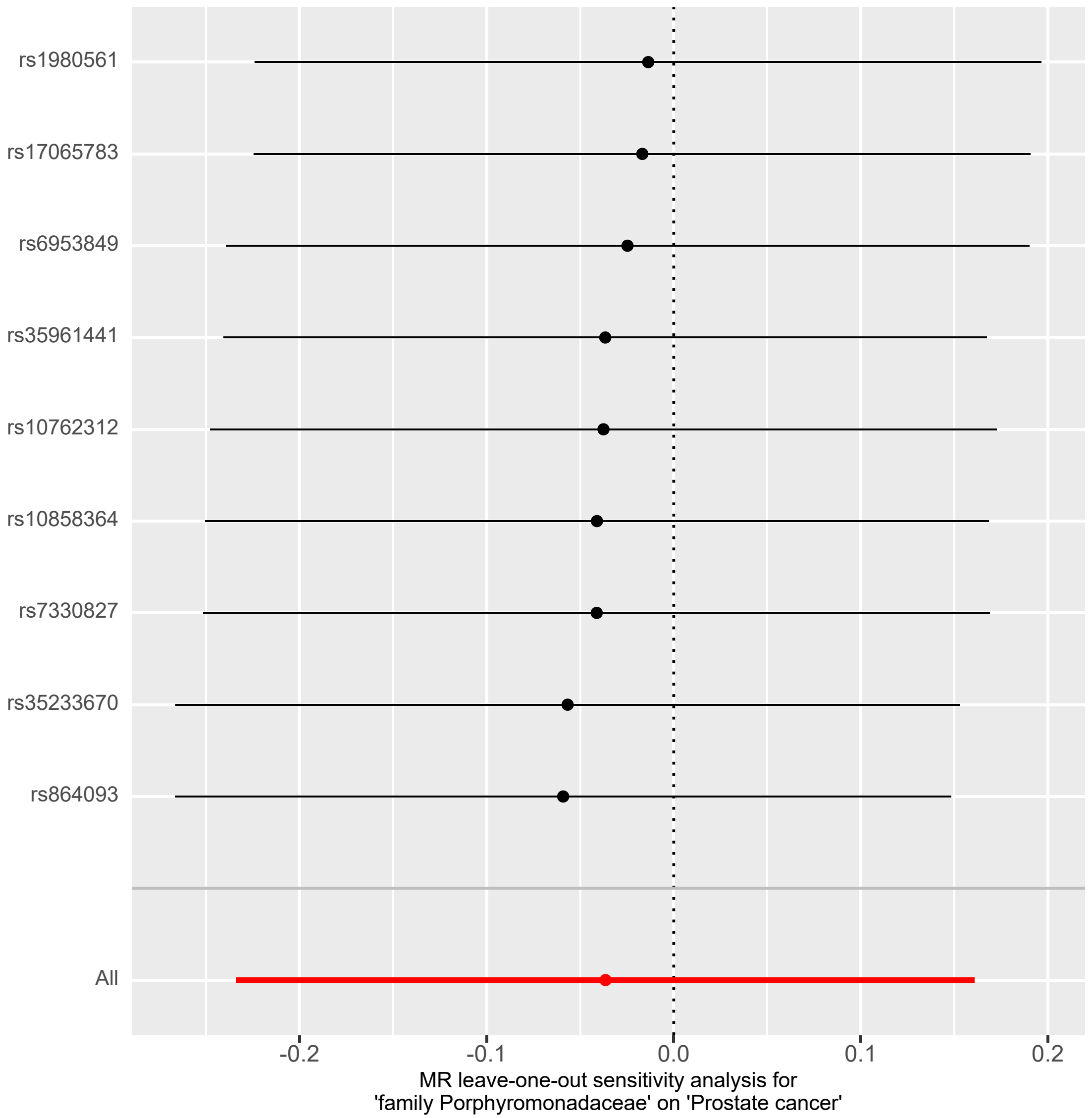

Figure S58. The forest plot of family Porphyromonadaceae on prostate cancer in the FinnGen consortium

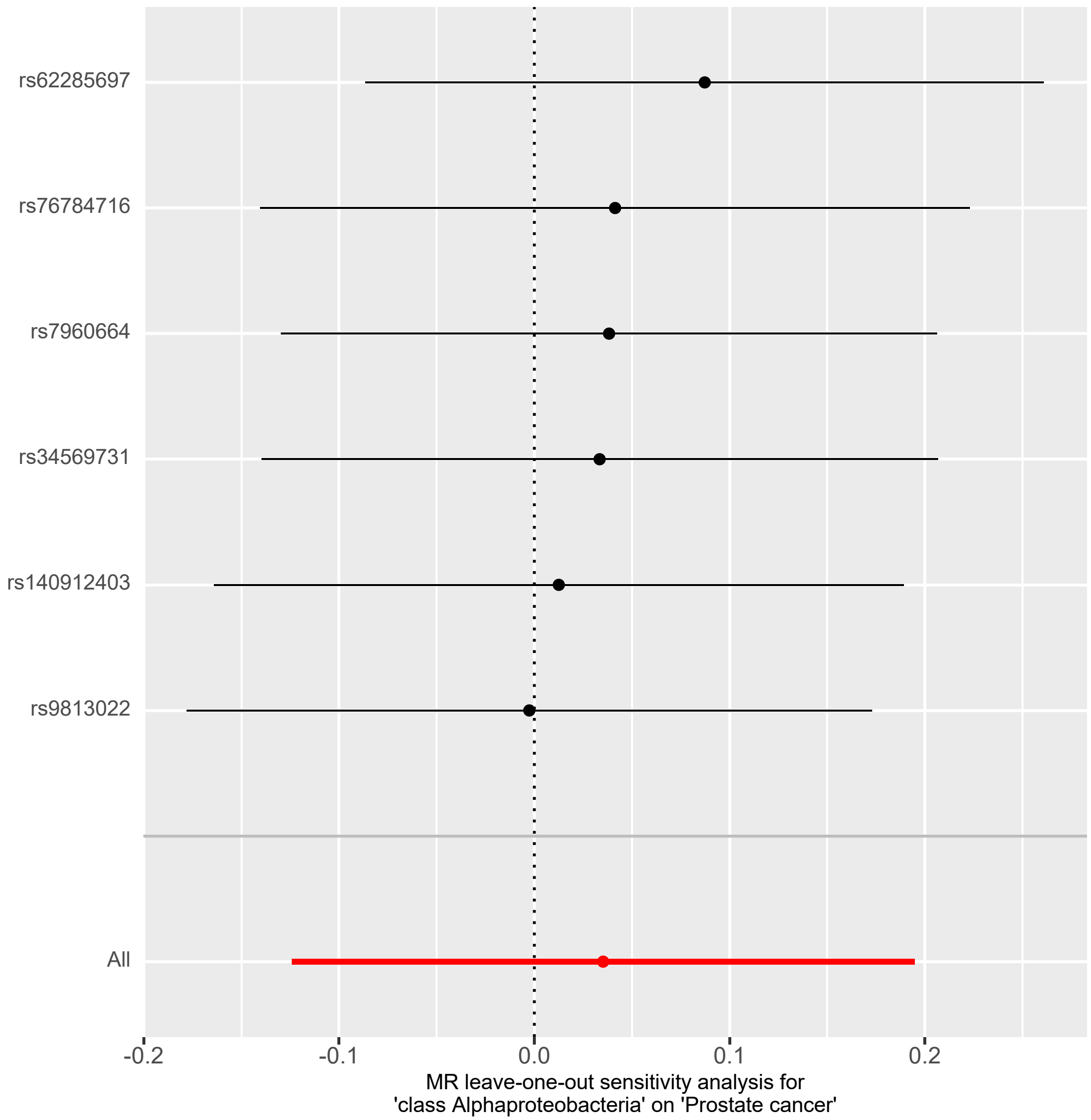

Figure S59. The leave-one-out test of family Porphyromonadaceae on prostate cancer in the FinnGen consortium

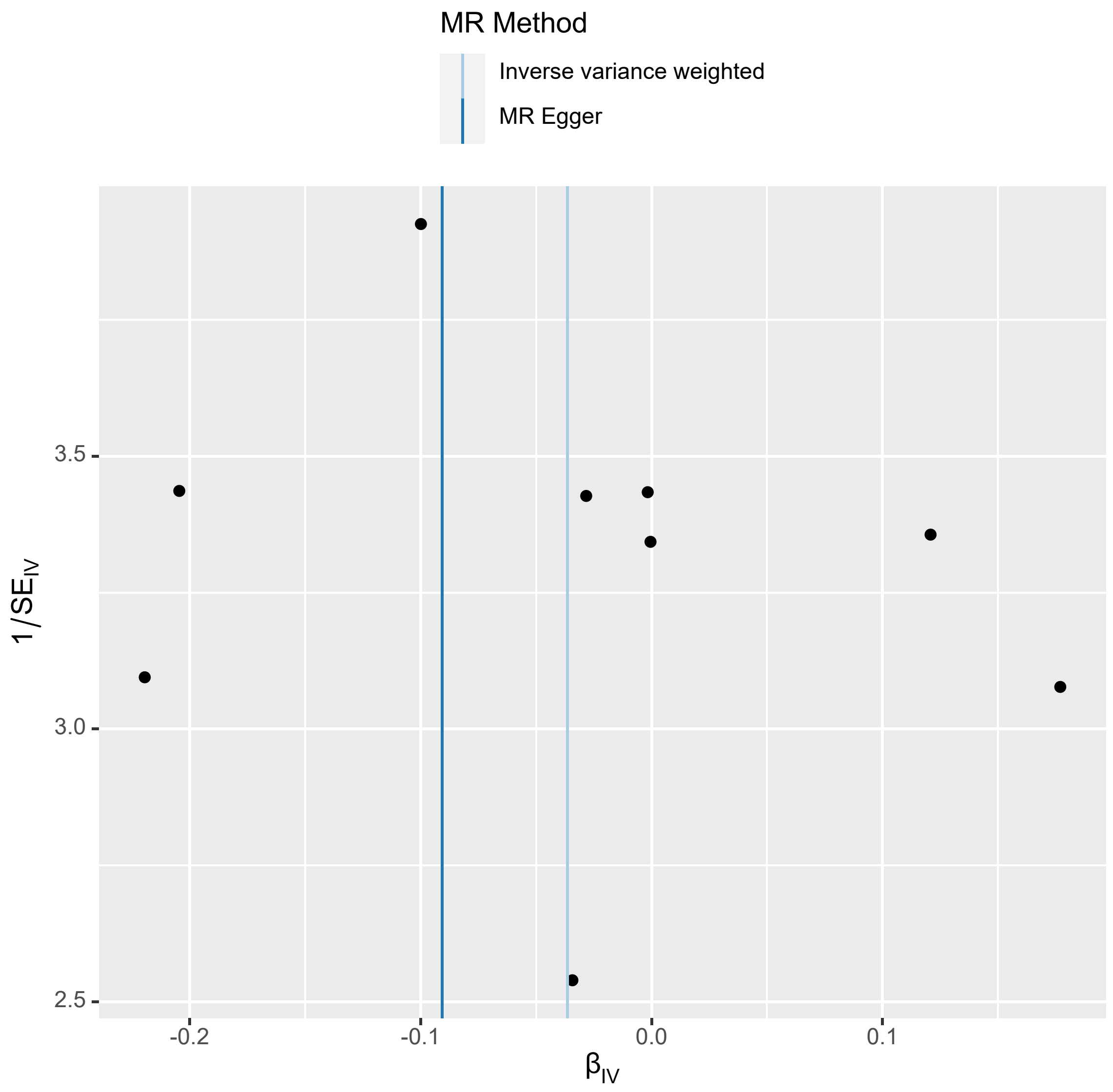

Figure S60. The funnel plot of family Porphyromonadaceae on prostate cancer in the FinnGen consortium

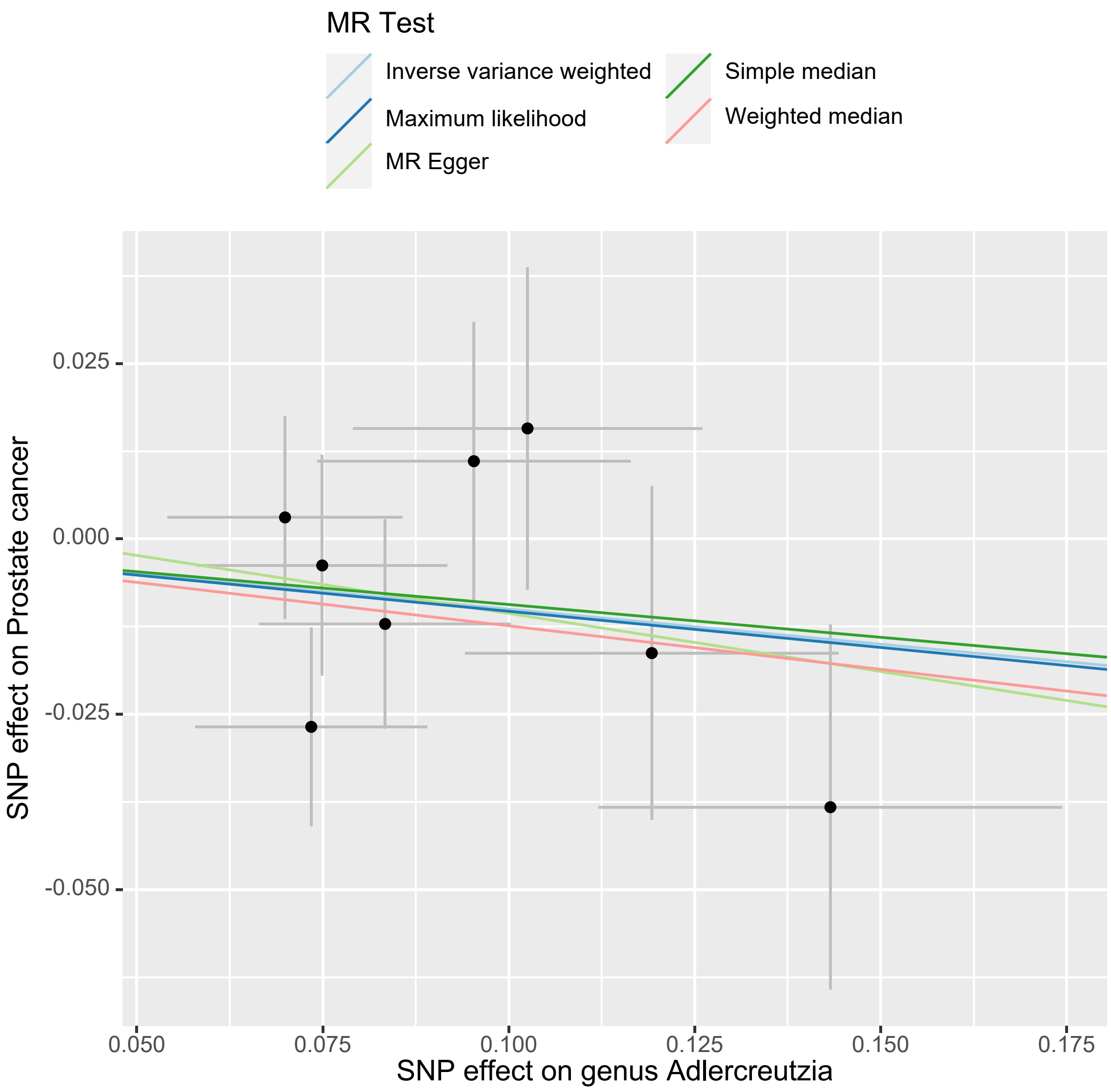

Figure S61. The scatter plot of genus Adlercreutzia on prostate cancer in the FinnGen consortium

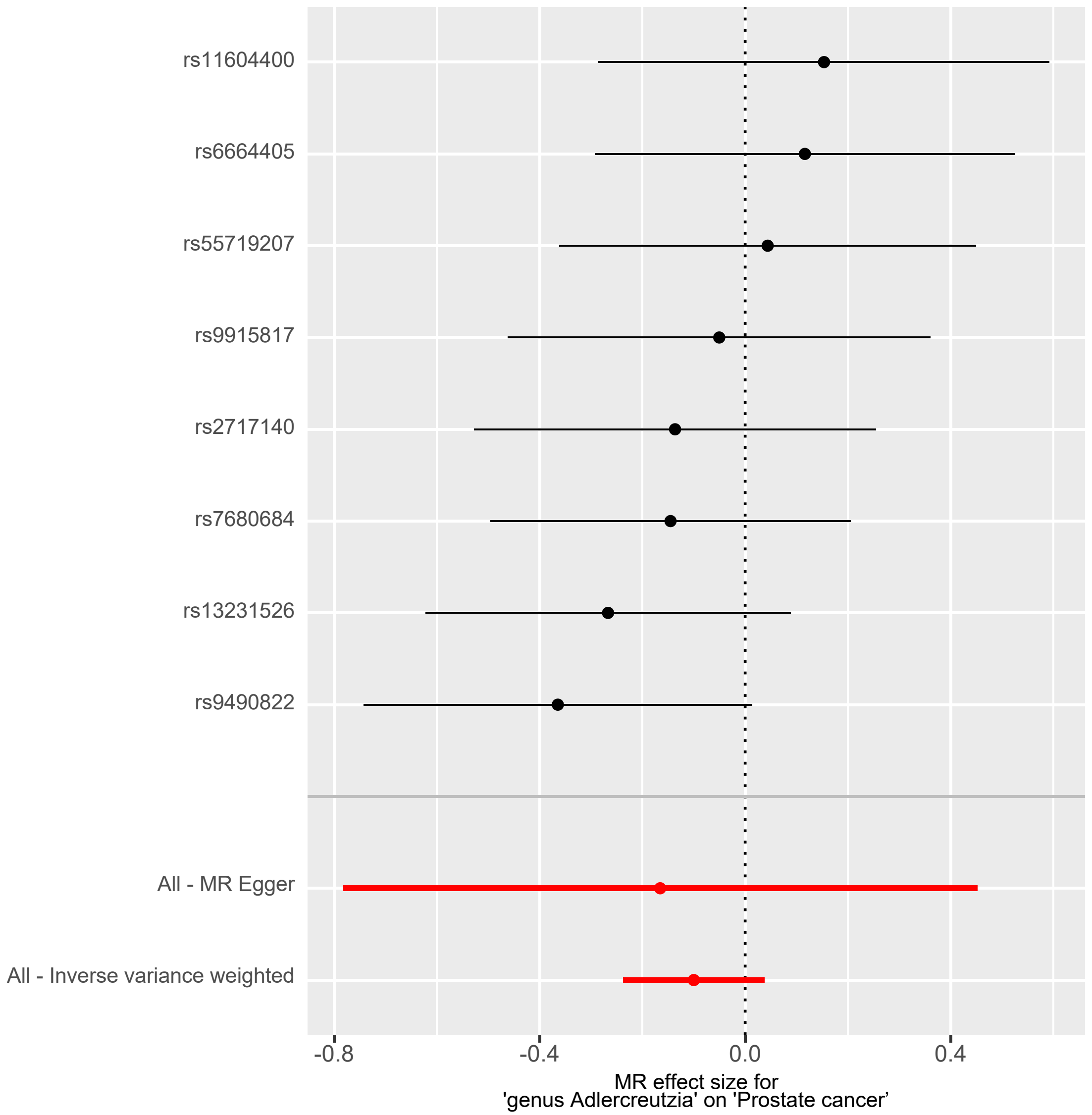

Figure S62. The forest plot of genus Adlercreutzia on prostate cancer in the FinnGen consortium

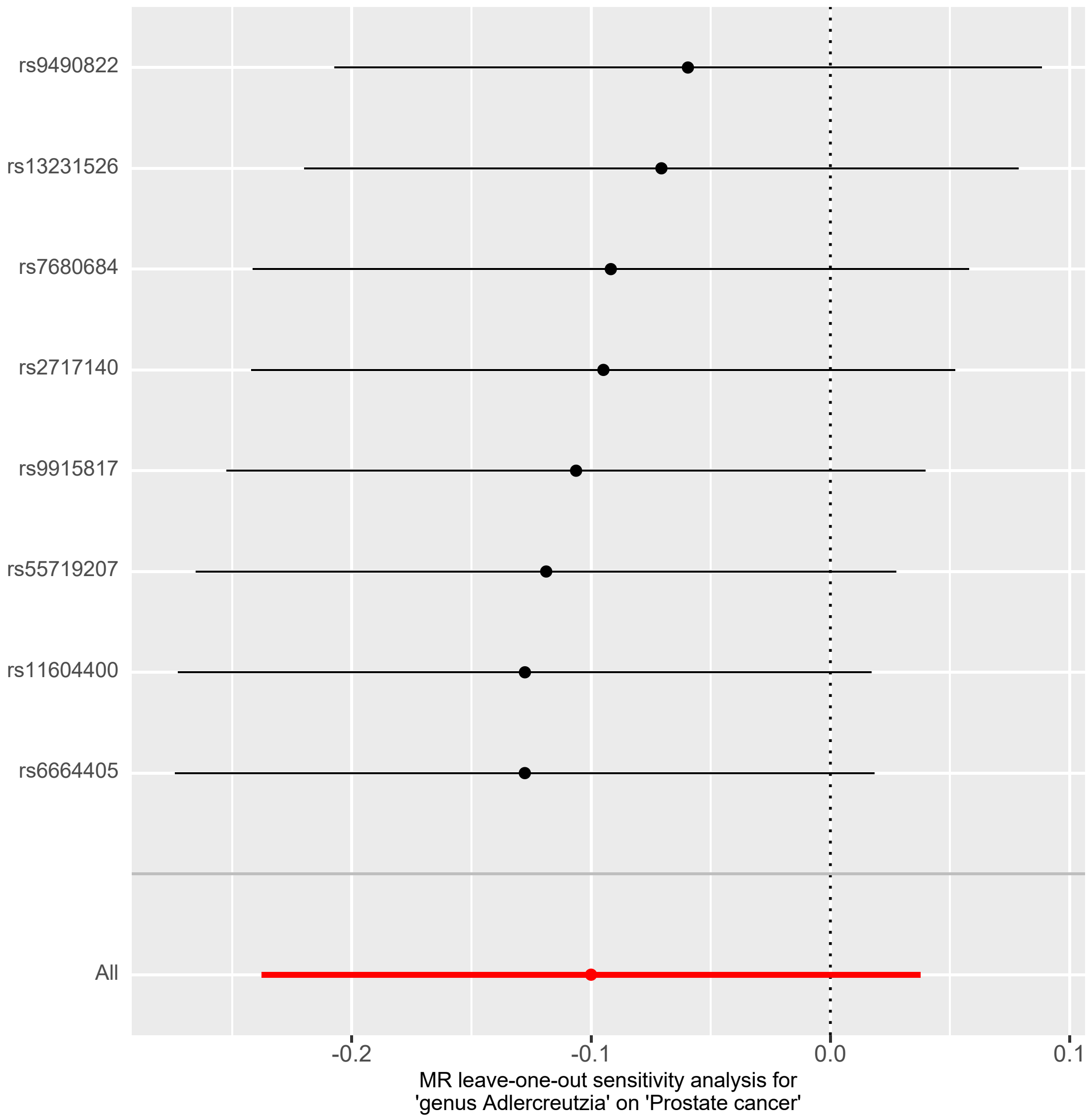

Figure S63. The leave-one-out test of genus Adlercreutzia on prostate cancer in the FinnGen consortium

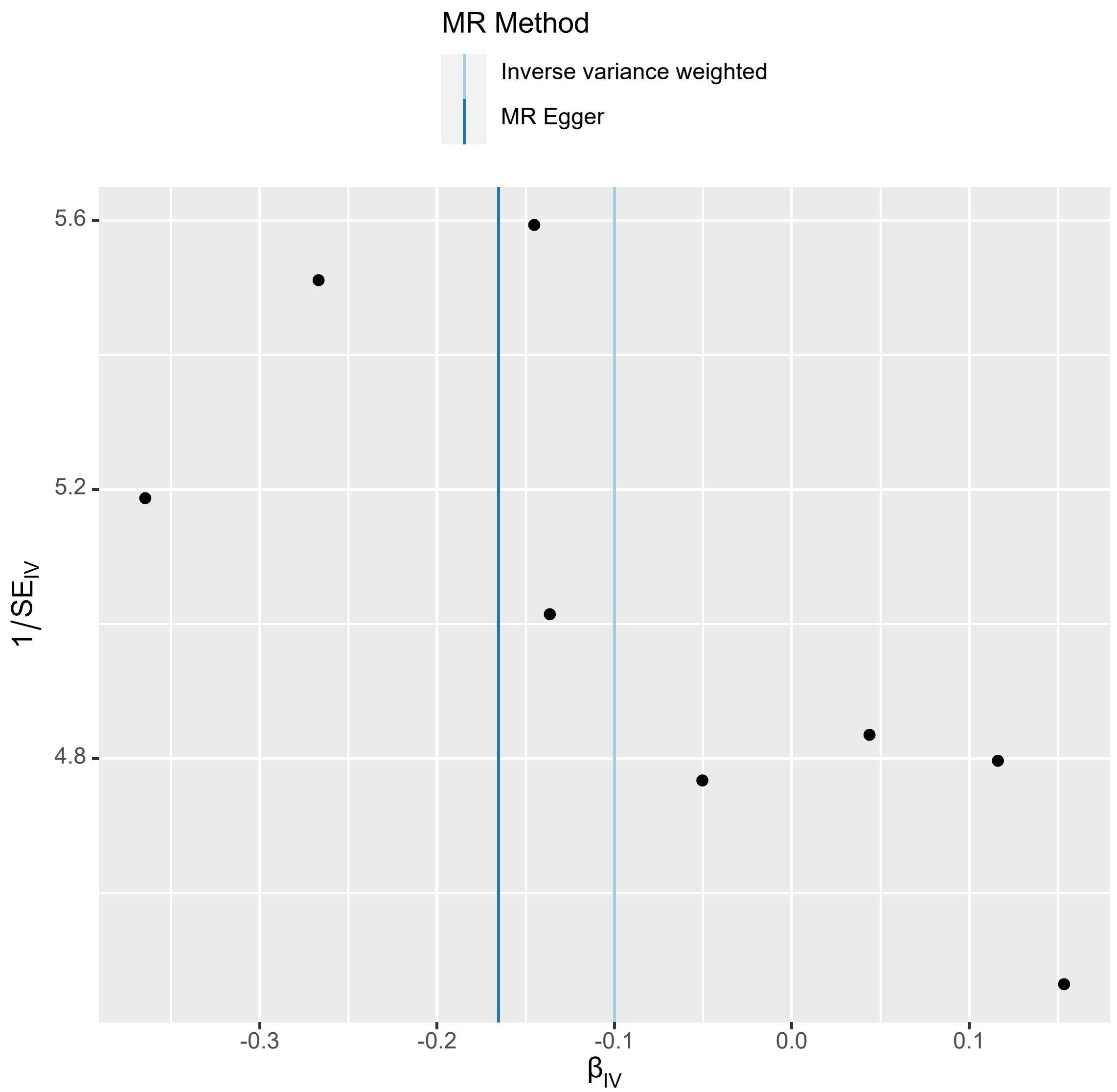

Figure S64. The funnel plot of genus Adlercreutzia on prostate cancer in the FinnGen consortium

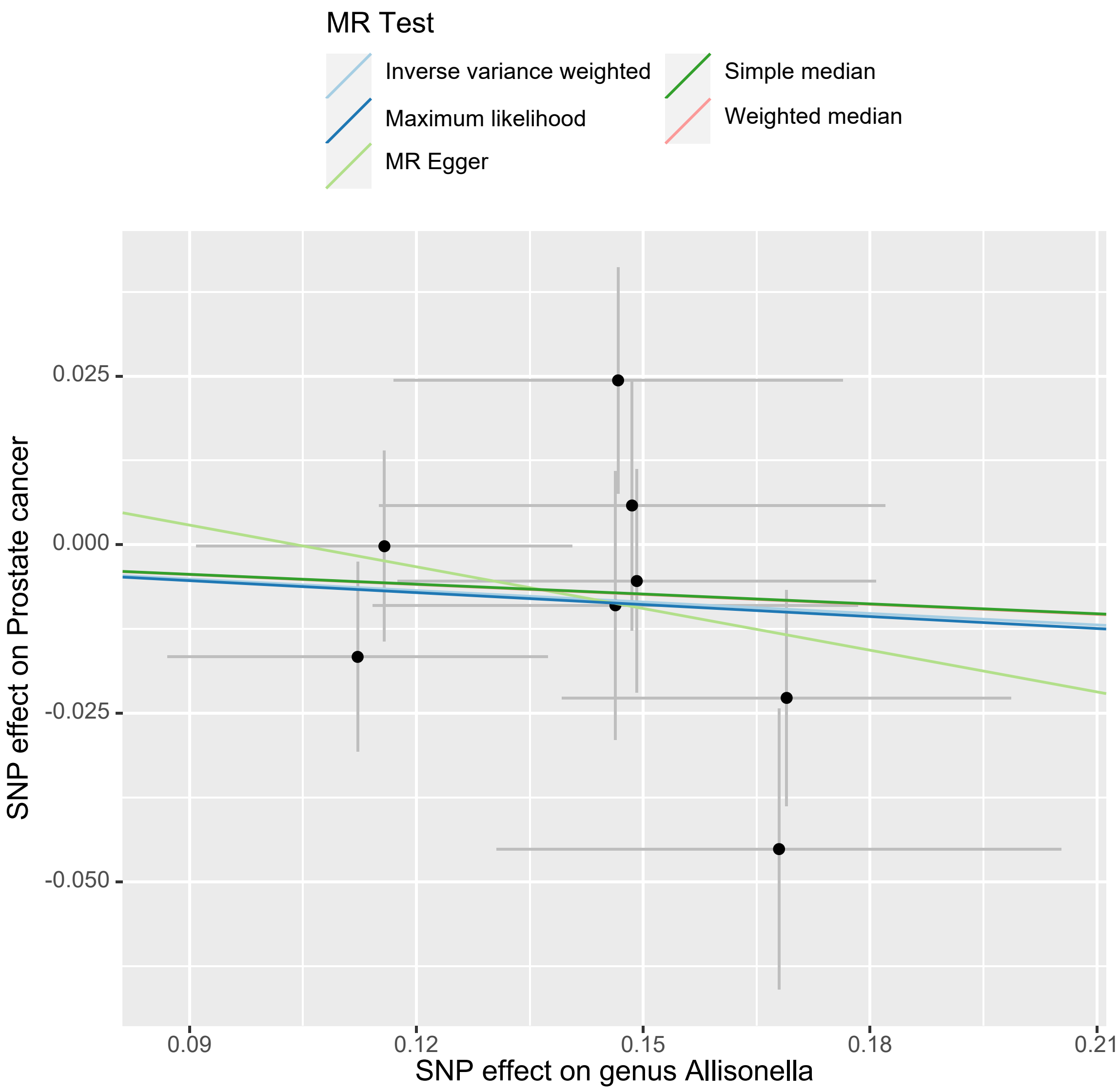

Figure S65. The scatter plot of genus Allisonella on prostate cancer in the FinnGen consortium

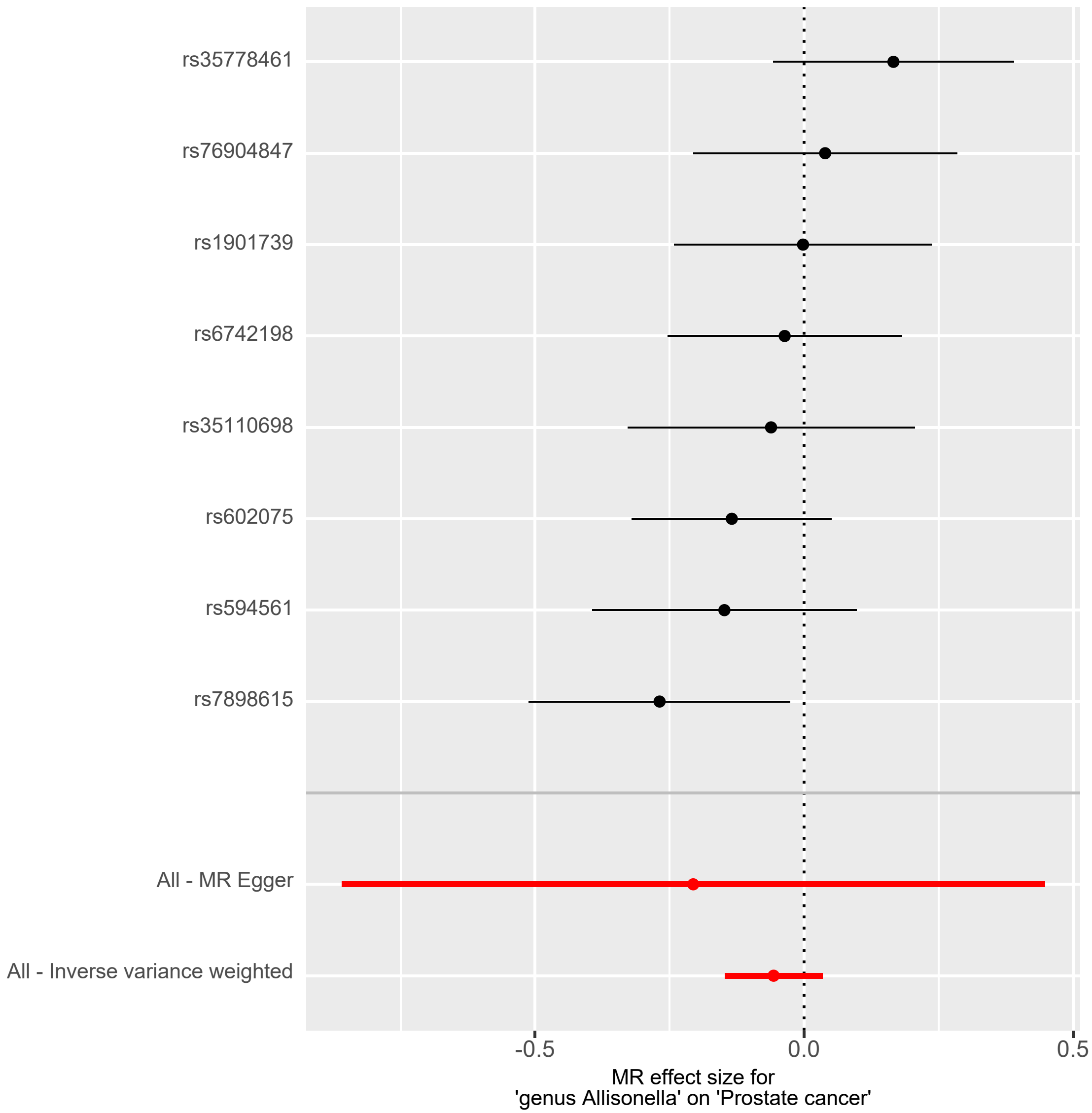

Figure S66. The forest plot of genus Allisonella on prostate cancer in the FinnGen consortium

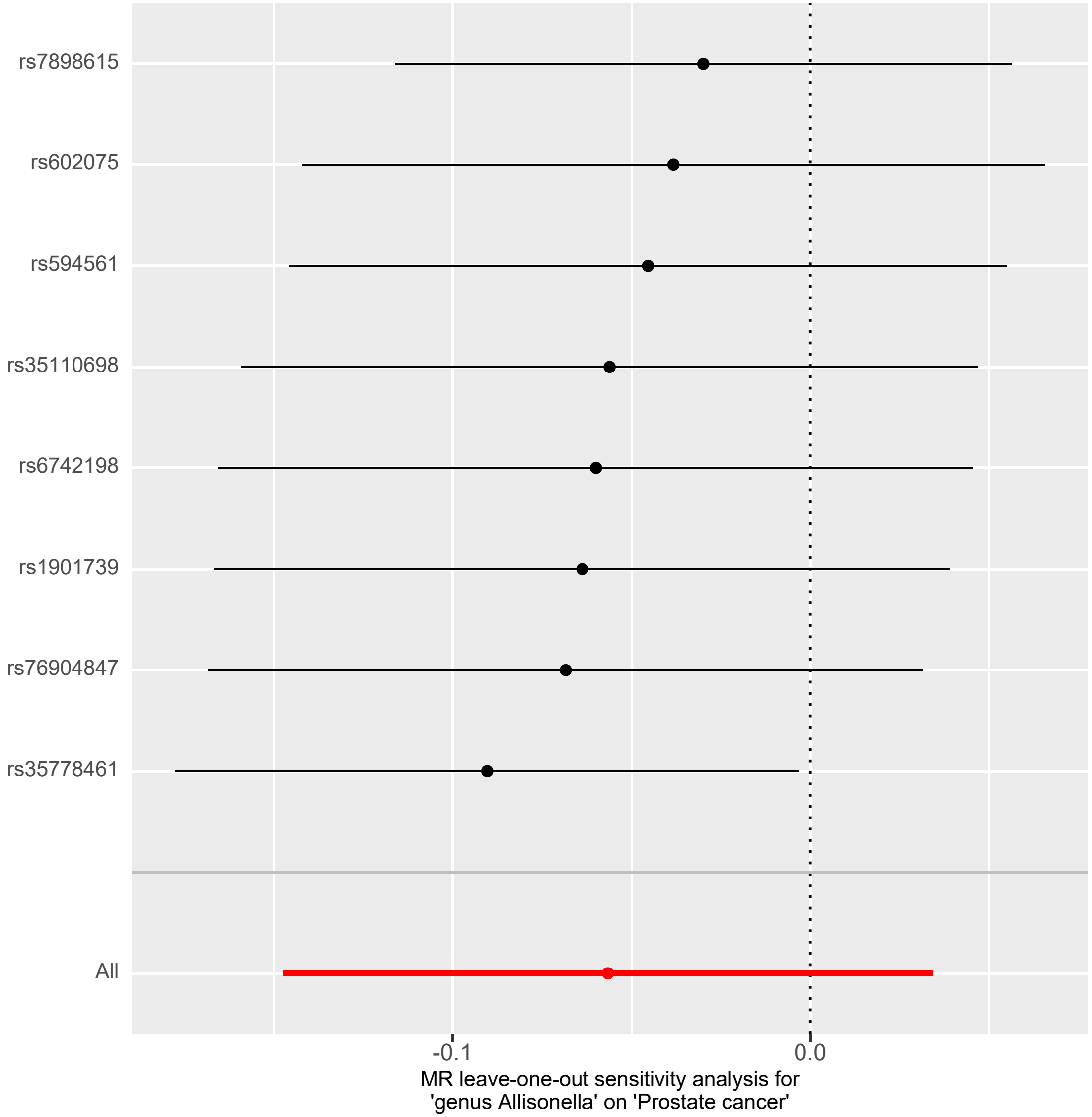

Figure S67. The leave-one-out test of genus Allisonella on prostate cancer in the FinnGen consortium

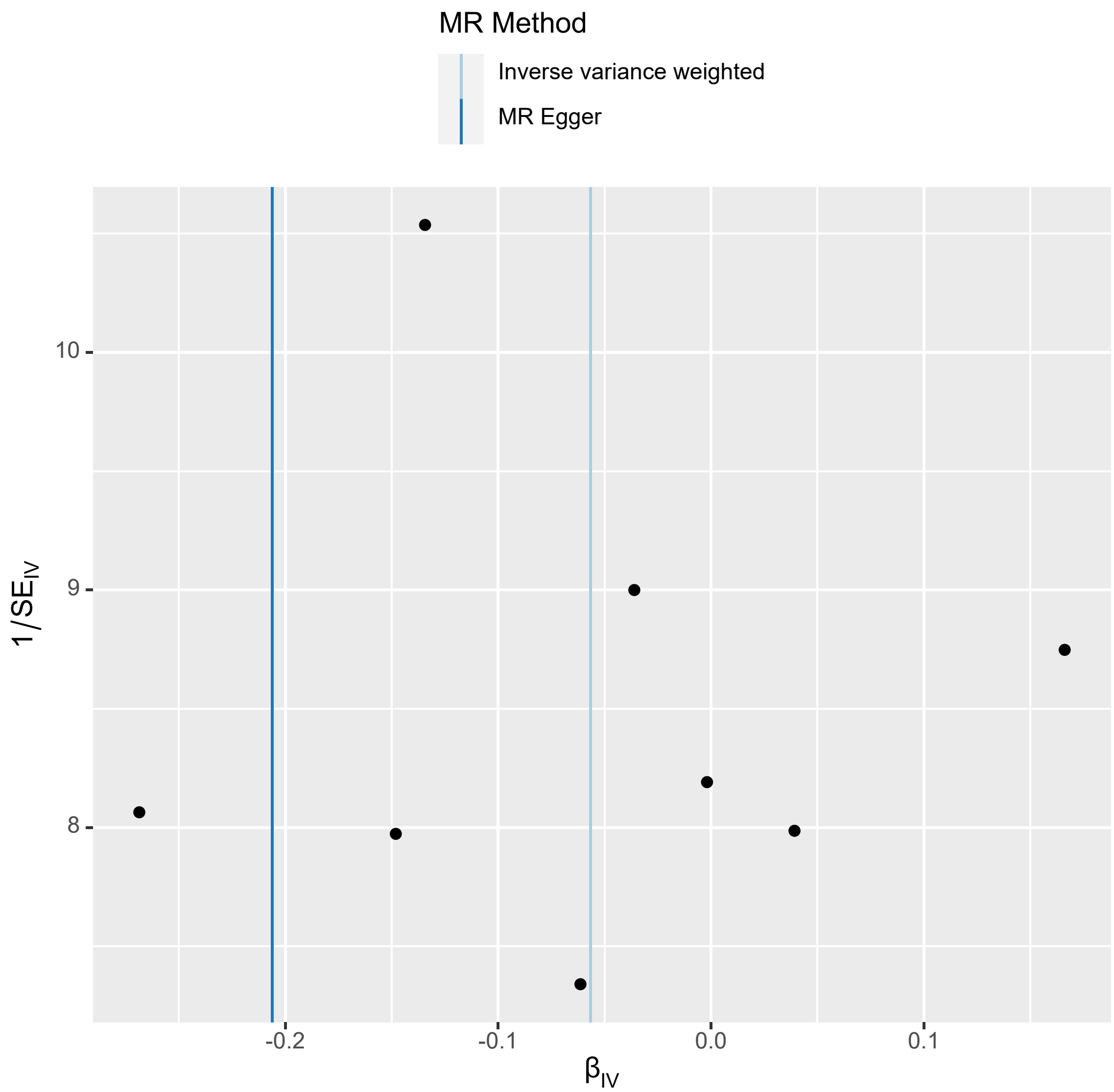

Figure S68. The funnel plot of genus Allisonella on prostate cancer in the FinnGen consortium

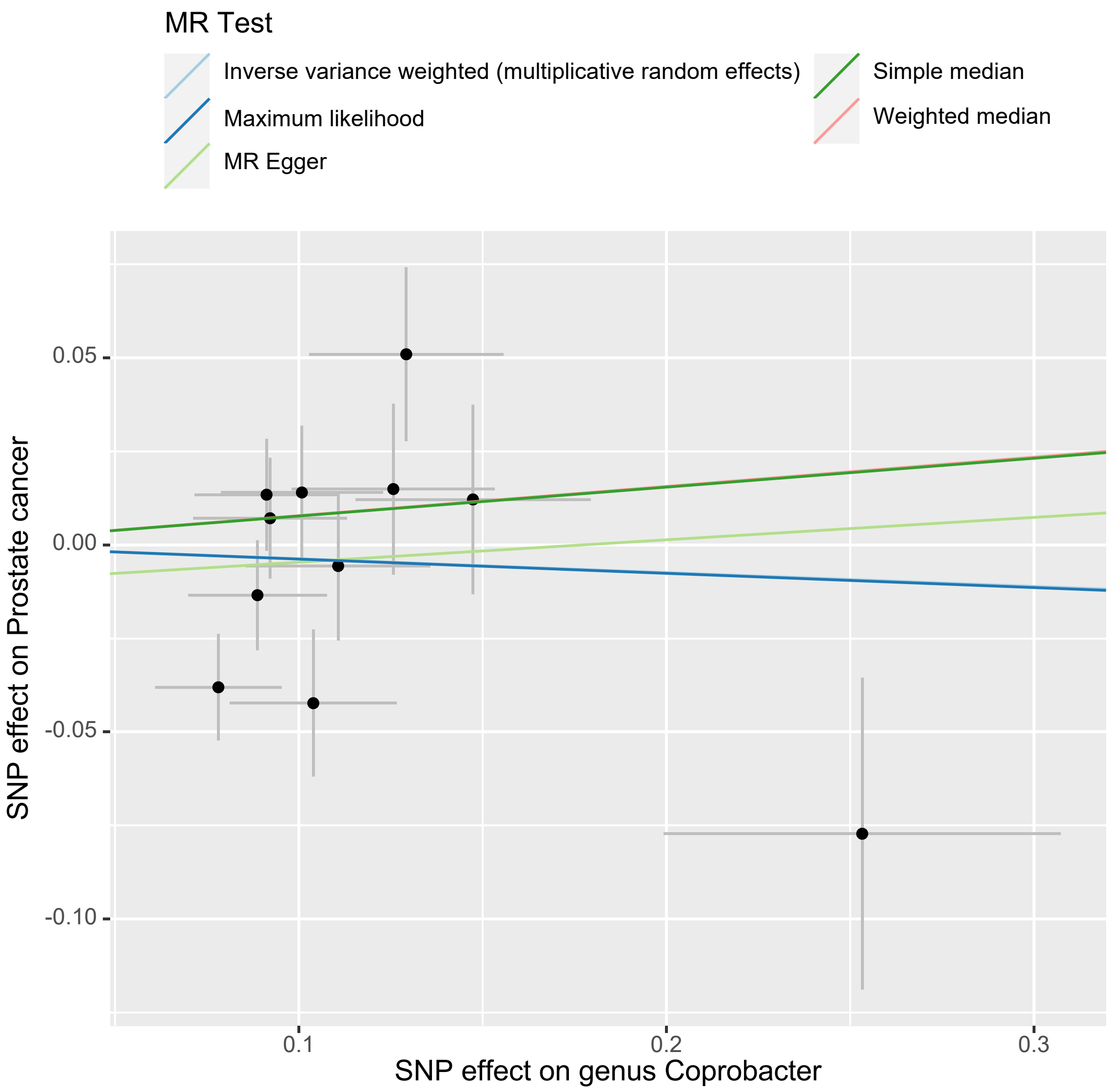

Figure S69. The scatter plot of genus Coprobacter on prostate cancer in the FinnGen consortium

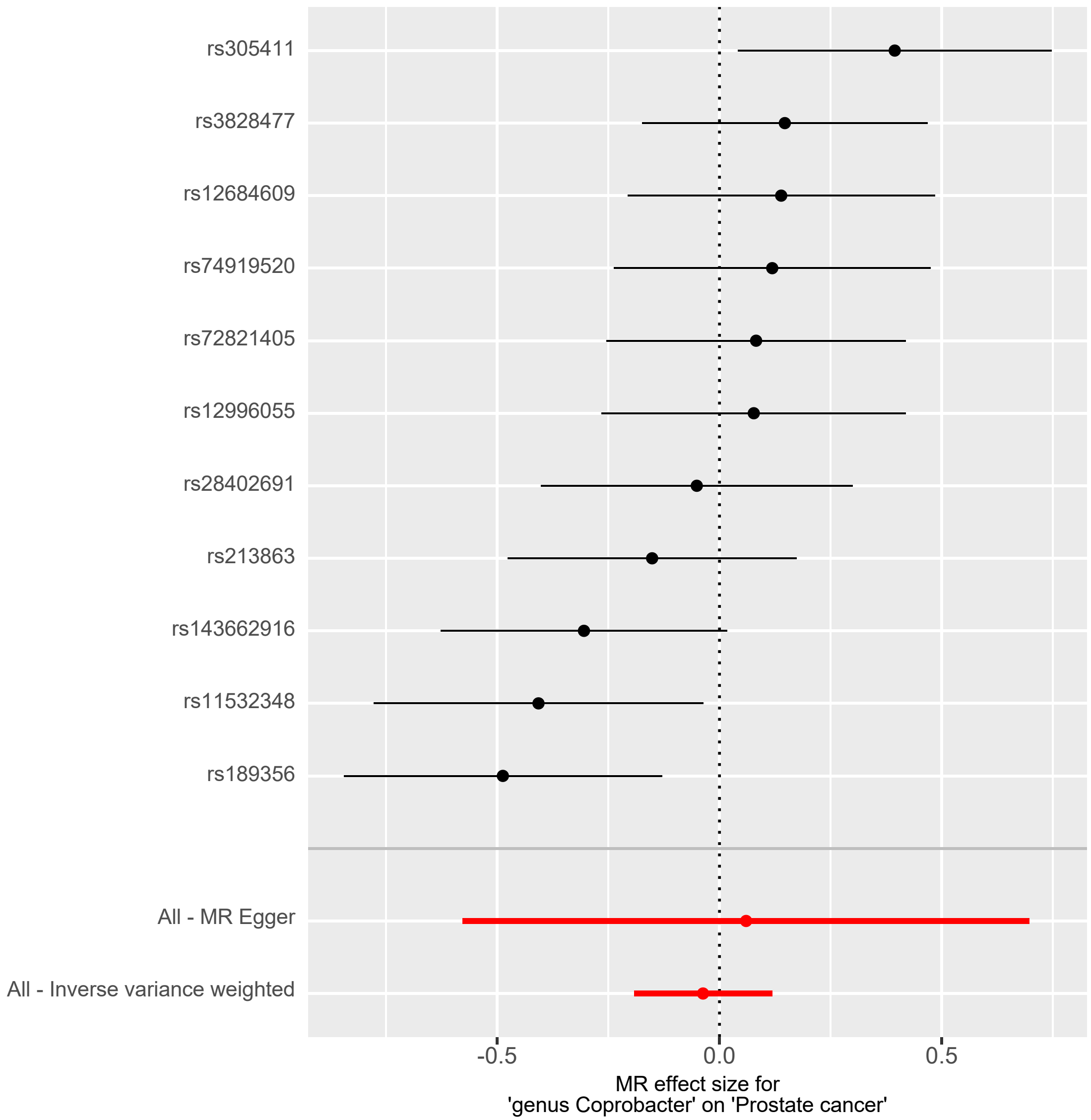

Figure S70. The forest plot of genus Coprobacter on prostate cancer in the FinnGen consortium

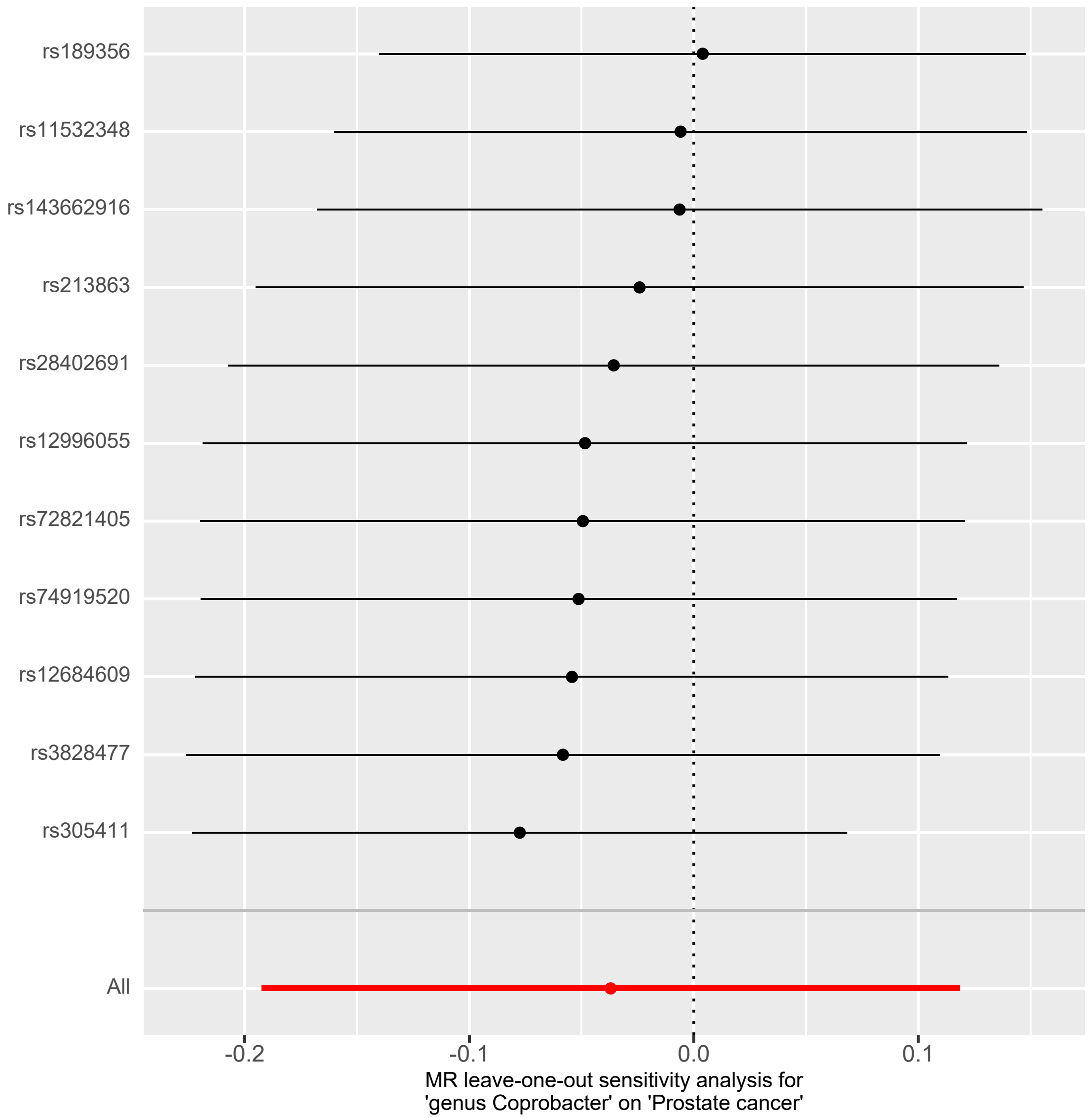

Figure S71. The leave-one-out test of genus Coprobacter on prostate cancer in the FinnGen consortium

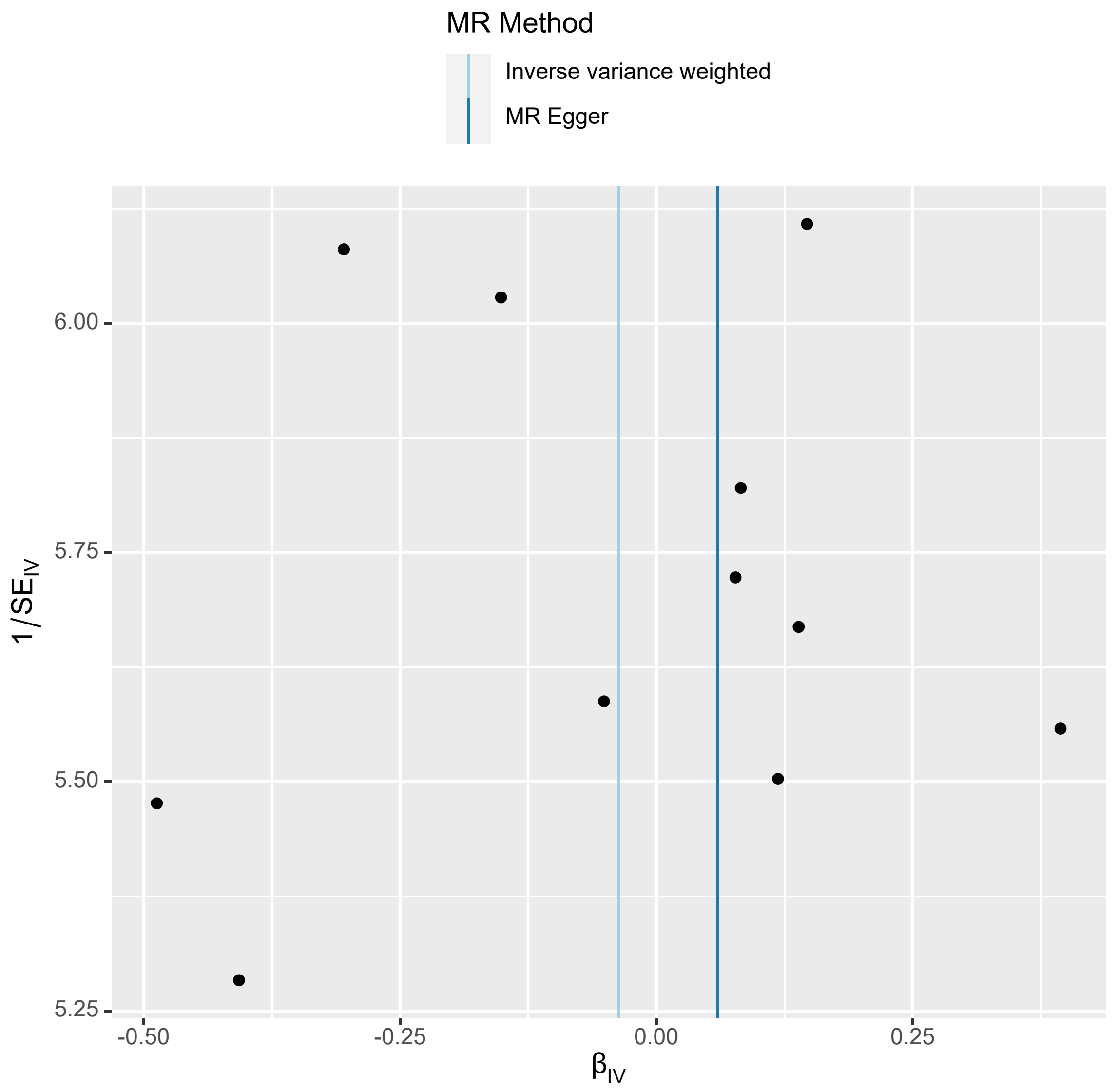

Figure S72. The funnel plot of genus Coprobacter on prostate cancer in the FinnGen consortium

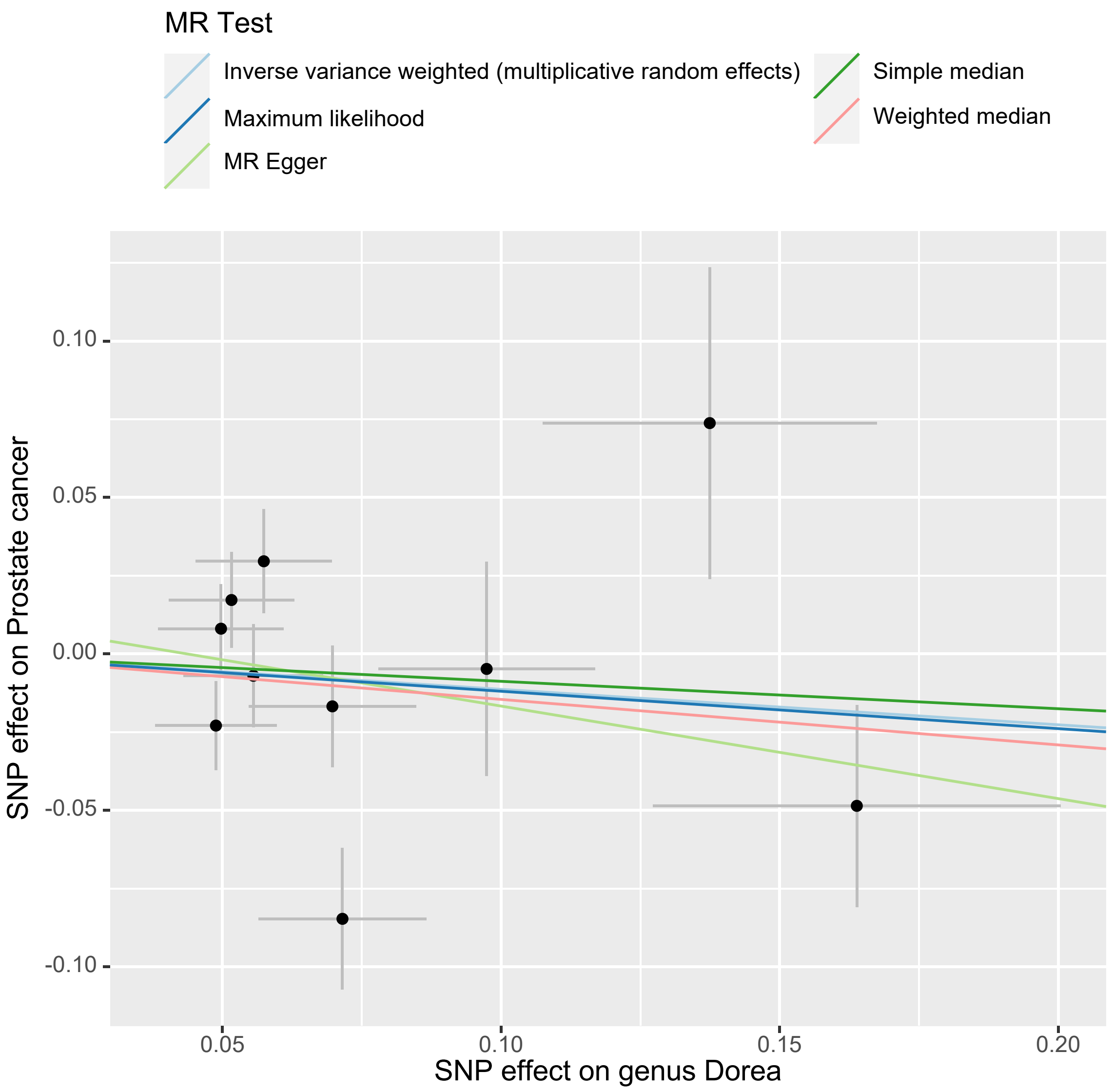

Figure S73. The scatter plot of genus Dorea on prostate cancer in the FinnGen consortium

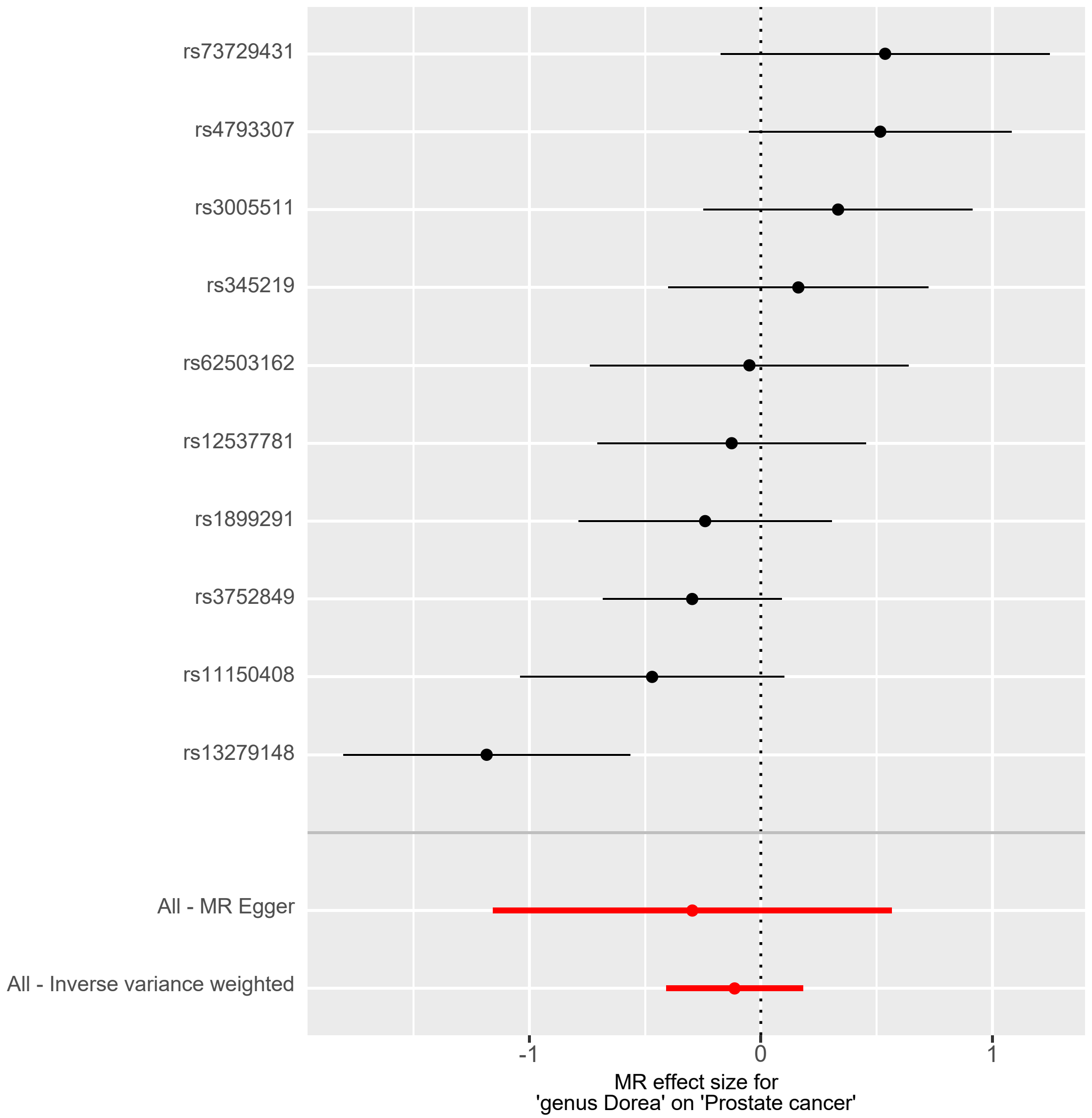

Figure S74. The forest plot of genus Dorea on prostate cancer in the FinnGen consortium

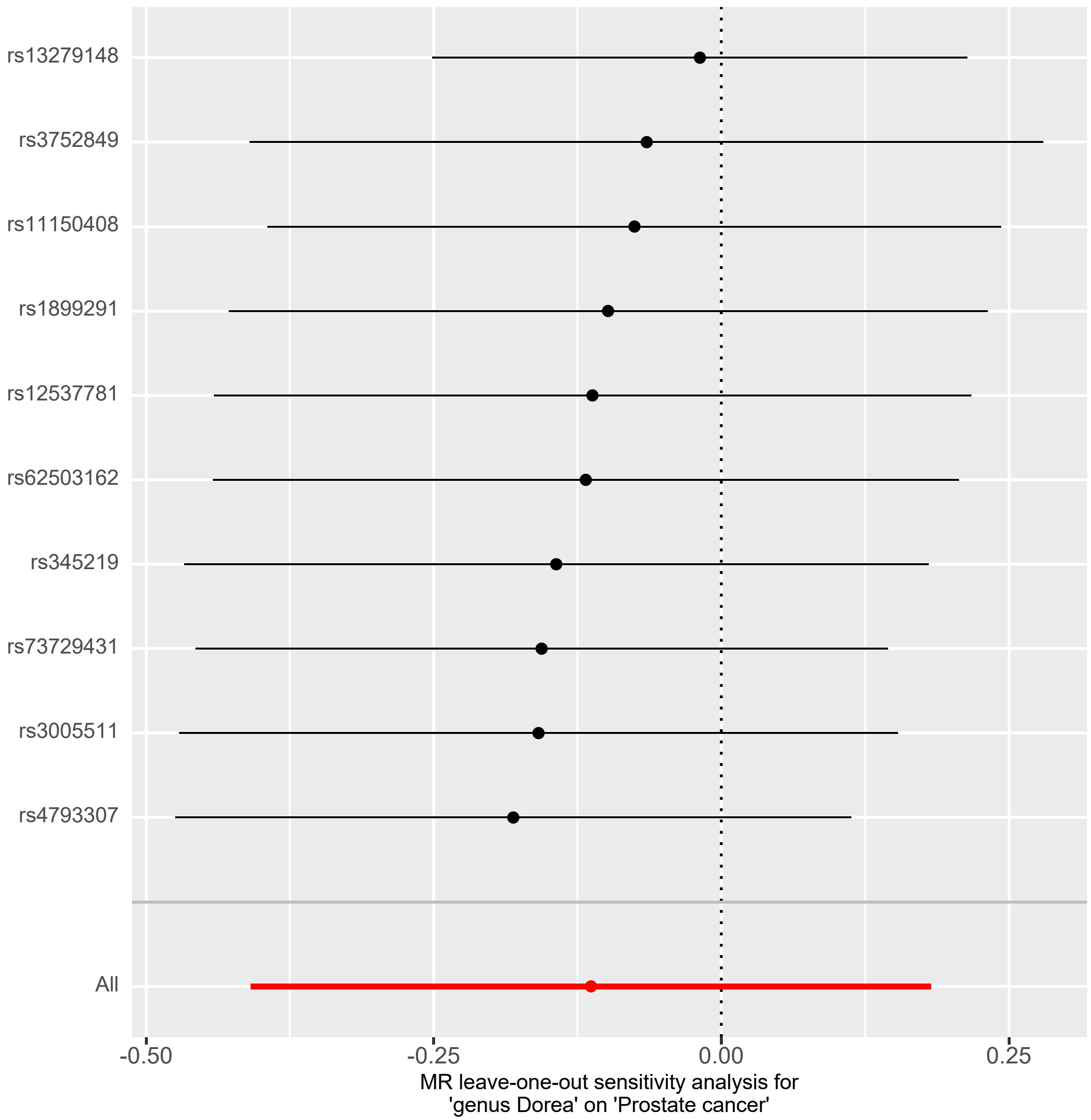

Figure S75. The leave-one-out test of genus Dorea on prostate cancer in the FinnGen consortium

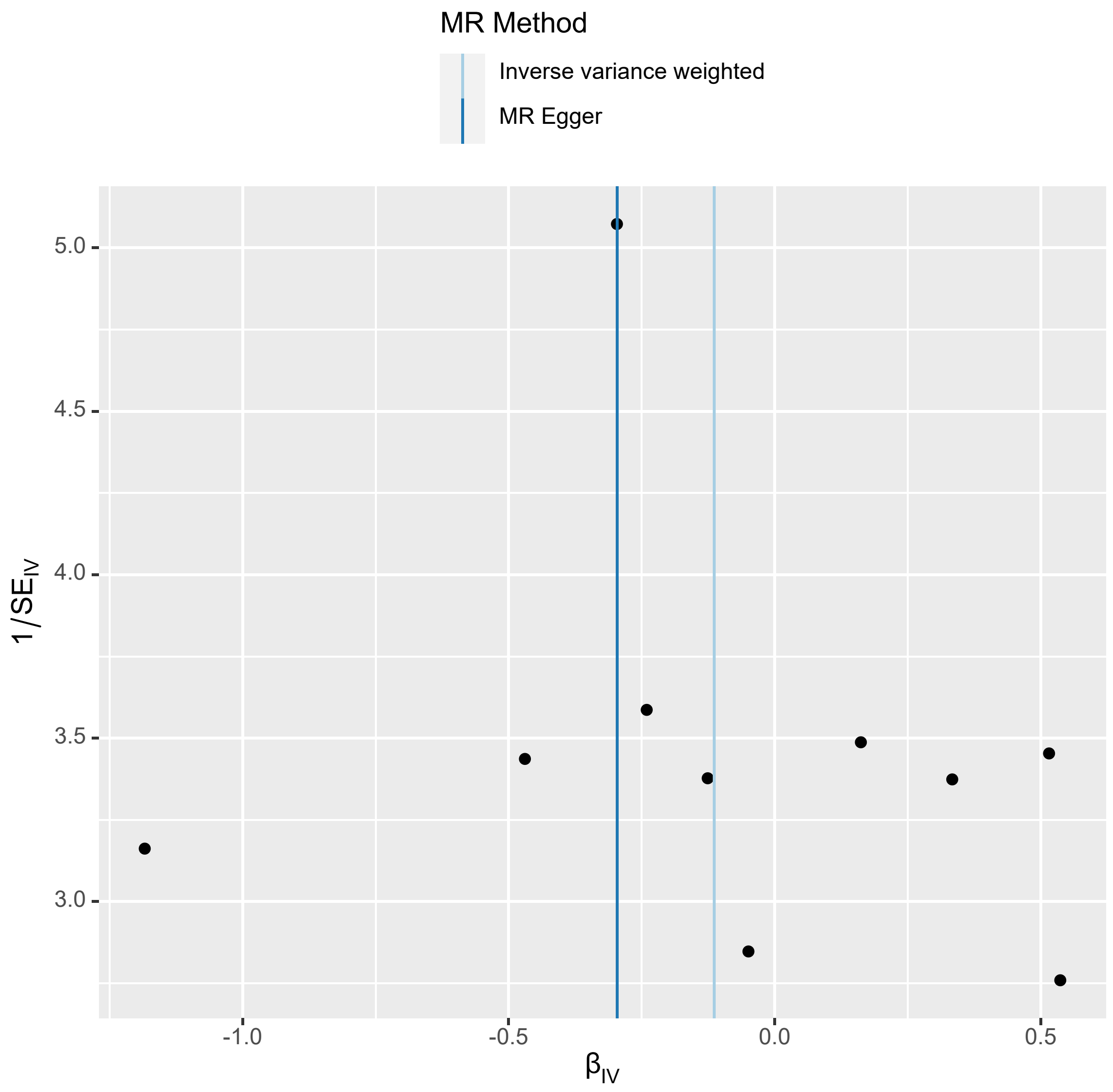

Figure S76. The funnel plot of genus Dorea on prostate cancer in the FinnGen consortium

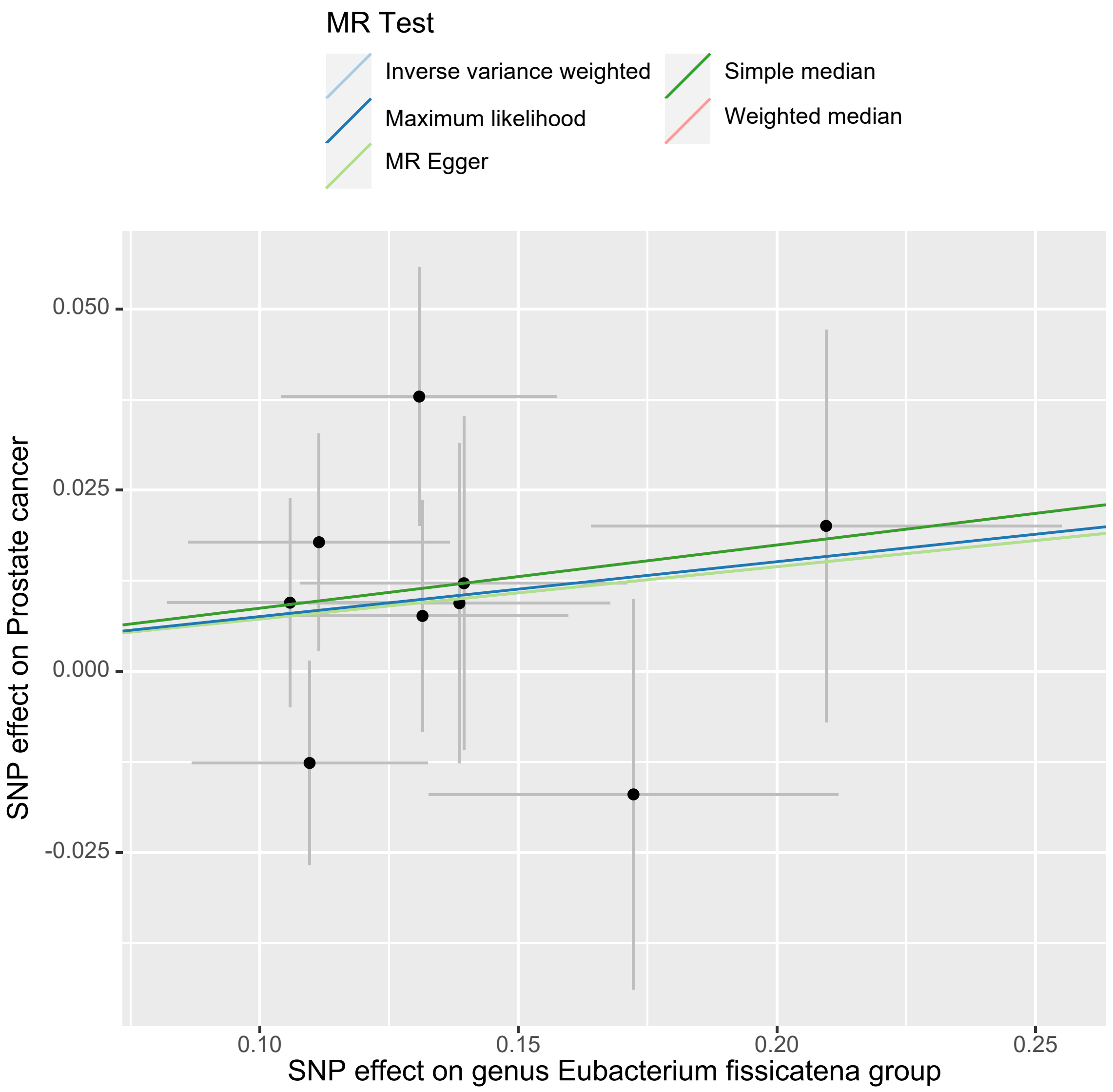

Figure S77. The scatter plot of genus Eubacterium fissicatena group on prostate cancer in the FinnGen consortium

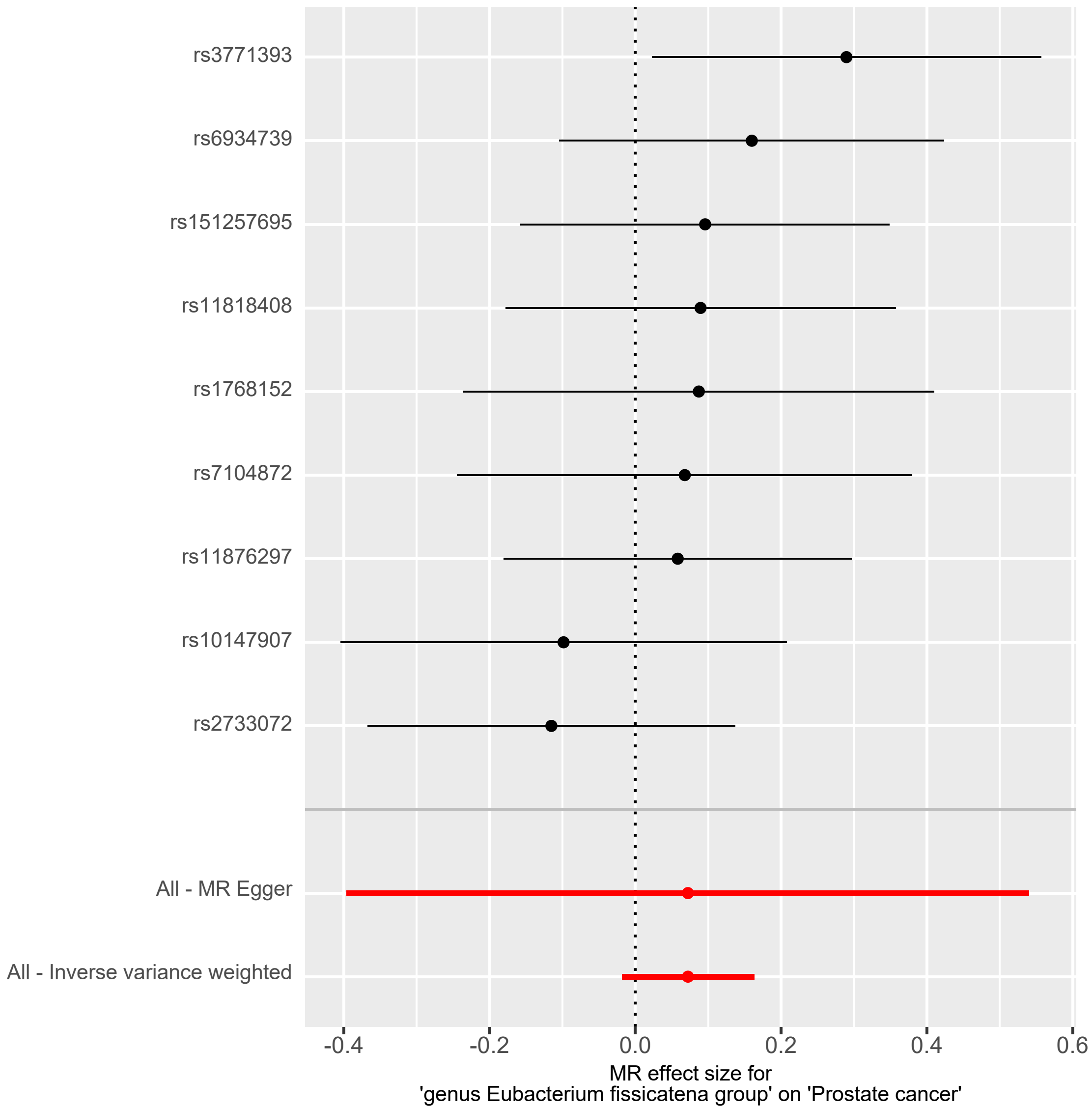

Figure S78. The forest plot of genus Eubacterium fissicatena group on prostate cancer in the FinnGen consortium

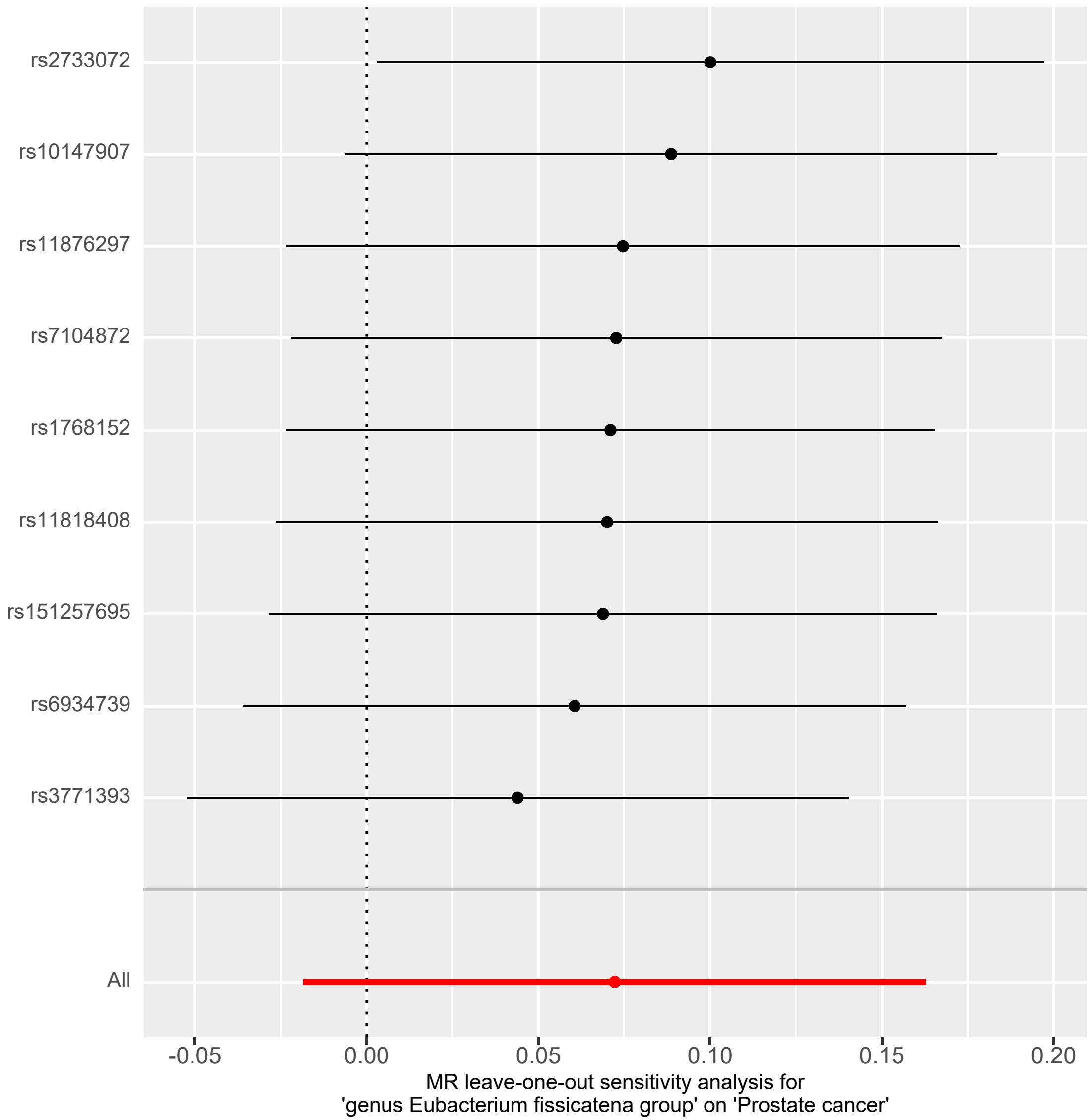

Figure S79. The leave-one-out test of genus Eubacterium fissicatena group on prostate cancer in the FinnGen consortium

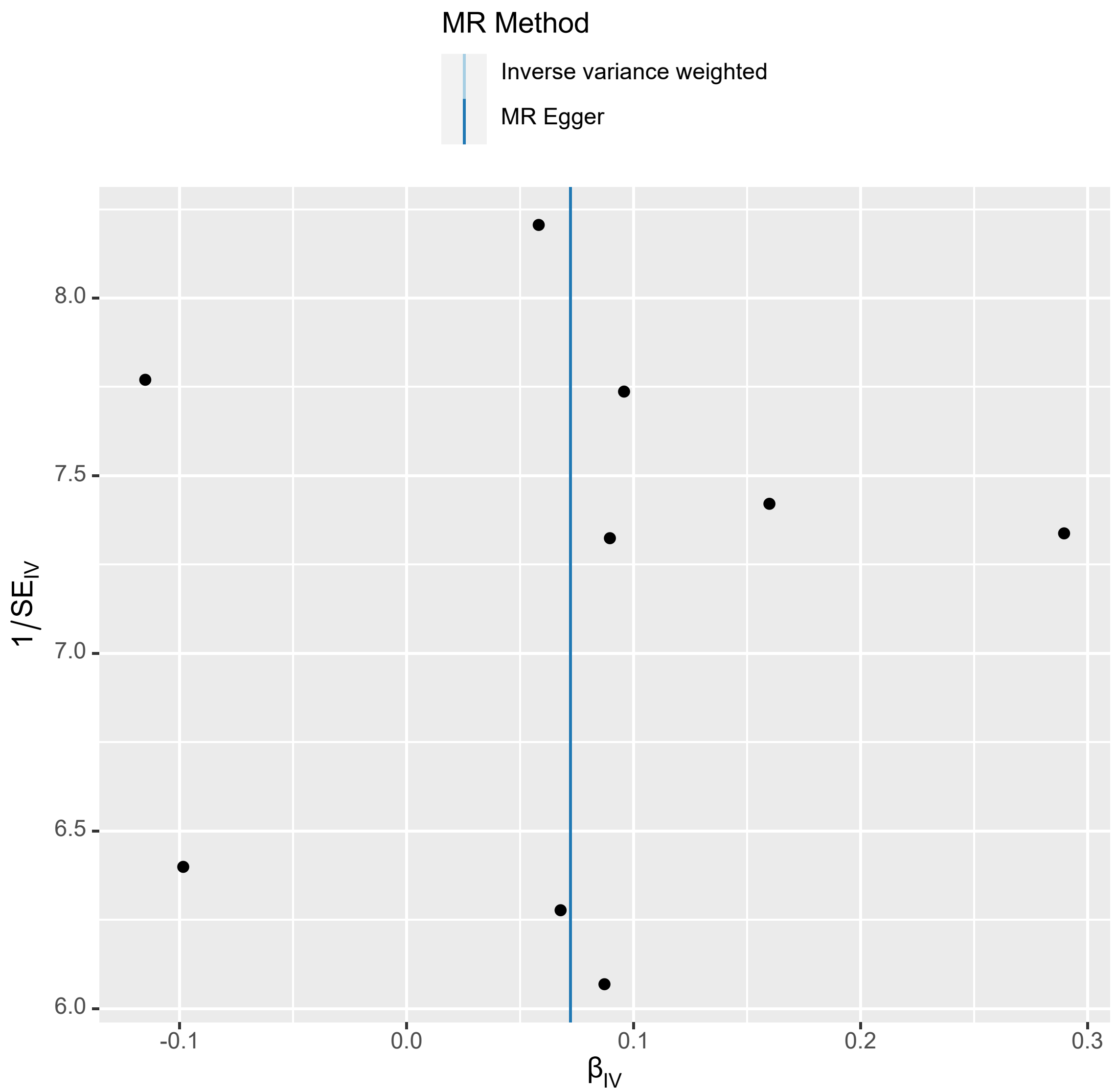

Figure S80. The funnel plot of genus Eubacterium fissicatena group on prostate cancer in the FinnGen consortium

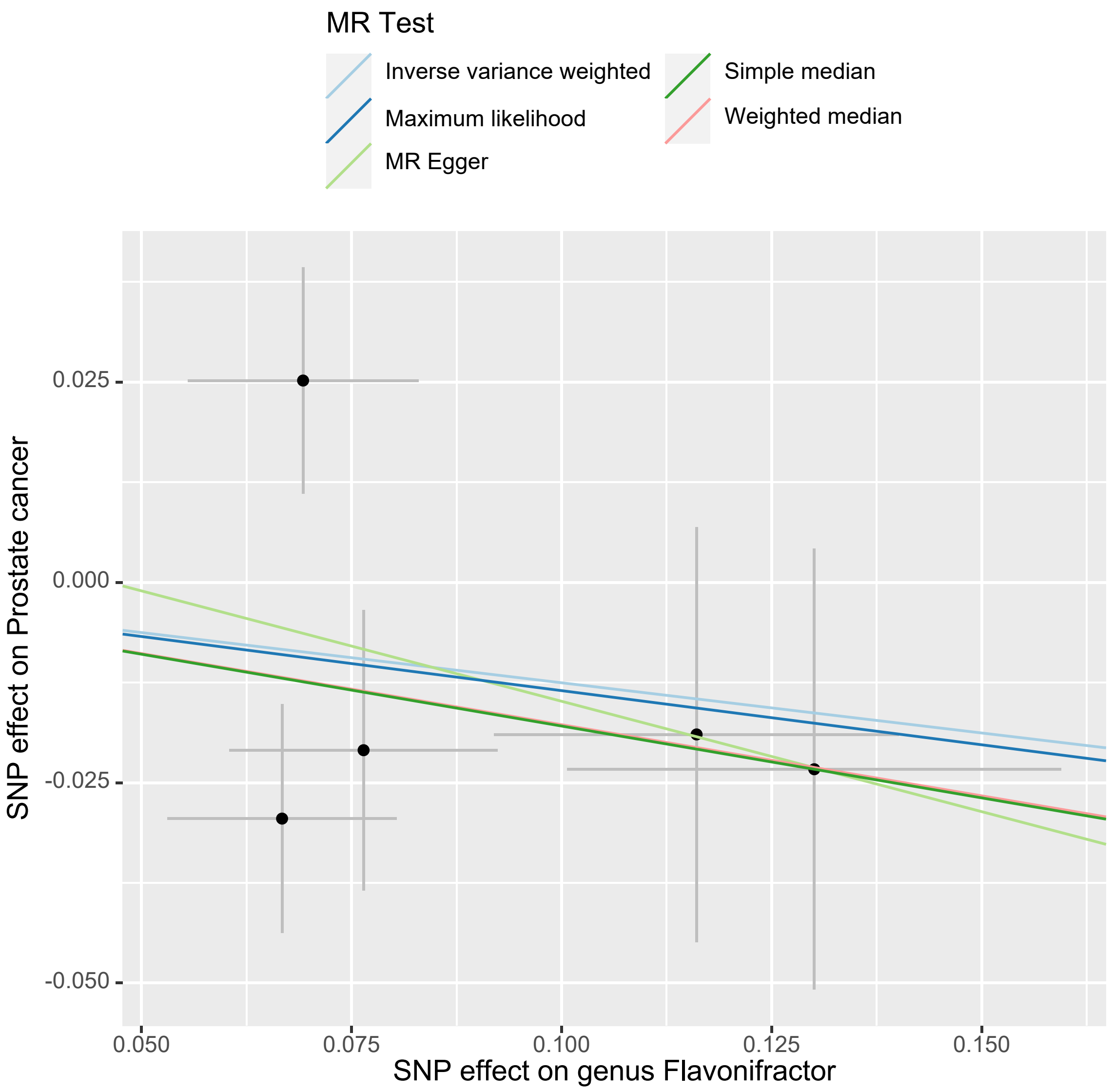

Figure S81. The scatter plot of genus Flavonifractor on prostate cancer in the FinnGen consortium

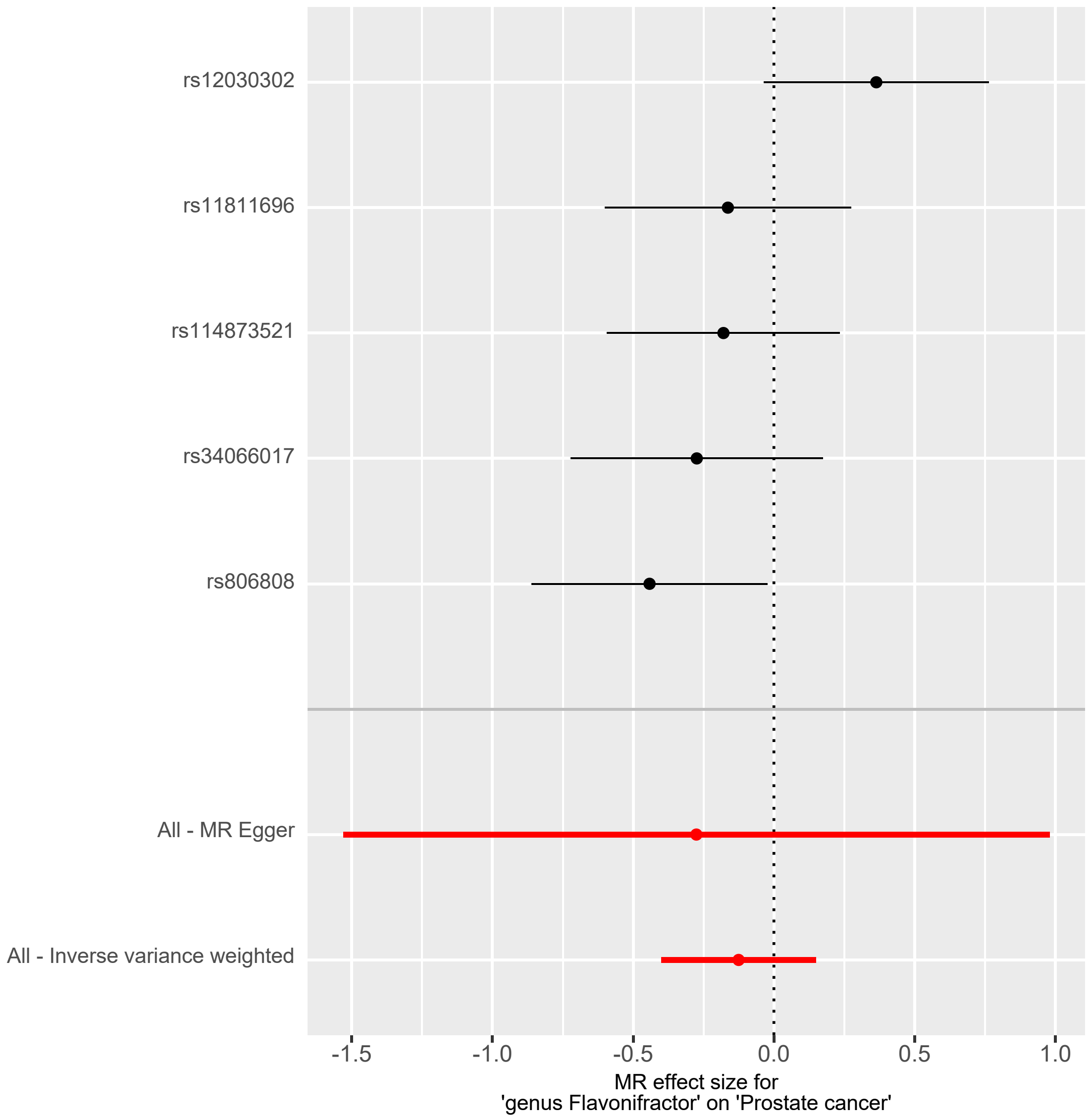

Figure S82. The forest plot of genus Flavonifractor on prostate cancer in the FinnGen consortium

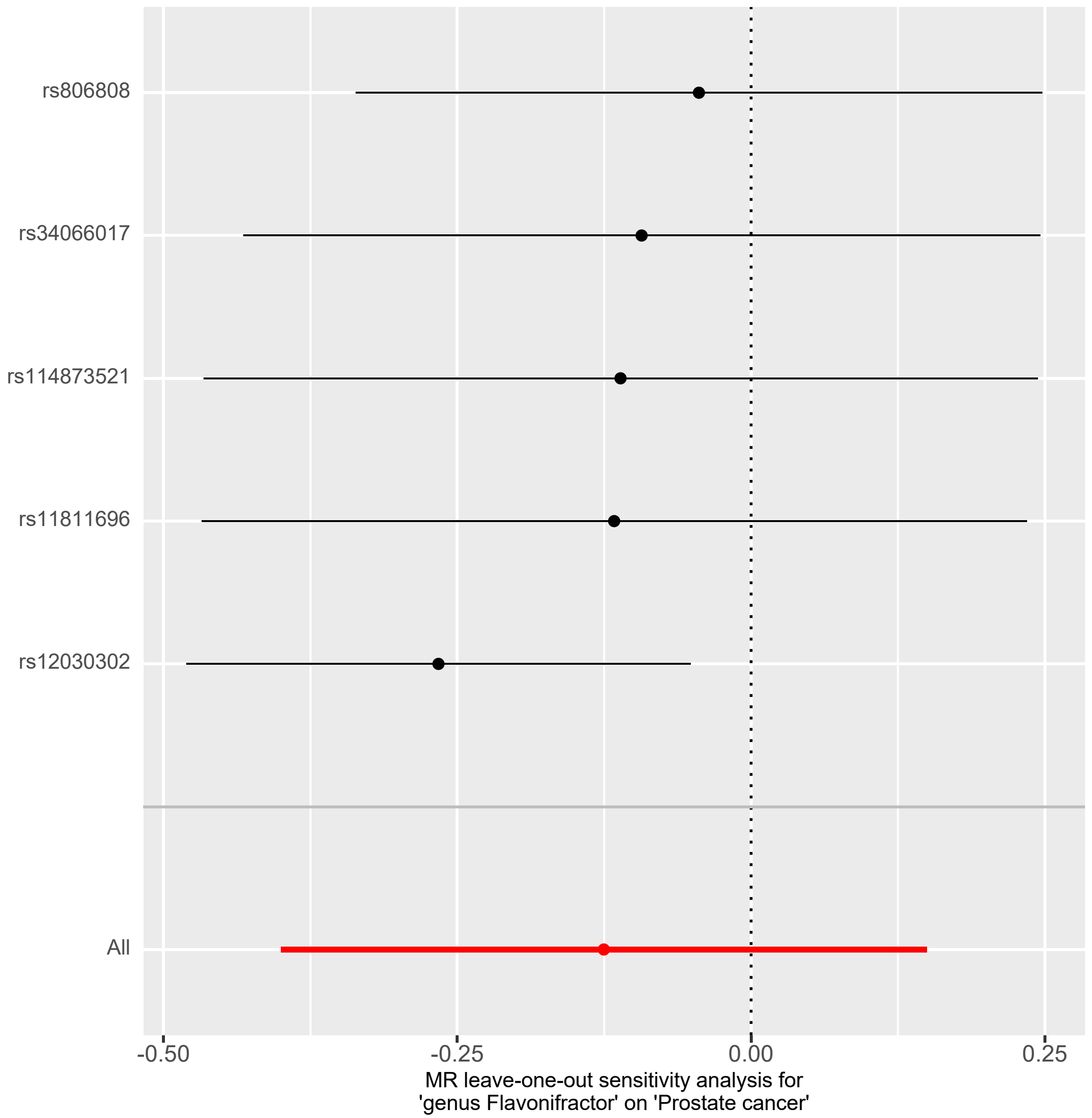

Figure S83. The leave-one-out test of genus Flavonifractor on prostate cancer in the FinnGen consortium

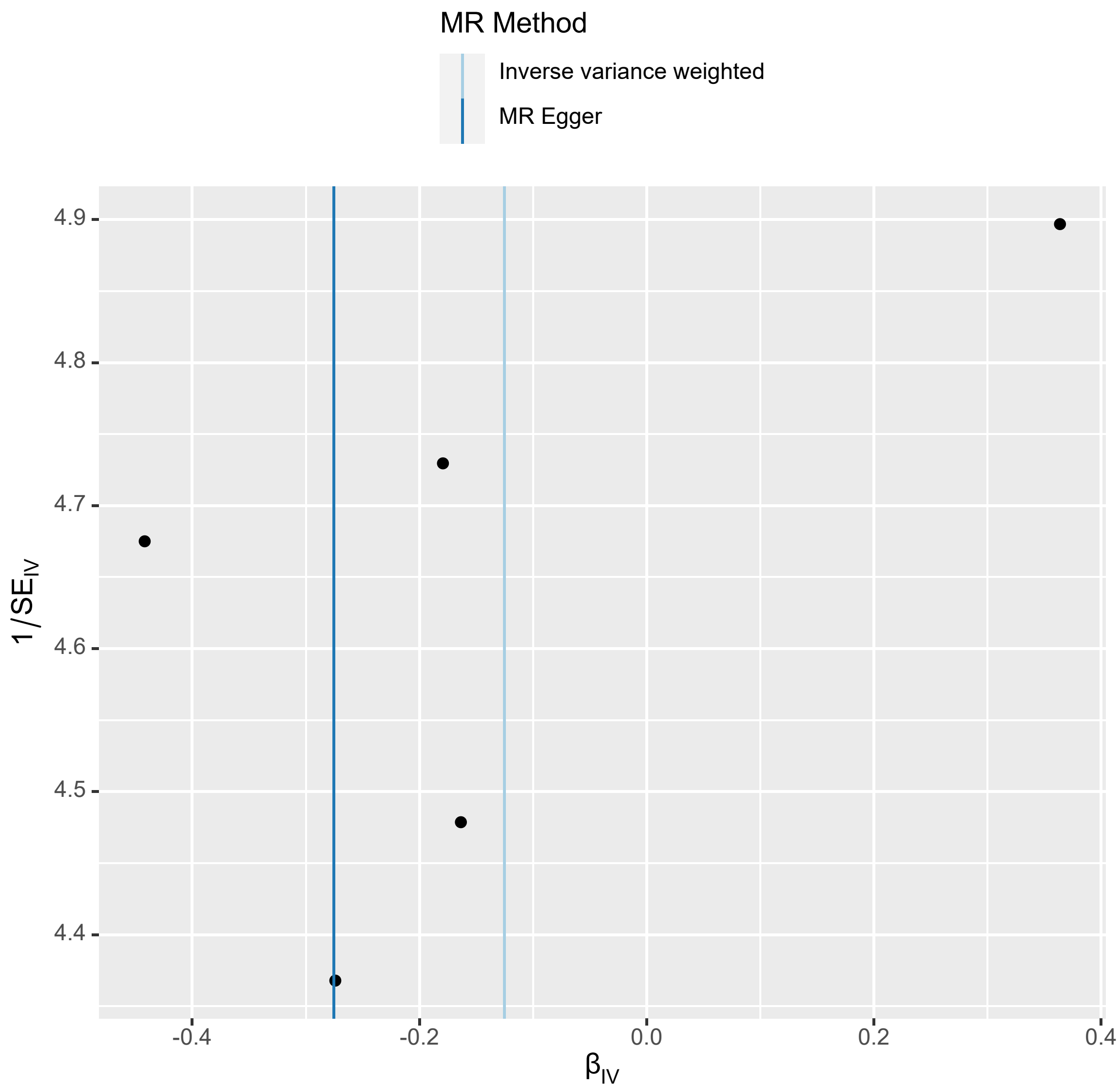

Figure S84. The funnel plot of genus Flavonifractor on prostate cancer in the FinnGen consortium

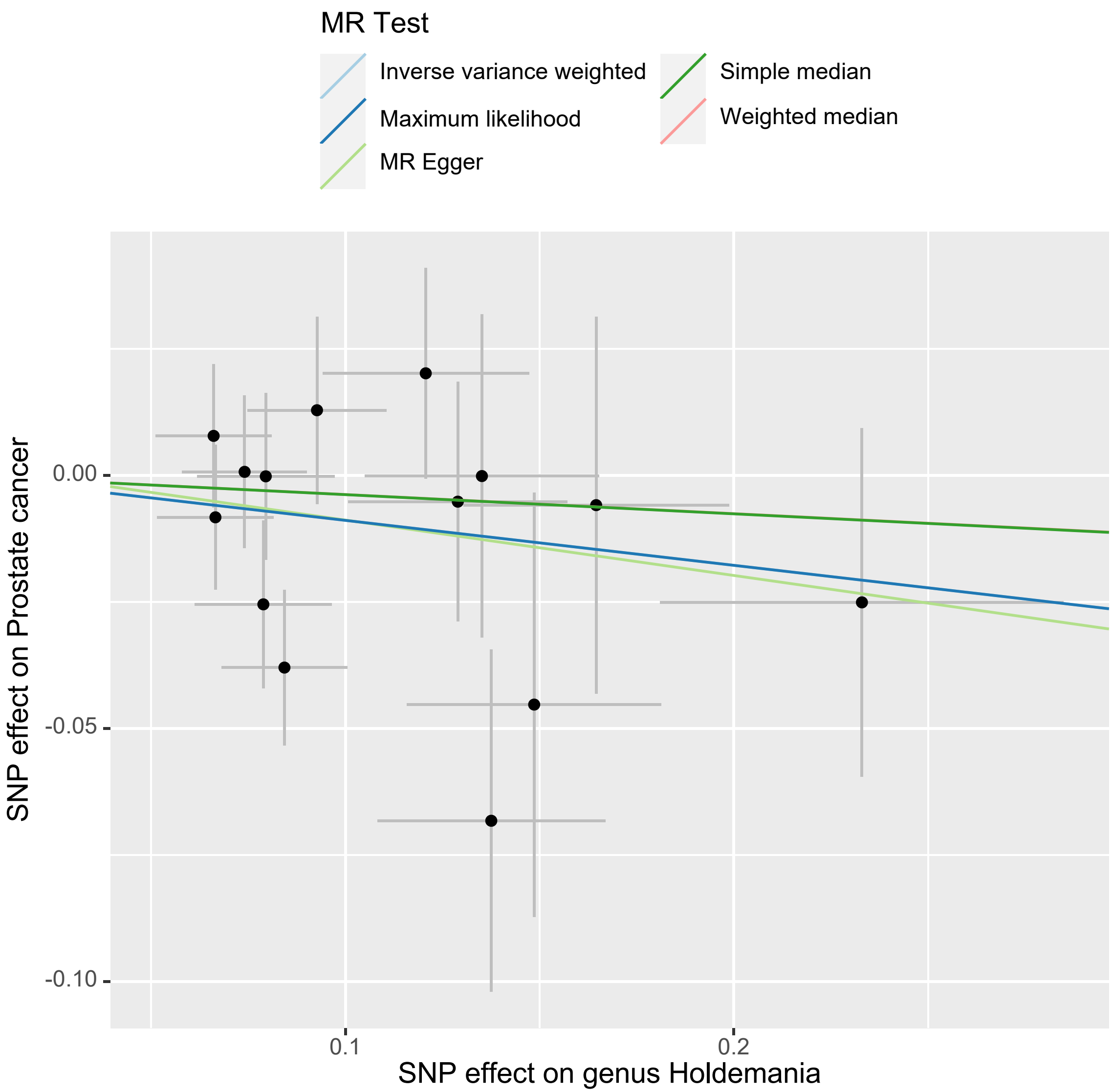

Figure S85. The scatter plot of genus Holdemania on prostate cancer in the FinnGen consortium

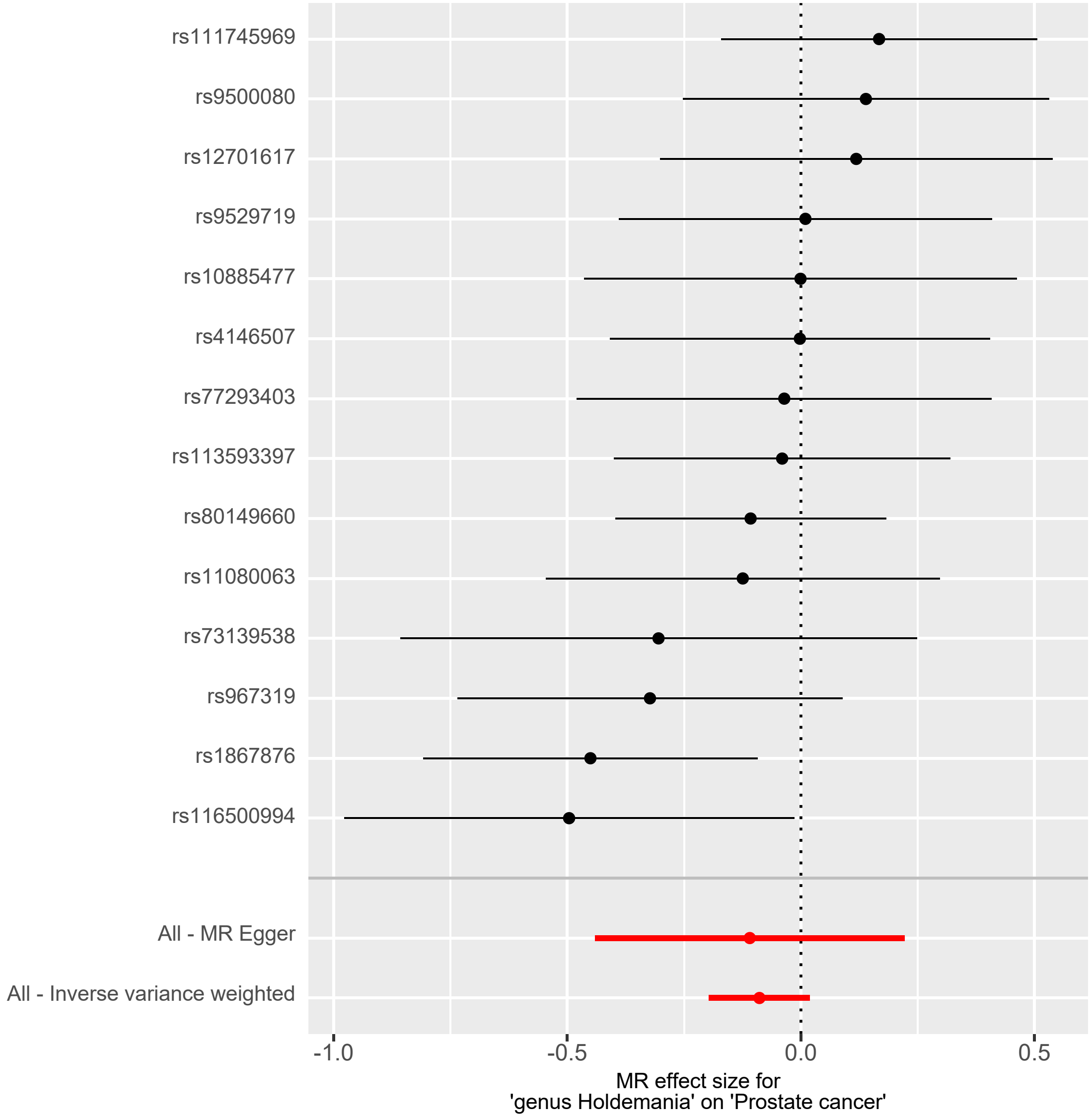

Figure S86. The forest plot of genus Holdemania on prostate cancer in the FinnGen consortium

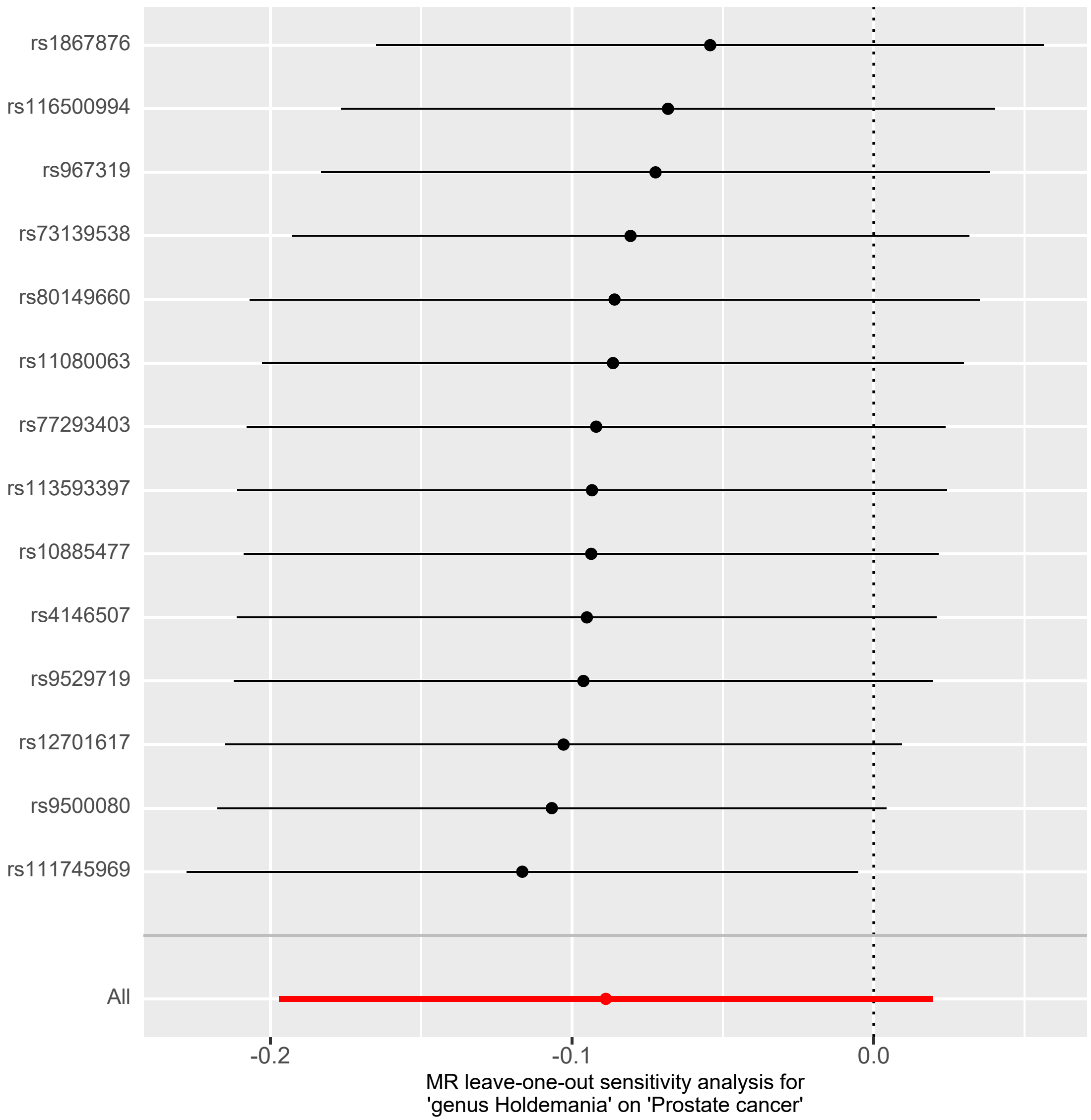

Figure S87. The leave-one-out test of genus Holdemania on prostate cancer in the FinnGen consortium

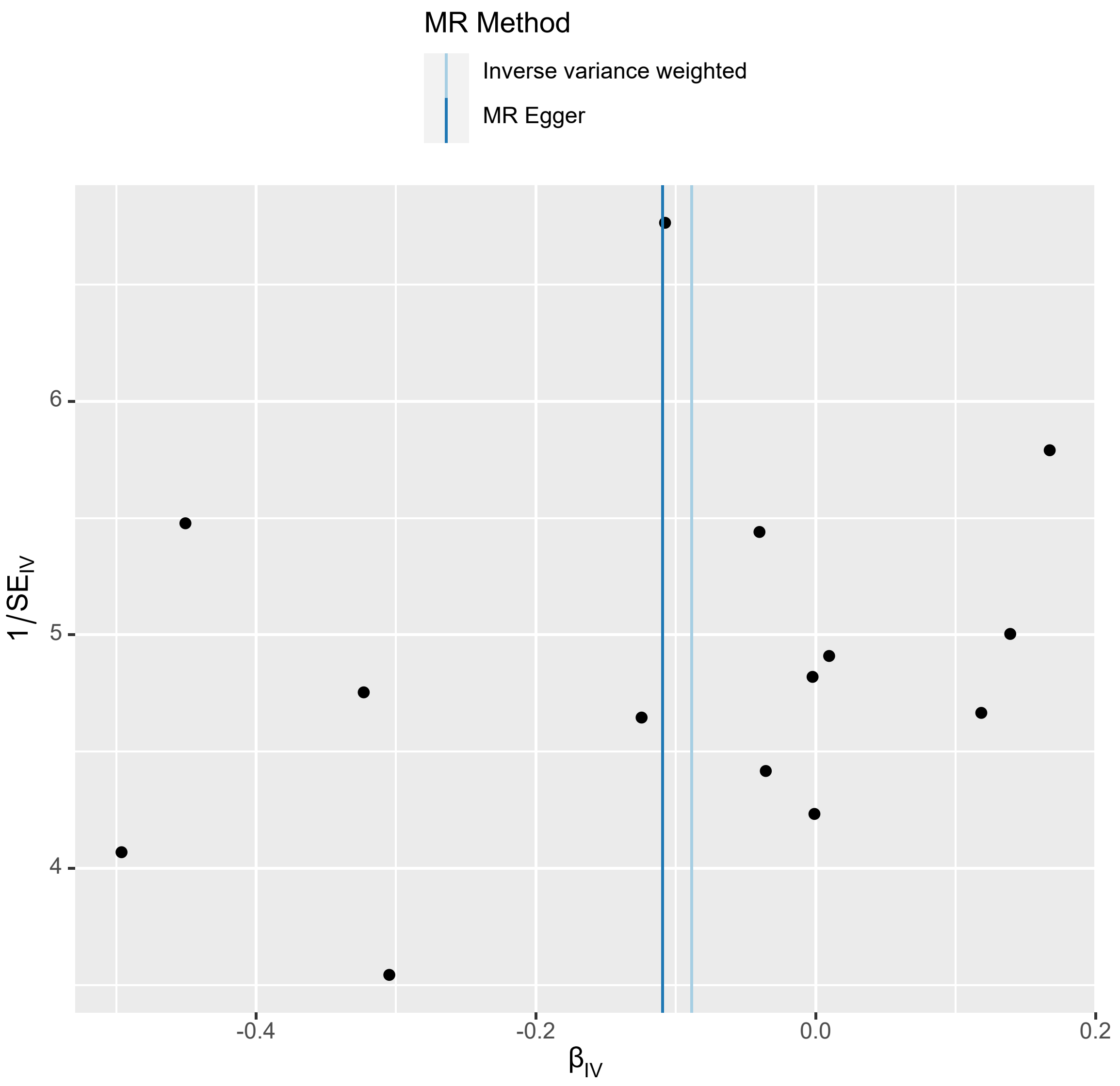

Figure S88. The funnel plot of genus Holdemania on prostate cancer in the FinnGen consortium

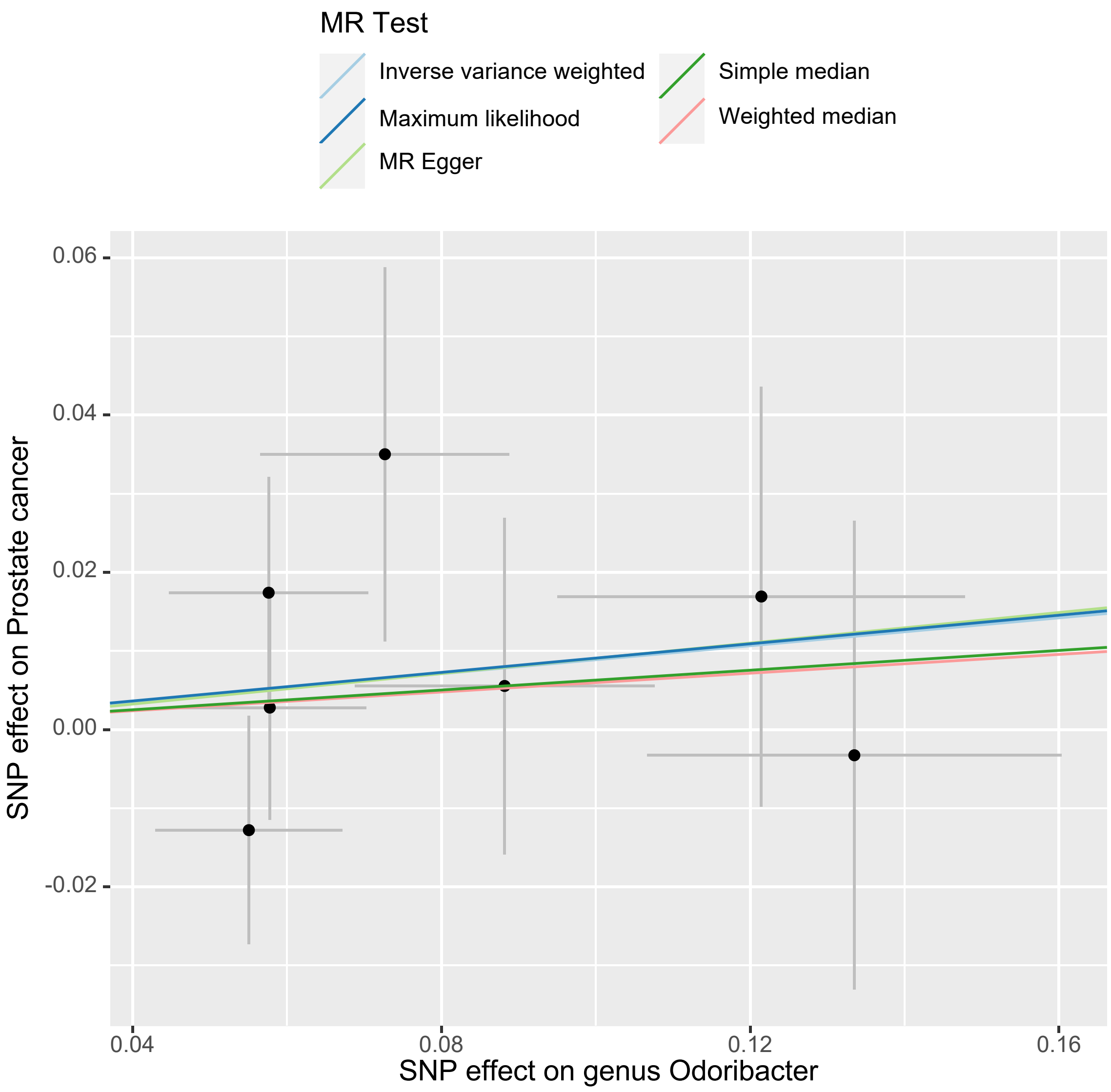

Figure S89. The scatter plot of genus Odoribacter on prostate cancer in the FinnGen consortium

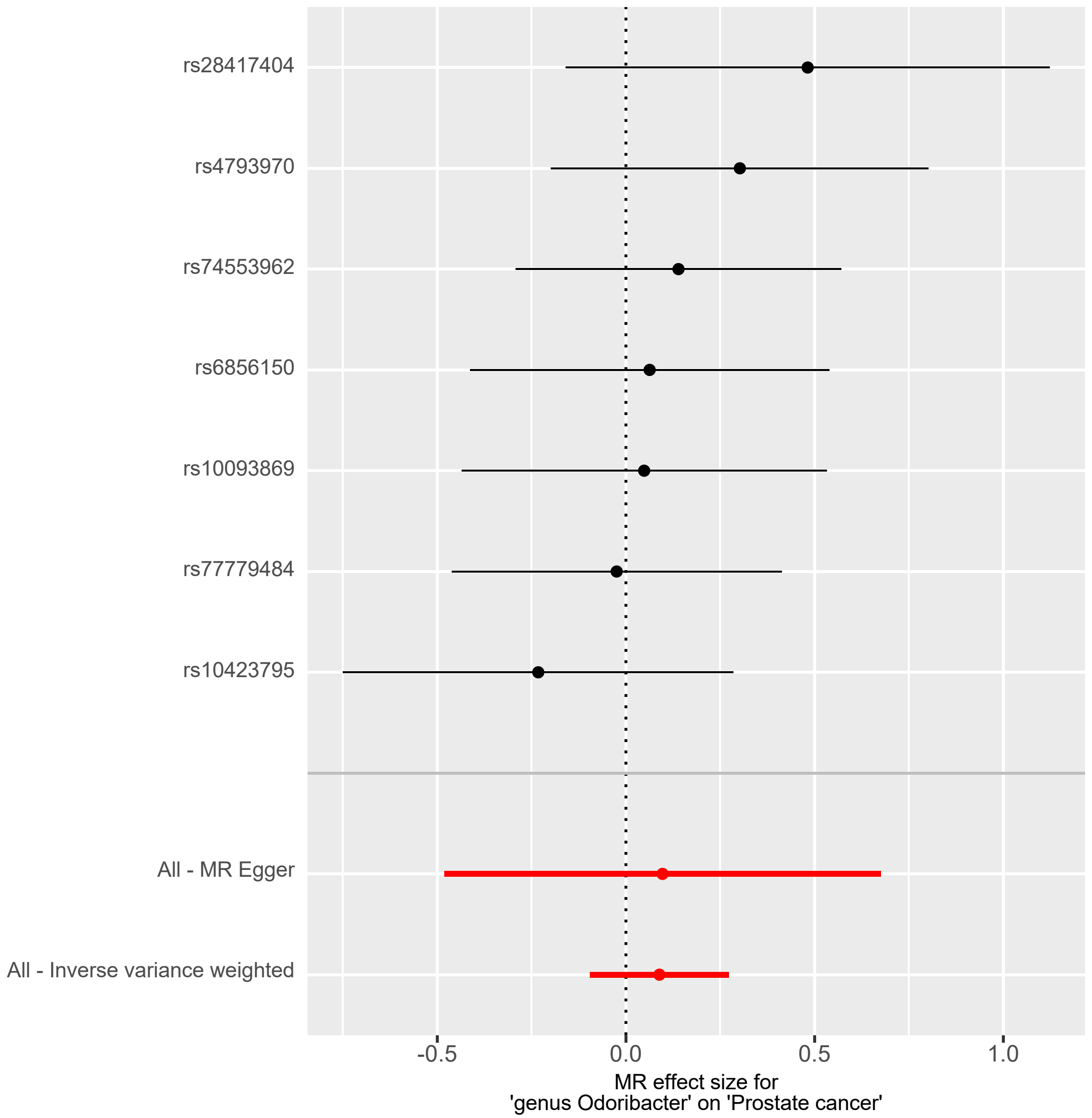

Figure S90. The forest plot of genus Odoribacter on prostate cancer in the FinnGen consortium

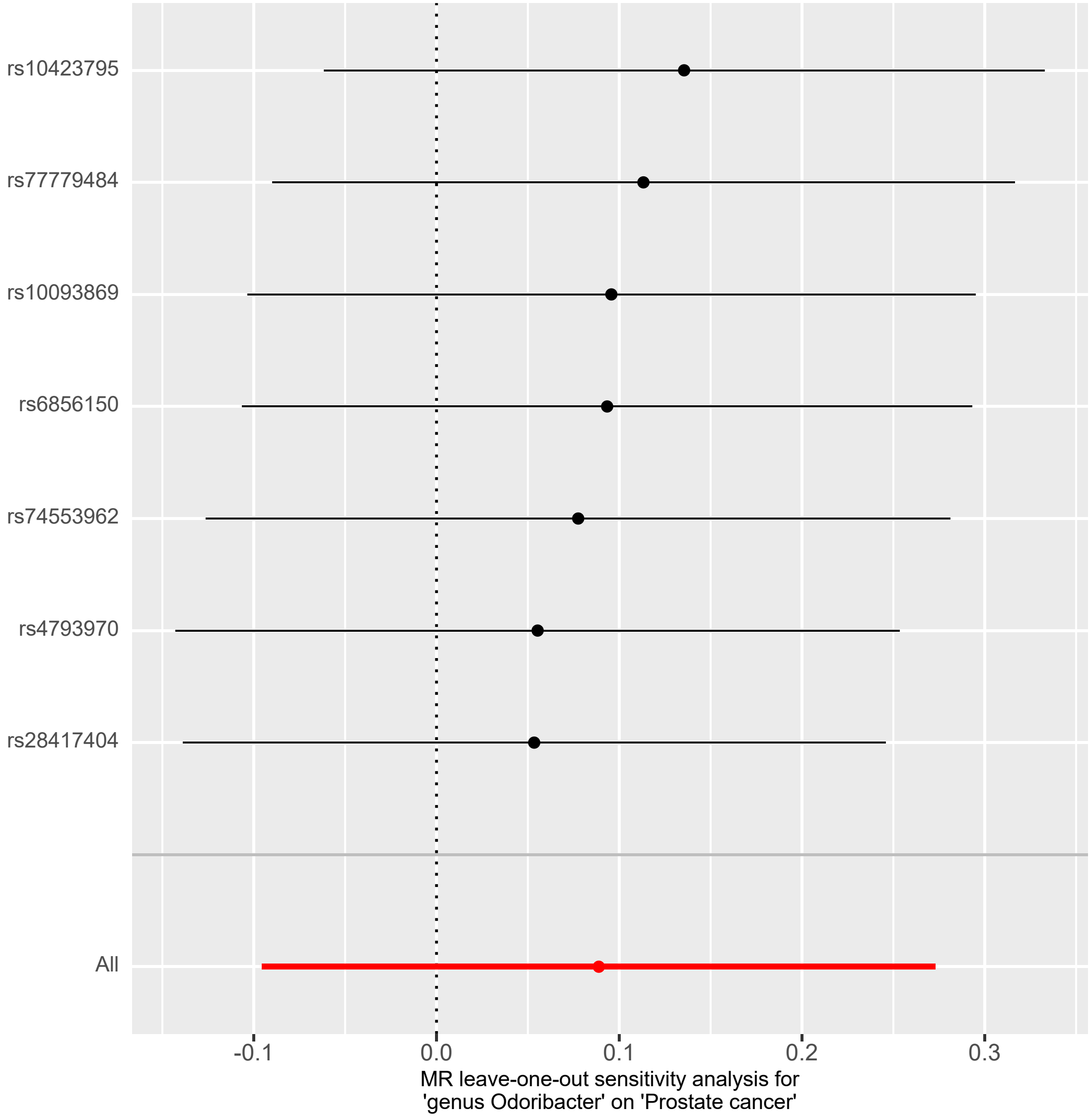

Figure S91. The leave-one-out test of genus Odoribacter on prostate cancer in the FinnGen consortium

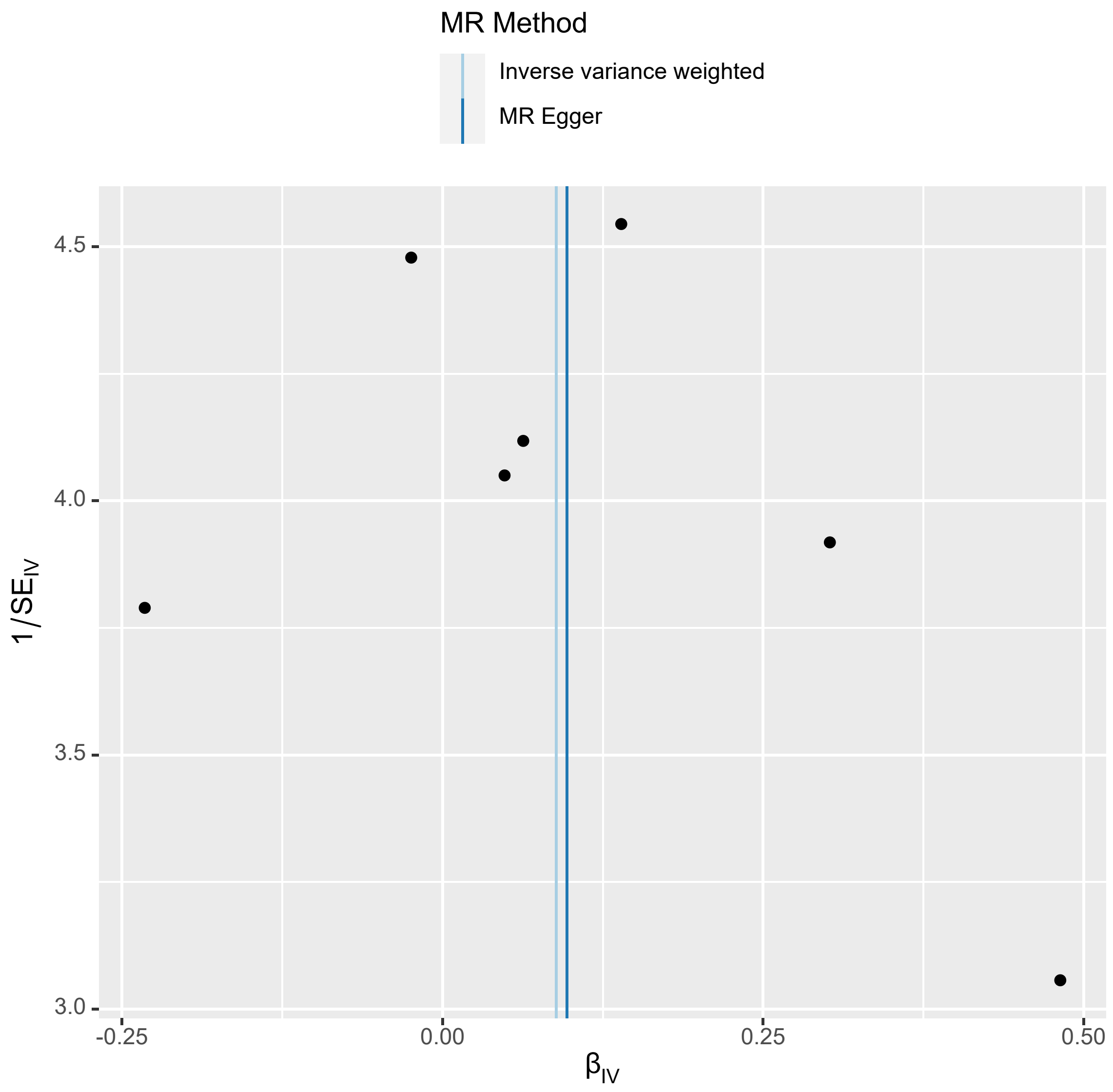

Figure S92. The funnel plot of genus Odoribacter on prostate cancer in the FinnGen consortium

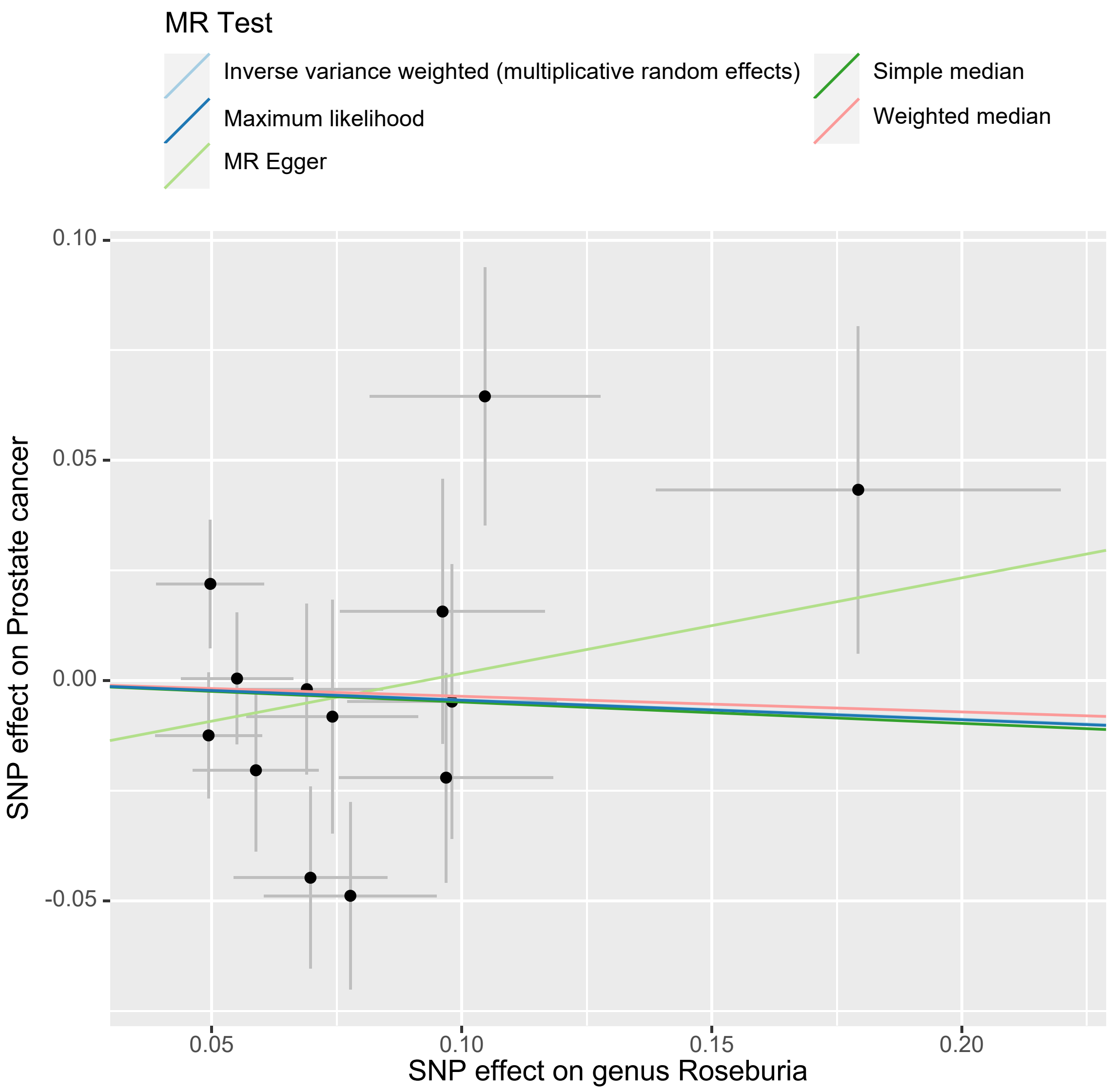

Figure S93. The scatter plot of genus Roseburia on prostate cancer in the FinnGen consortium

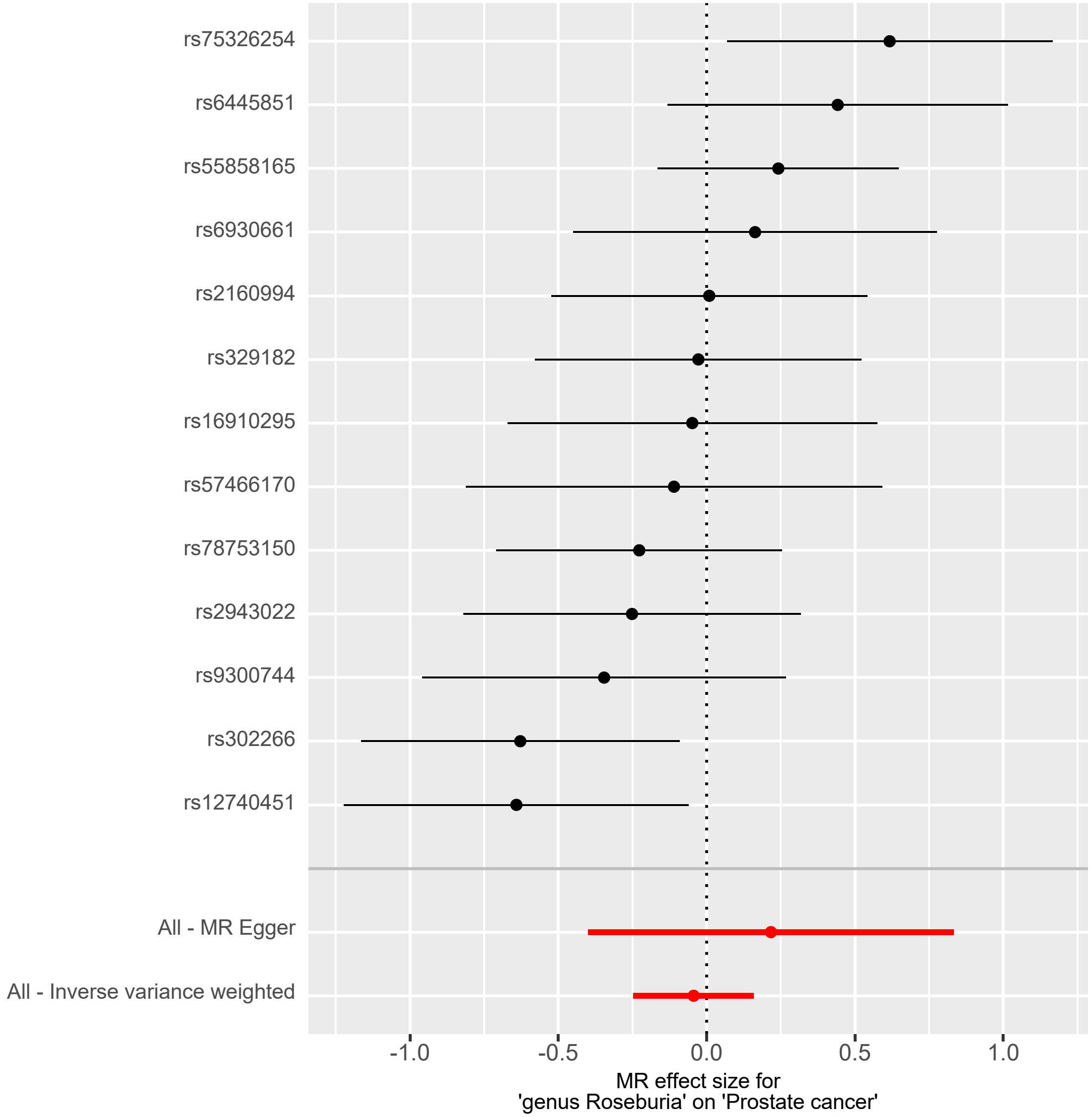

Figure S94. The forest plot of genus Roseburia on prostate cancer in the FinnGen consortium

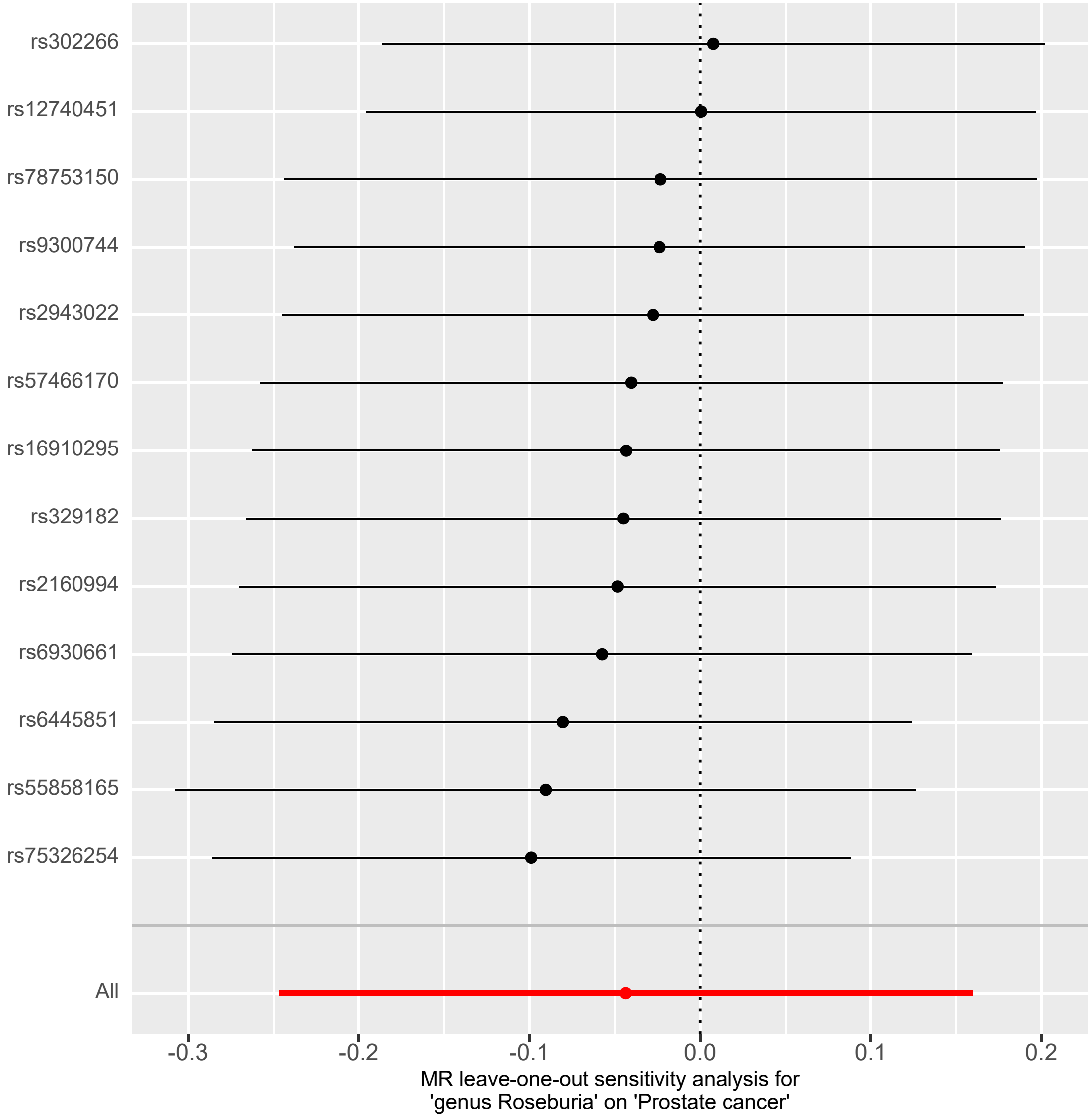

Figure S95. The leave-one-out test of genus Roseburia on prostate cancer in the FinnGen consortium

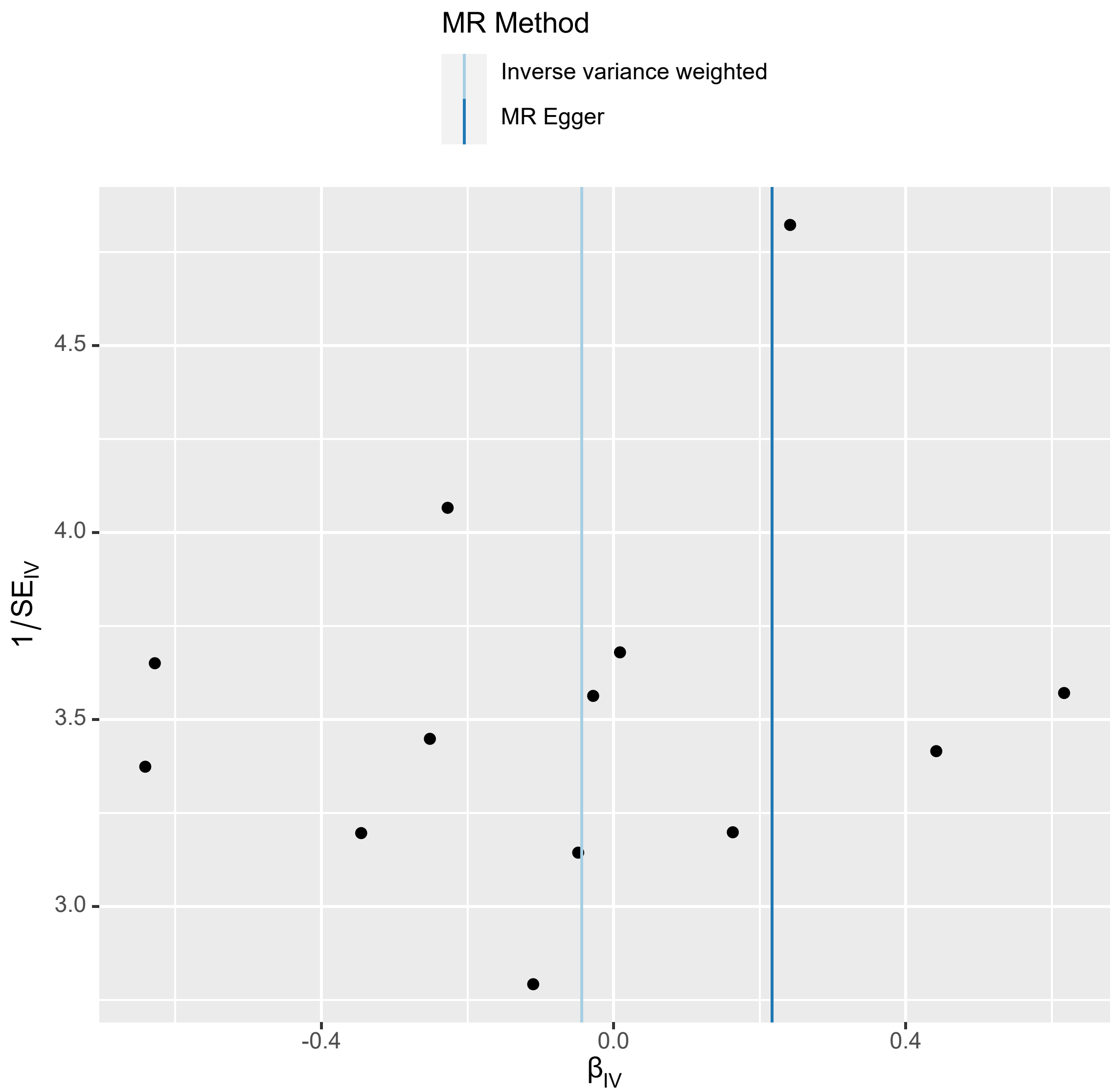

Figure S96. The funnel plot of genus Roseburia on prostate cancer in the FinnGen consortium

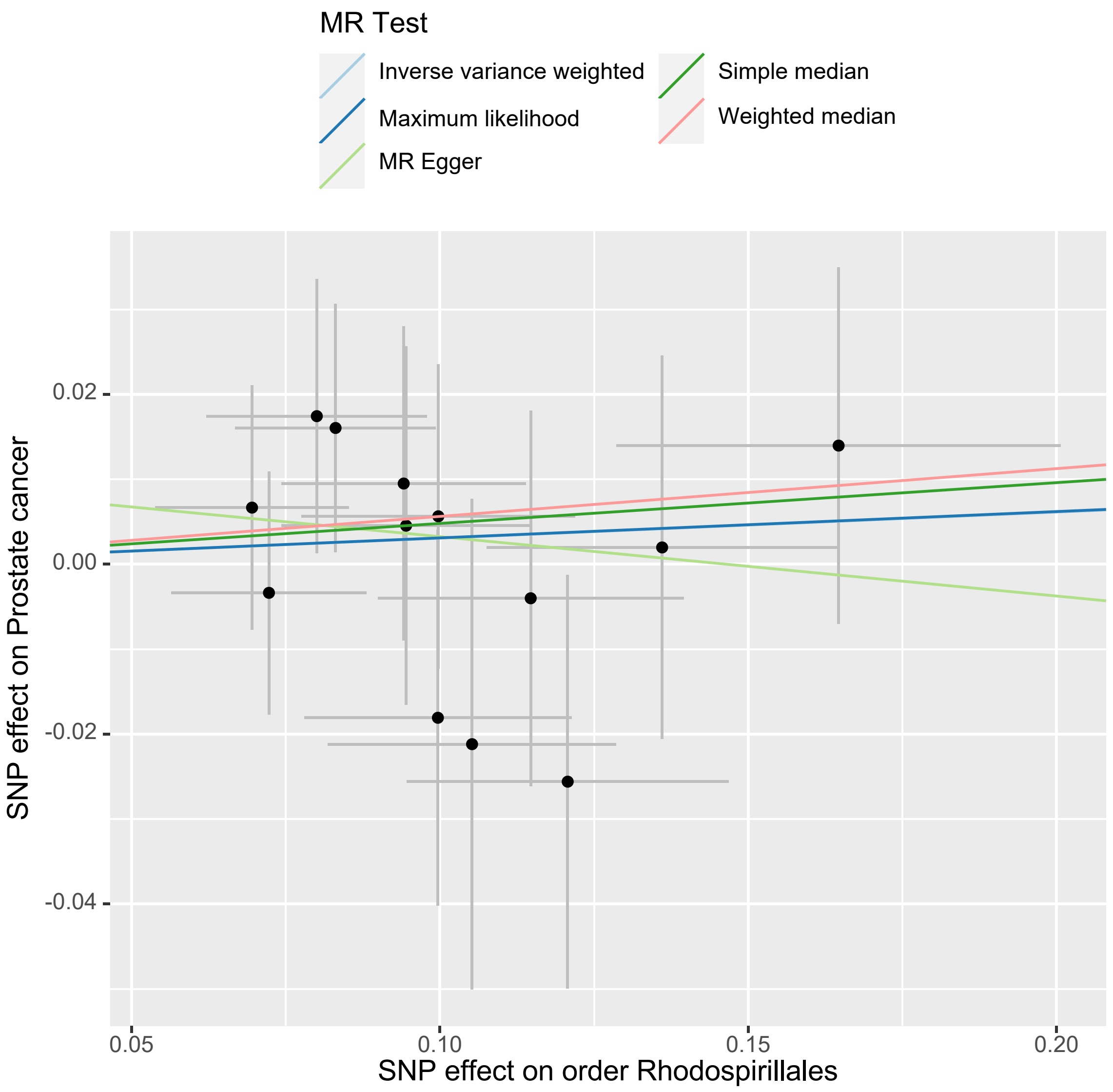

Figure S97. The scatter plot of order Rhodospirillales on prostate cancer in the FinnGen consortium

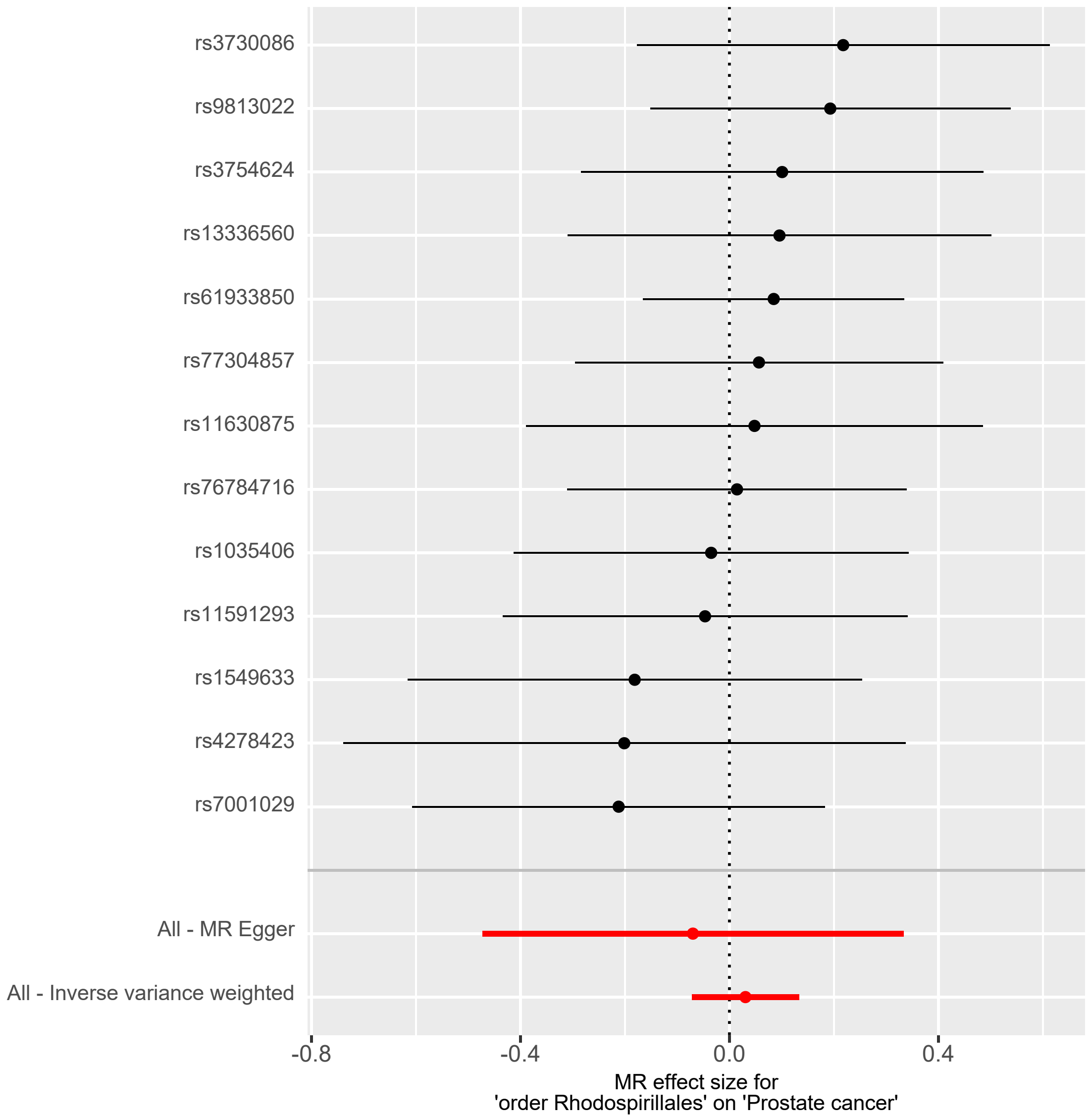

Figure S98. The forest plot of order Rhodospirillales on prostate cancer in the FinnGen consortium

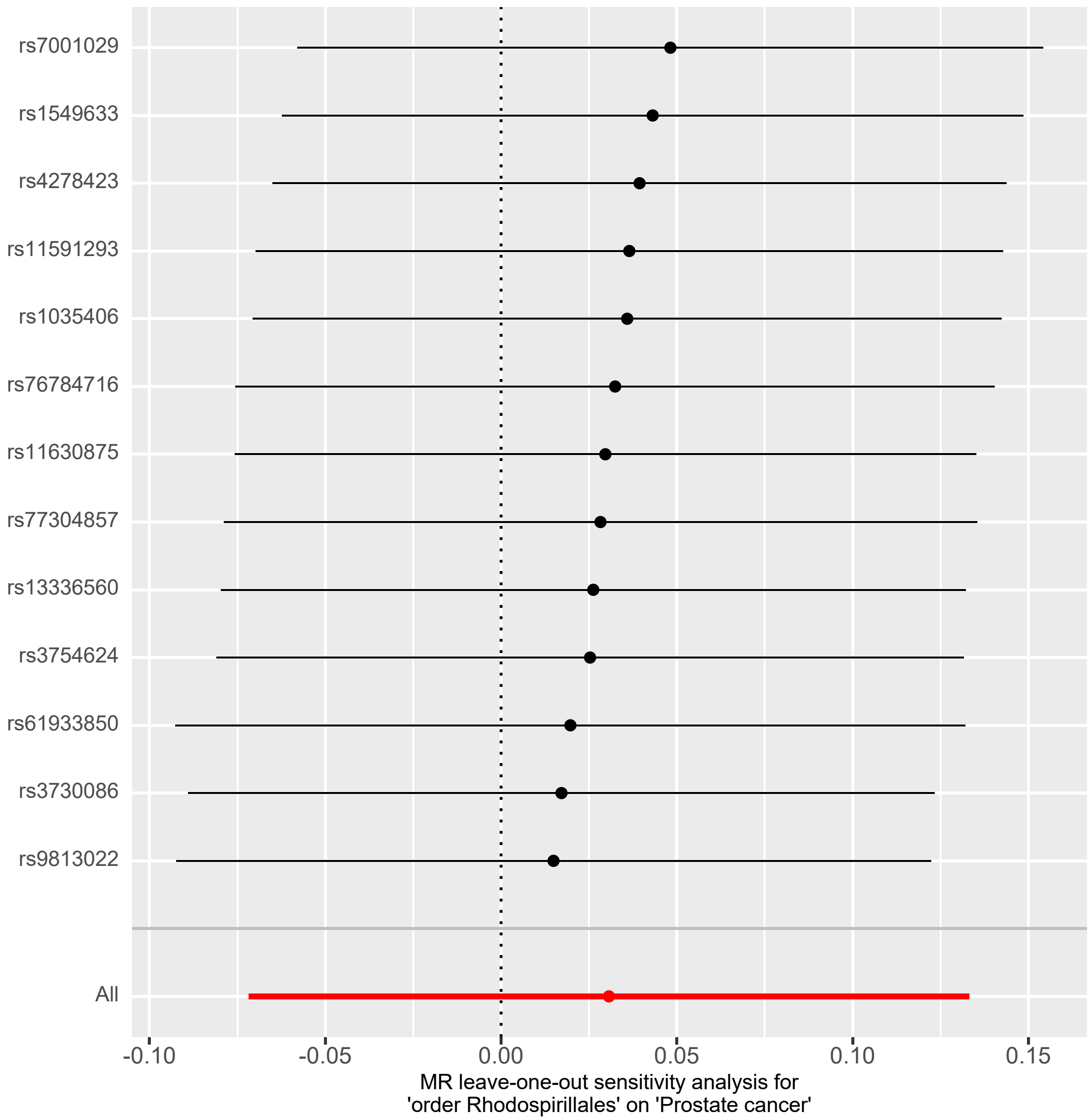

Figure S99. The leave-one-out test of order Rhodospirillales on prostate cancer in the FinnGen consortium

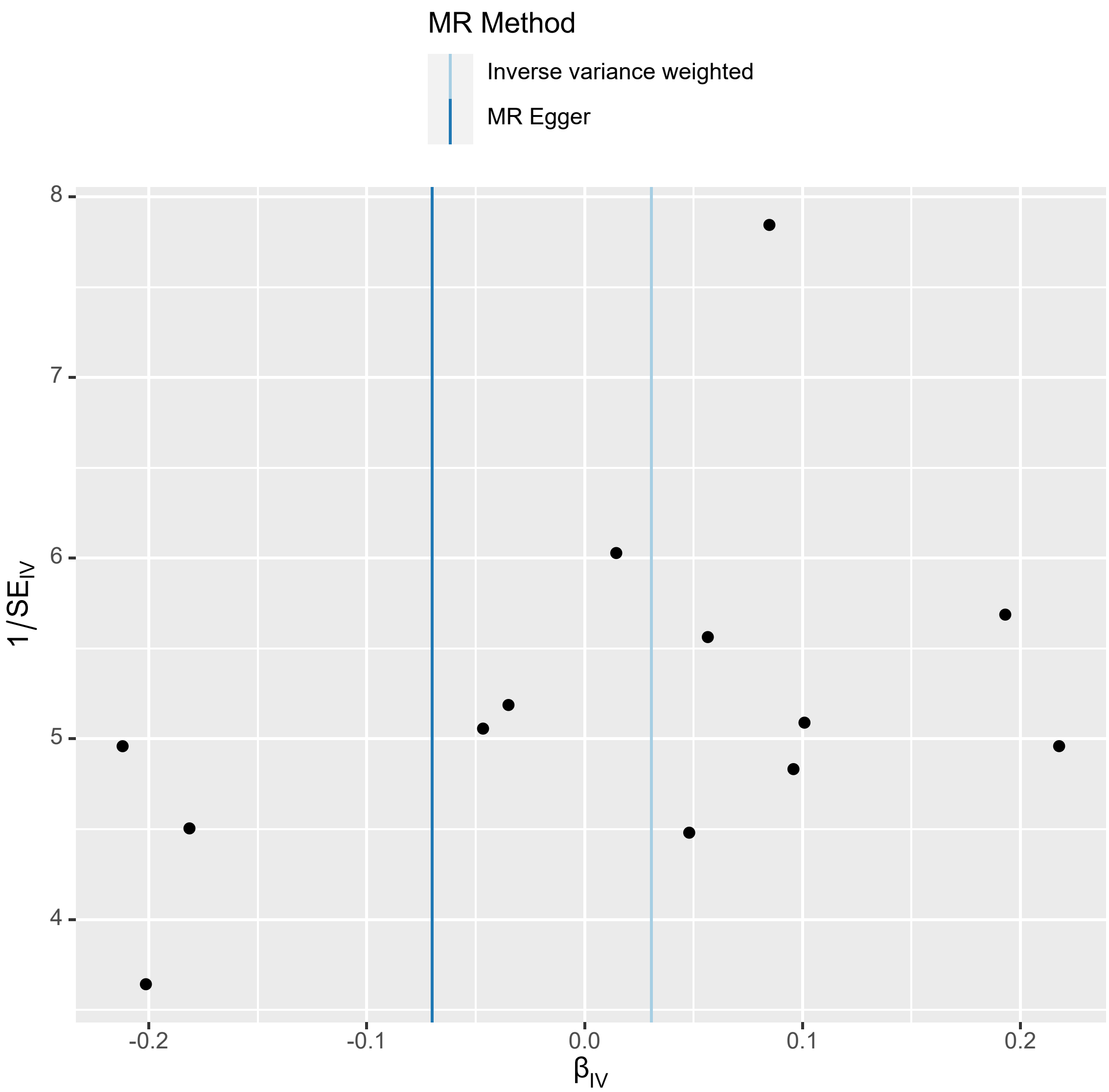

Figure S100. The funnel plot of order Rhodospirillales on prostate cancer in the FinnGen consortium

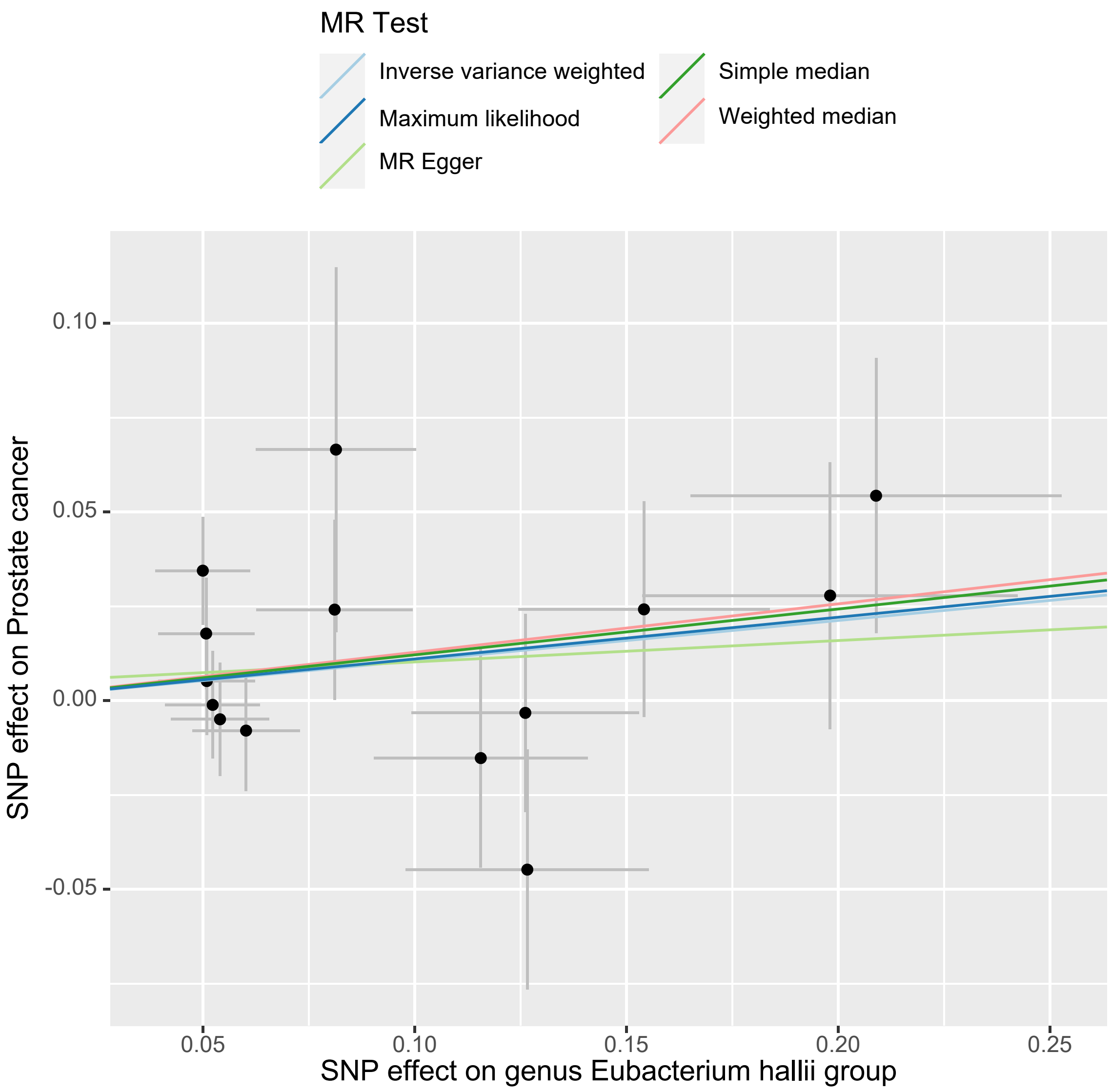

Figure S101. The scatter plot of genus Eubacterium hallii group on prostate cancer in the FinnGen consortium

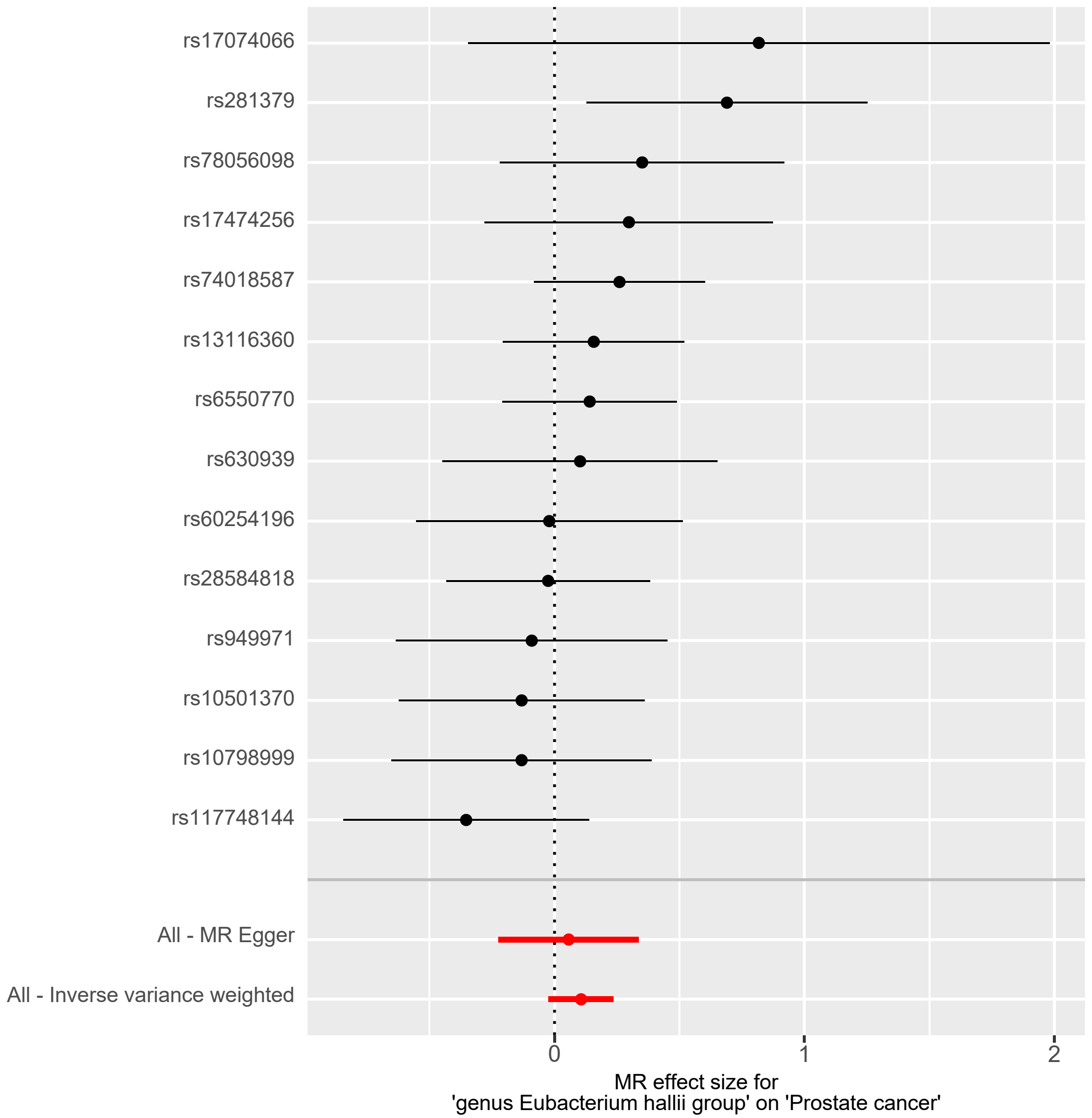

Figure S102. The forest plot of genus Eubacterium hallii group on prostate cancer in the FinnGen consortium

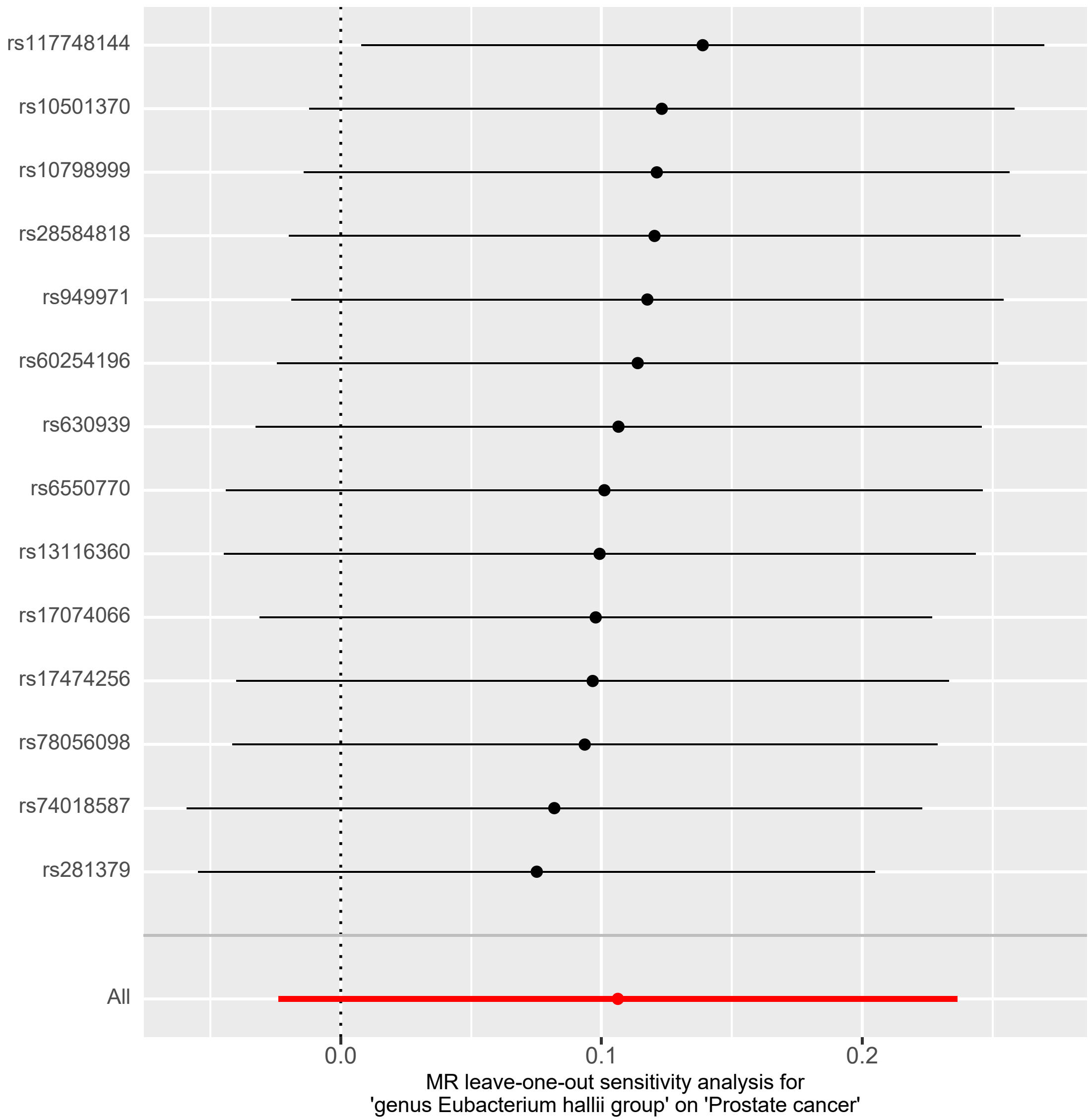

Figure S103. The leave-one-out test of genus Eubacterium hallii group on prostate cancer in the FinnGen consortium

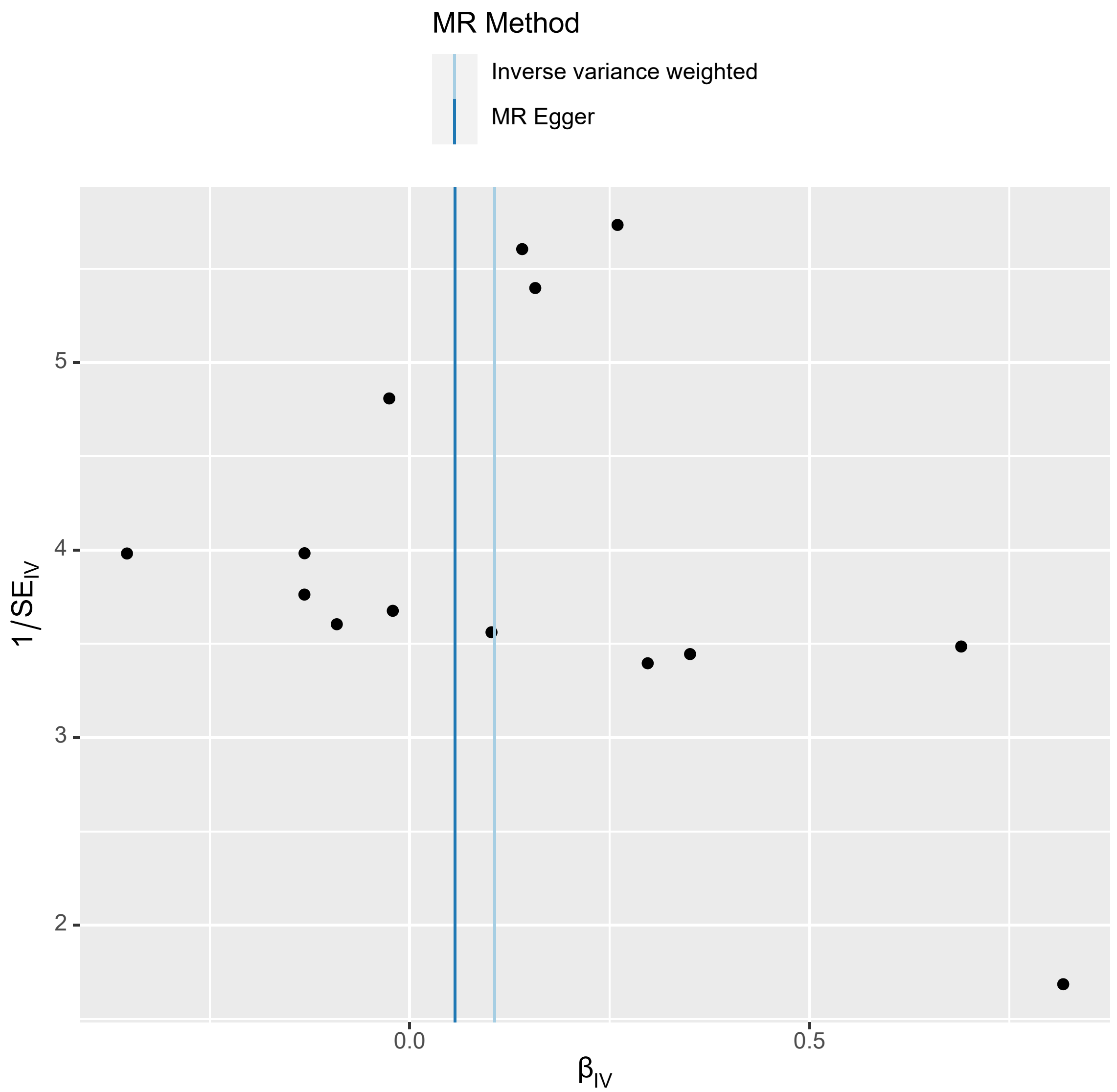

Figure S104. The funnel plot of genus Eubacterium hallii group on prostate cancer in the FinnGen consortium
